# Unpacking transatlantic income inequality

**DOI:** 10.1073/pnas.2625474123

**Published:** 2026-11-03

**Authors:** James P. Ziliak, Jonathan Cribb, Richard Blundell, James Banks, David Green, Antoine Bozio, Malka Guillot, Barra Roantree, Maxime To, Brindusa Anghel, Cristina Barceló, Jim Been, Patrick Bennett, Maximilian Blömer, Olympia Bover, Koen Caminada, Bart Capéau, Daniele Checchi, Svetoslav Danchev, André Decoster, Bram De Rock, Kelly Foley, Giorgos Gatopoulos, Martin Halla, Bradley Hardy, Elena Herold, Laura Hospido, Tullio Jappelli, Egbert Jongen, Niki Kalavrezou, Arizo Karimi, Elizabeth Krause, Max Lay, Soren Leth-Petersen, Charlotte Lucke, Immacolata Marino, Antonis Mavropoulos, Carlos Oliveira, Mårten Palme, Tuuli Paukkeri, Grigoris Pavlou, Andreas Peichl, Pedro Portugal, Lukas Puschnig, Pedro Raposo, Ann-Christin Rathje, Terhi Ravaska, Craig Riddell, Marja Riihelä, Johan Saeverud, Kjell G. Salvanes, Annalisa Scognamiglio, Anne Steuernagel, Jonas Vanderkelen, Heike Vethaak, Nikos Vettas, Andrea Weber, Thomas Wernham

**Affiliations:** ^a^ Institute for Fiscal Studies, London NW1 4DF, United Kingdom; ^b^ Department of Economics, University of Kentucky, Lexington, KY 40506; ^c^ Department of Economics, University College London, London WC1E 6BT, United Kingdom; ^d^ Department of Economics, University of Manchester, Manchester M13 9PL, United Kingdom; ^e^ Vancouver School of Economics, University of British Columbia, Vancouver, BC V6T 1LR, Canada; ^f^Institut des Politiques Publiques, Paris 75014, France; ^g^ Paris School of Economics, Paris 75014, France; ^h^ École des hautes études en sciences sociales, Paris 75006, France; ^i^ Department of Economics, Hautes Études Commerciales Liège, Université de Liège, Liège 4000, Belgium; ^j^ Department of Economics, Trinity College Dublin, The University of Dublin, Dublin 2, Ireland; ^k^ Banco de España, Madrid 28014, Spain; ^l^ Department of Economics, Leiden University, Leiden 2311 ES, The Netherlands; ^m^ Department of Economics, University of Liverpool, Liverpool L69 7ZH, United Kingdom; ^n^ Information und Forschung Institute for Economic Research, University of Munich, München 81679, Germany; ^o^ Centro de Estudios Monetarios y Financieros, Madrid 28014, Spain; ^p^ Faculty Governance & Global Affairs, Leiden University, The Hague 2511 VA, The Netherlands; ^q^ European Center for Advanced Research in Economics and Statistics, Université libre de Bruxelles, Bruxelles 1050, Belgium; ^r^ Department of Economics, Katholieke Universiteit Leuven, Leuven 3000, Belgium; ^s^ University of Milan, Milano 20122, Italy; ^t^Foundation for Economic and Industrial Research, Athens 117 42, Greece; ^u^ Department of Economics, University of Saskatchewan, Saskatoon, SK S7N 5A5, Canada; ^v^ Department of Economics, Vienna University of Economics and Business, Vienna 1020, Austria; ^w^ McCourt School of Public Policy, Georgetown University, Washington, DC 20001; ^x^ Department of Economics and Statistics and Centre for Studies in Economics and Finance, University of Naples Federico II, Napoli 80126, Italy; ^y^ European Centre for Social Welfare Policy and Research, Vienna 1090, Austria; ^z^ Department of Economics, Uppsala University, Uppsala 751 05, Sweden; ^aa^ Department of Economics and Center for Economic Behavior and Inequality, University of Copenhagen, Copenhagen DK-1353, Denmark; ^bb^ Department of Economics, Stockholm University, Stockholm 106 91, Sweden; ^cc^Valtion taloudellinen tutkimuskeskus, Helsinki 00101, Finland; ^dd^ Faculty of Economics, Ludwig-Maximilians-Universität München, München 80539, Germany; ^ee^ Economics and Research Department, Bank of Portugal, Lisboa 1100-150, Portugal; ^ff^ Nova School of Business and Economics, Carcavelos 2775-405, Portugal; ^gg^Católica Lisbon School of Business and Economics, Lisboa 1649-023, Portugal; ^hh^ Faculty of Management and Business, Tampere University, Tampere 33100, Finland; ^ii^ Department of Economics, Norwegian School of Economics, Bergen 5045, Norway; ^jj^ Faculty of Business, Economics, and Law, Friedrich-Alexander University Erlangen-Nuremberg, Nuremberg 90402, Germany; ^kk^ Department of Economics, Athens University of Economics and Business, Athens 10434, Greece; ^ll^ Department of Economics, Central European University, Vienna 1100, Austria; ^mm^ Austrian Institute of Economic Research, Vienna 1030, Austria

**Keywords:** disposable income inequality, redistribution, convergence

## Abstract

This paper uses newly harmonized survey and register data on incomes and labor market outcomes as the basis for consistent comparisons of inequalities across 17 North American and European countries over the period 1995–2021. We examine trends in disposable income inequality in the working-age population, defined as persons aged 25 to 60, as well as its component parts of market income inequality and redistribution through government tax and transfer systems. We adjust incomes to allow for changes in education, population aging, and household structure in each country. We test for convergence across countries in demographically-adjusted disposable income inequality, along with market income inequality and redistribution. There is strong evidence of convergence over time in disposable income inequality across the 17 countries, despite a divergence in the dispersion of market income inequality driven by very heterogeneous trends for hours of work and the number of workers in households across countries. Consistent with this we find a strong convergence across countries in the dispersion of our measure of redistribution. There are reductions over time in inequality-reducing redistribution in the Nordic countries and increases in some other European and North American countries, with the net effect being more similarity across countries, a lower average level of redistribution, and a higher average level of disposable income inequality. These trends are being driven by the bottom 95% of the income distribution. Our results are robust to a variety of alternative specifications including the measure of inequality, trimming of data outliers, and sample period.

## Significance

From 1995–2021 there was a convergence in disposable income inequality across advanced North American and European countries and a rising average level of disposable income inequality. This occurred despite inequality in market incomes becoming less similar across countries due to diverse changes in hours of work and the number of workers at the household level. The convergence in disposable income inequality was due to convergence across countries in the inequality-reducing redistribution that tax and transfer systems do, with a trend toward less redistribution on average over time, due to policy changes and also the natural interactions between tax and transfer systems and the distribution of market incomes. Increasing inequality in the Nordic countries is an important part of this story.

Income inequality in rich countries has been one of the most widely watched barometers of economic well-being among policymakers in recent decades and has generated intense research interest (1–33). However, the extent to which there have been common trends, or diverging experiences, in inequality across a broad range of high-income countries remains understudied (34–48). Understanding which trends and patterns are country-specific and which are shared is useful in assessments of common economic shocks from financial crises such as the Great Recession of 2008–2009 or health crises like COVID-19 in 2020–2021, as well as of secular developments in labor markets from technological change and globalization. Such comparisons are also helpful to understand whether redistributive policies and institutions designed to reduce inequality within a country spread across countries, and whether such policies and institutions work in concert to attenuate or increase inequality.

This paper uses newly harmonized survey and register data on household incomes and labor market outcomes as the basis for consistent comparisons of inequalities across 17 North American and European countries over the last three decades. The harmonized microdata, which we developed in parallel with the IFS Deaton Review (49–52), ensures comparability across countries, focusing on the period between 1995 and 2021 (*Materials and Methods*). These data are complementary to, and expand upon, existing international data provided by the OECD (53), the LIS (54), the WID (55), and the GRID (56). Some of the existing data provide various estimates of inequality for a subset of our outcomes, but not the underlying micro data, while others, notably the LIS, provide micro data but for fewer years and without the rich administrative data available in some of our countries. In short, those existing resources, while highly valuable, do not provide all the detailed information we need to unpack all the stages of the income process that leads from hourly wages to redistribution undertaken by the tax and transfer systems over the last 30 y.

We begin our analysis by examining broad trends in disposable income inequality, as well as its component parts of market income inequality and redistribution. For our purposes, market income is the sum of labor-market earnings, ordinary capital income such as interest and dividends, and private transfer income not provided by the government. Redistribution refers to government tax payments and receipts of transfer income, where tax payments include direct income taxes at the federal level (including Social Security payroll taxes) as well as the state (province/district) level, inclusive of refundable tax credits that could make tax liability negative, while income transfers include both cash and near-cash transfers like food and housing assistance. We exclude indirect taxes on consumption such as the value-added tax (VAT) and in-kind transfers such as health insurance. Disposable income is thus the sum of market income and redistribution, which is higher or lower than market income depending on whether the household is a net recipient of government transfers or net payer of taxes.

We add up the resources contributed by all members of the household to construct our measures of household disposable and market incomes, but for our analysis we focus at the individual level on the population who is predominantly reliant on labor income: the working-age population of 25 to 60 y olds. This is accomplished by equivalizing household-level disposable and market incomes whereby we adjust income for differences in household size and age composition of children, and thus incomes used in measuring inequality are on a per-person basis (*Materials and Methods*). The implication is that a married or cohabiting couple where each partner is in the 25 to 60 age range will appear in our sample as separate persons with the same level of equivalized resources. The age restriction captures the part of the life course after formal schooling is completed for most individuals, and prior to retirement, and where labor-market inequalities most directly feed into disposable income inequalities. Secular changes in education attainment, population aging, and household structure among the working-age population in recent decades may affect both pretax (market) inequality via who selects into work, as well as posttax and transfer (disposable) inequality through differential treatment of households in tax and transfer programs by presence of children.

This leads to a second innovation of our paper, in that we adjust our inequality comparisons for changing demographics in each country using Recentered Influence Functions (RIF; See *Materials and Methods* for details and *SI Appendix*, Figs. S5–S10 for a full set of analyses based on the unadjusted data). This is a more robust method of controlling for changes in the demographic composition of countries than the standard least-squares residual adjustment found in scores of papers on wage inequality (1–3, 7, 9, 11–15) as it allows the demographics to have a different effect across the unconditional distribution of income, instead of assuming a constant (average) effect throughout (57).

Moving beyond descriptive trends, we then formally test whether our set of countries are converging toward a common (higher or lower) level of income inequality over time (*Materials and Methods*). Testing whether the average income levels of poorer countries are catching up to average levels of richer countries is a standard application of neoclassical growth theory models (58–61). Similar tests of whether the within-country dispersion of incomes is also converging are much less common (38–40, 45), even though convergence in distribution is a prediction of those same growth models (62, 63). We first test whether there is convergence in disposable income inequality, and then separately whether inequality convergence is occurring across market incomes, from income redistribution, or both (or neither). Within market incomes, we also test for convergence in hourly wage inequality, individual earnings inequality, and equivalized household earnings inequality to help disentangle whether changes in labor-market inequality come from changes in hourly wage inequality, in individual hours of work, or the number of workers in the household.

Estimating the extent of income redistribution undertaken through tax and transfer systems across so many economies over time, along with conducting tests of convergence at each of these stages, is a third innovation of our paper. We then decompose the change in disposable income inequality over time into the contributions of market income inequality and of redistribution in order to better understand whether drivers of inequality change stem from changes in labor markets, or changes in the effects of government tax and transfer systems on inequality (47).

## Results

**Fig. 1.**
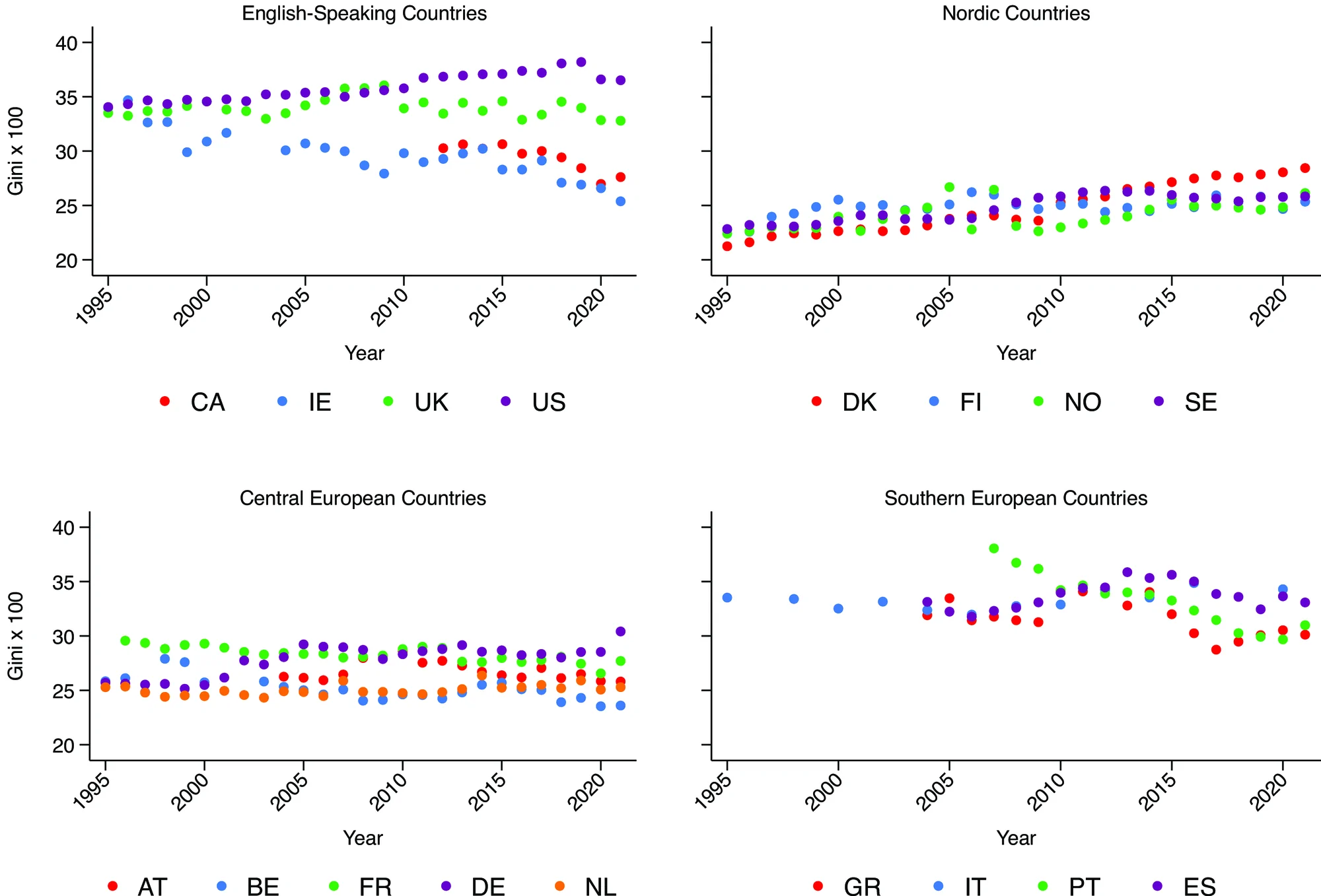
Cross-country trends in disposable income inequality among the working-age population. Inequality is measured by the Gini coefficient times 100. Income includes contributions from household members of all ages, and is equivalized using the Modified OECD scale. The sample used in the figure is the working-age population defined as those aged 25 to 60. Countries are grouped into English-speaking nations of Canada (CA), Ireland (IE), United Kingdom (UK), and United States (US); Nordic countries of Denmark (DK), Finland (FI), Norway (NO), and Sweden (SE); Central European countries of Austria (AT), Belgium (BE), France (FR), Germany (DE), and The Netherlands (NL); and Southern European nations of Greece (GR), Italy (IT), Portugal (PT), and Spain (ES).

Fig. 1 presents trends in disposable income inequality among the working-age population for the 17 countries in our sample spanning calendar years 1995–2021. Inequality is measured by the widely used Gini coefficient times 100, where a value of 0 represents no inequality and a value of 100 represents complete inequality. The Gini coefficient captures the entire distribution, and it is unit free, and thus permits meaningful comparisons across countries over time with income measured in different currencies (See *SI Appendix* and discussion below in the *Robustness* section for alternative inequality measures). In the figure, countries are grouped into English-speaking countries of Canada (CA), Ireland (IE), United Kingdom (UK), and United States (US) in the *Upper-Left* panel; Nordic countries of Denmark (DK), Finland (FI), Norway (NO), and Sweden (SE) in the *Upper-Right* panel; Central European countries of Austria (AT), Belgium (BE), France (FR), Germany (DE), and The Netherlands (NL) in the *Lower-Left* panel; and Southern European countries of Greece (GR), Italy (IT), Portugal (PT), and Spain (ES) in the *Lower-Right* panel.

The key takeaway in Fig. 1 is that there is substantial heterogeneity in experiences of disposable income inequality in the working-age population in recent decades. Indeed, there are as many countries with declining inequality as increasing. The United States has consistently the highest level of disposable income inequality, and it has trended upward over time. We also see rising inequality in the Nordic countries, most notably Denmark and Sweden, and Norway to a lesser extent, though importantly these increases are from much lower levels compared to the US. On the other hand, Ireland and Portugal stand out as having relatively high levels of inequality that have fallen substantially over time, while Canada, France, Greece, and Spain have experienced more tempered declines in recent years. The remaining countries have experienced remarkable stability in Gini inequality, though the levels of inequality in Southern Europe consistently exceed Central Europe.

**Fig. 2.**
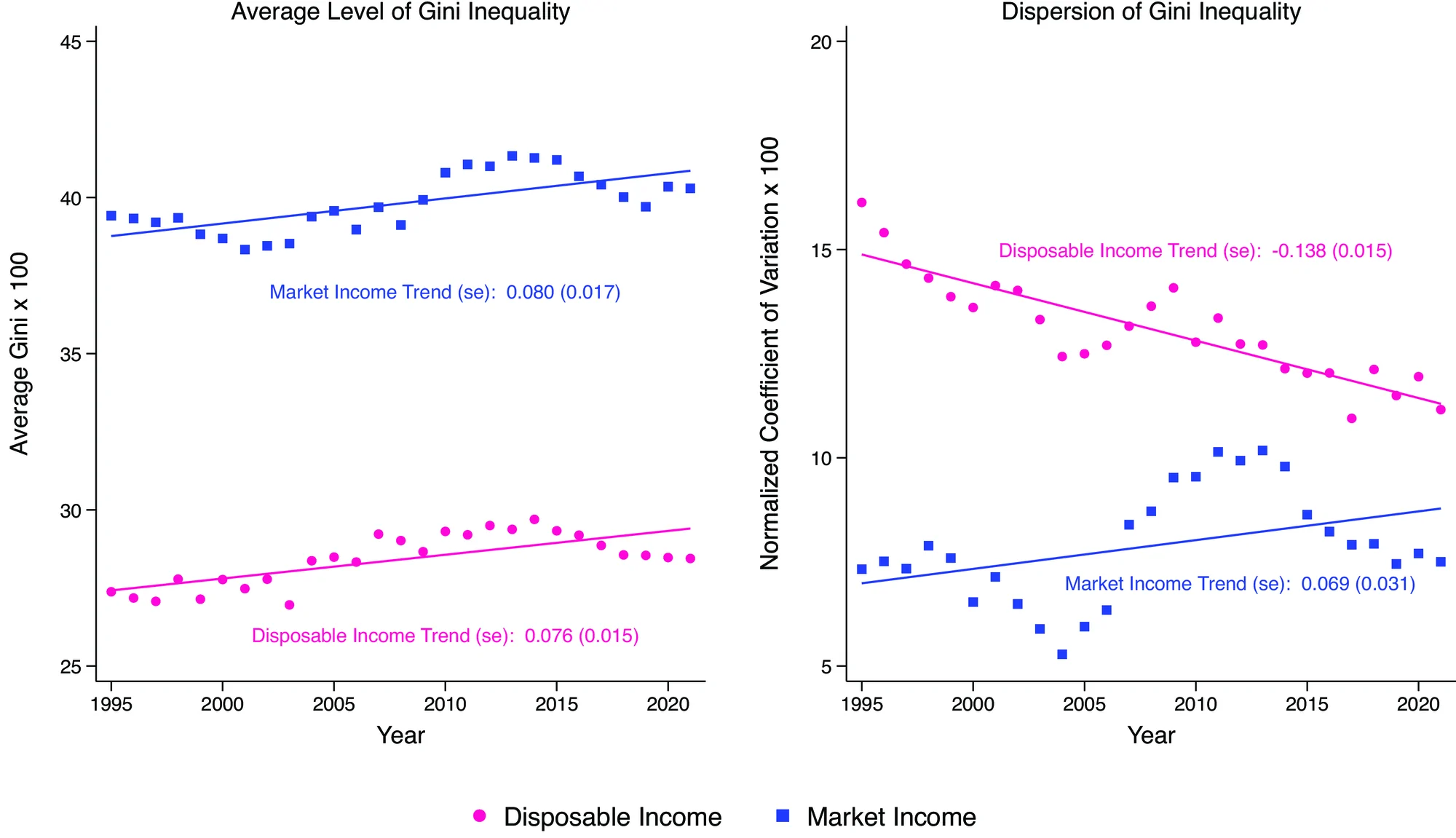
Average level of and convergence in cross-country disposable and market income inequality among the working-age population. Inequality is measured by the Gini coefficient times 100. The test of convergence is represented by the coefficient and associated SE on the linear trend lines in the *Right*-hand panel. Income includes contributions from household members of all ages, and is equivalized using the Modified OECD scale. Working-age population is defined as those aged 25 to 60.

The *Left* panel of Fig. 2 displays the average disposable- and market-income Gini coefficient across countries in each year, along with a linear trend line with associated coefficient and SE in parentheses. The figure shows that despite the heterogeneity of inequality displayed in Fig. 1, on average inequality has been increasing over time. We see much higher levels of inequality in market incomes that are also increasing over time. The higher average market income inequality is not emanating from one or a few countries, rather as depicted in *SI Appendix*, Fig. S1 market income inequality is higher than disposable income inequality in each country and year. Ireland stands out as having especially high market income inequality during and after the Great Recession, while there is notably more variation across the Nordic and Southern European countries compared to Fig. 1 on disposable income inequality. These level differences between market and disposable income inequality suggest that tax and transfer systems substantially reduce inequality on average, but given the common trend increase across income measures in Fig. 2, redistribution has not been successful in undoing the secular rise in average inequality.

The *Right* panel of Fig. 2 presents our main tests of convergence in cross-country income inequality. Because our interest is in changes in the dispersion of cross-country inequality over time, we focus on so-called *sigma*-convergence (See *Materials and Methods* and *SI Appendix*, section 9 for an alternative test of *beta*-convergence). Our measure of dispersion is the normalized coefficient of variation (CV) of the Gini coefficient times 100, which is bounded between 0 and 100 with decreasing dispersion as the normalized CV approaches 0 and increasing dispersion as the measure approaches 100. We overlay a linear trend with the corresponding coefficient and SE in parentheses. The panel shows that cross-country dispersion in disposable income inequality falls by nearly 30 percent over the sample period, signaling strong evidence of convergence as reflected in the statistically significant negative trend coefficient. On the other hand, we find evidence that *divergence* is occurring across countries in terms of market income inequality, though the rate is about one-half the size based on the smaller trend coefficient (in absolute value), in part due to the spike in dispersion in market-income inequality during the Great Recession years. Notable also is that the level of cross-country dispersion of Gini inequality in most years is higher with disposable income than market income, suggesting wide variation in redistribution across countries as we demonstrate below.

**Fig. 3.**
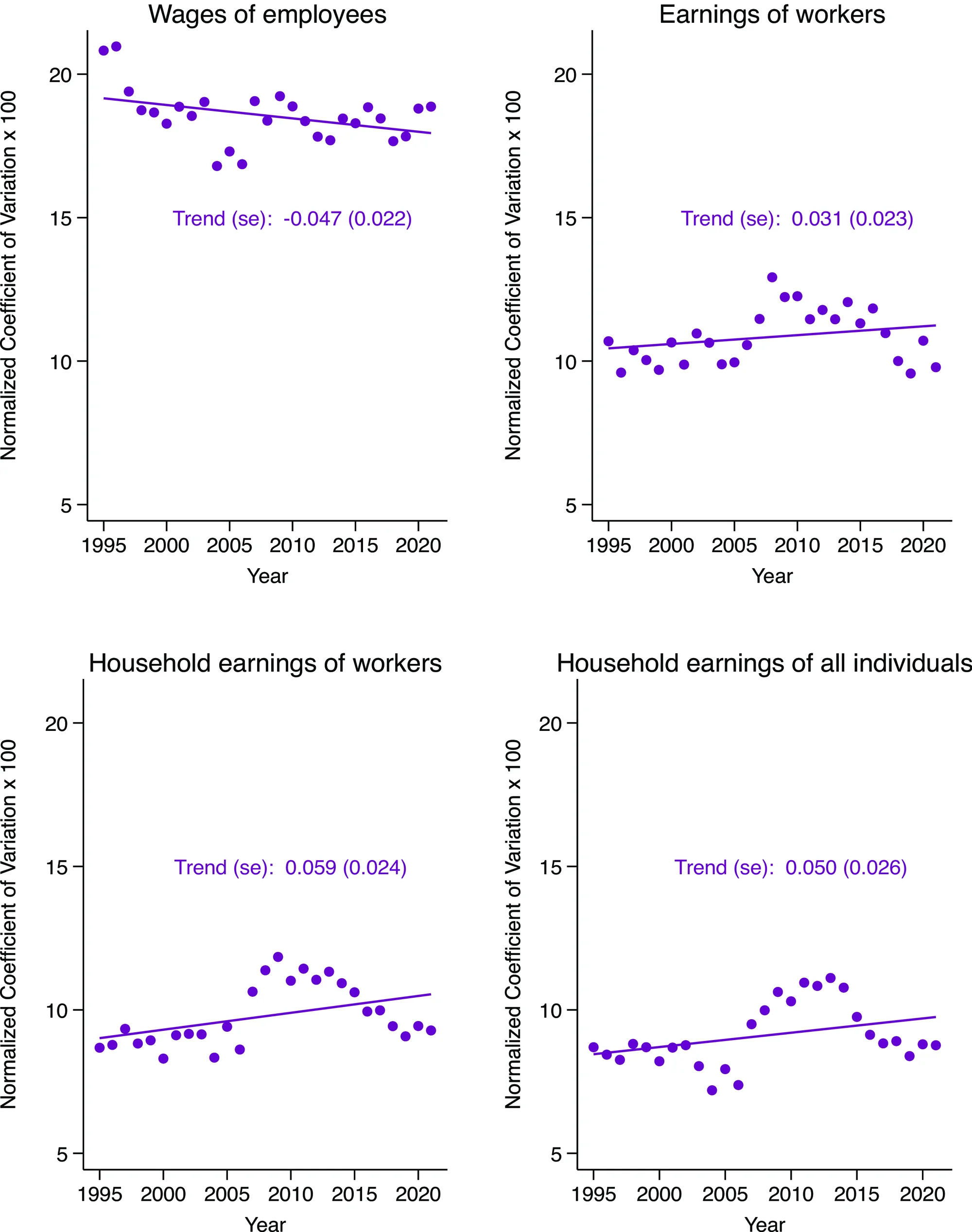
Tests of convergence in cross-country labor-market inequalities among working-age population. Inequality is measured by the Gini coefficient times 100. The test of convergence is represented by the coefficient and associated SE on the linear trend line. Wages of employees are average hourly earnings for employees. Earnings of workers are annual earnings of employees and self-employed workers. Household earnings of workers add up annual earnings of all persons who are employees or self-employed workers. Household earnings of all individuals include nonworking households. Household earnings are equivalized using the Modified OECD scale. Working-age population is defined as those aged 25 to 60.

Fig. 3 explores whether the cross-country divergence in market income inequality is coming from the labor market, and if so, at which stage of the earnings process. Specifically we test for convergence in inequality in hourly wages among employees (*Upper Left* panel), in individual earnings among both employees and the self-employed (*Upper Right* panel), in equivalized household earnings among employees and the self-employed (*Lower Left* panel), and in equivalized household earnings among all individuals (*Lower Right* panel). The latter group includes both those employed and those not employed, which means those households with no earnings are also included. Adding ordinary capital income and nongovernmental transfers to this last measure of household earnings with zeros included yields market income in Fig. 2.

Moving from the *Upper Left* panel of dispersion of hourly wage inequality in Fig. 3 to the *Upper Right* panel of dispersion of earnings inequality helps inform whether incorporating hours of work and the self-employed affects convergence in inequality. The *Lower* panels help to additionally reveal whether incorporating the number of household workers affects convergence. The figure shows that hourly wage inequality is converging across countries, even though (like disposable income inequality) average hourly wage inequality is increasing across countries (*SI Appendix*, Fig. S2). However, this does not feed through to earnings inequality, which is diverging, especially once we move to household earnings. This suggests that differences in the number of workers per household, and assortative matching across workers with different earnings levels, across countries underlies divergence in market income inequality (*SI Appendix*, Fig. S3). Further analysis indicates that wage inequality convergence is occurring primarily in the bottom half of the wage distribution, while household earnings divergence is both an upper- and lower-tail phenomenon (*SI Appendix*, Fig. S4).

**Fig. 4.**
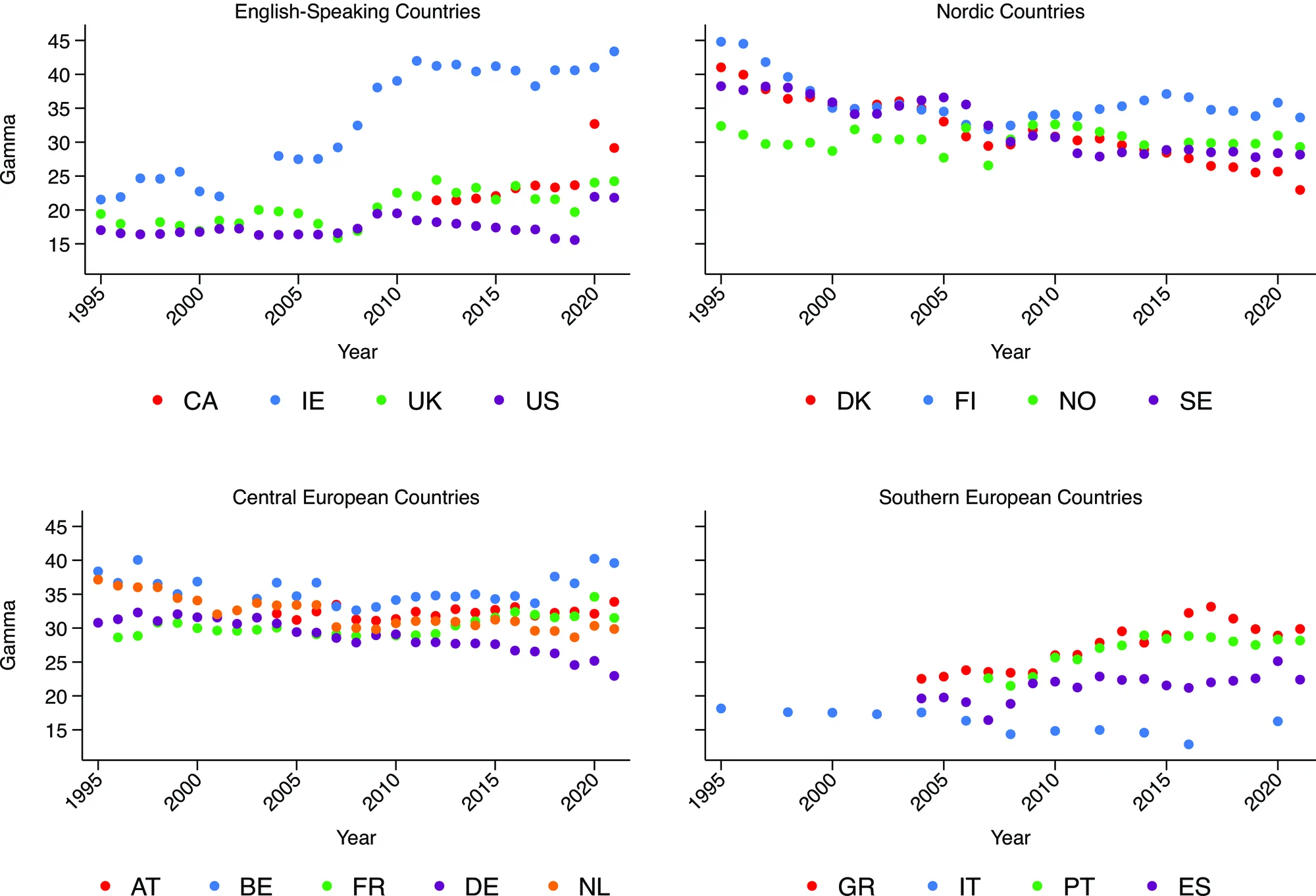
Cross-country trends in redistribution from market income to disposable income for working-age population. Inequality is measured by the Gini coefficient. *Gamma* is 100 times the relative reduction in market income inequality to disposable income inequality. Income includes contributions from household members of all ages, and is equivalized using the Modified OECD scale. Working-age population is defined as those aged 25 to 60.

Fig. 4 quantifies the extent of the effects of redistributive income tax and transfer policies by country and year. Each point labeled as *Gamma* on the y-axis of the graph represents the percentage reduction in Gini inequality in moving from market income to disposable income (times 100), with higher values corresponding to greater effects of redistribution (*Materials and Methods*). Note that since we hold demographic composition constant in our income series, these redistributive effects result from either changes in government tax and transfer policies, changes in how market inequality interacts with those policies, or both. The figure reveals a great deal of variation across countries, both within each of the four panels as well as across groupings of countries.

Among the English-speaking nations, Ireland stands out with very high levels of redistribution, with a substantial increase of about 15 percentage points in the years surrounding the Great Recession that persisted for the rest of the sample period. Canada and the US, and the UK to a lesser extent, had robust increases in redistribution during the COVID-19 pandemic, but otherwise had very different trends prior to the pandemic, with Canada trending upward and the UK and US trending downward. Among the Nordic countries, Denmark and Sweden, and also Finland, had levels of redistribution in the 1990s comparable to post-Great Recession Ireland, but then began a three-decade retreat, with redistribution according to this metric declining by nearly 40 percent in Denmark by the early 2020s. The Central European nations have similarly high levels of redistribution both to each other and to the high-redistribution Finland, Ireland, and Norway. However, in the years leading up to the Great Recession, the effects of redistribution in the Netherlands gradually fell, while Germany saw redistribution fall after the Great Recession. Among Southern European countries, Greece and Portugal provide redistribution rates comparable to Central European nations, whereas Italy provides much lower levels that are comparable to the US, with Spain in between.

**Fig. 5.**
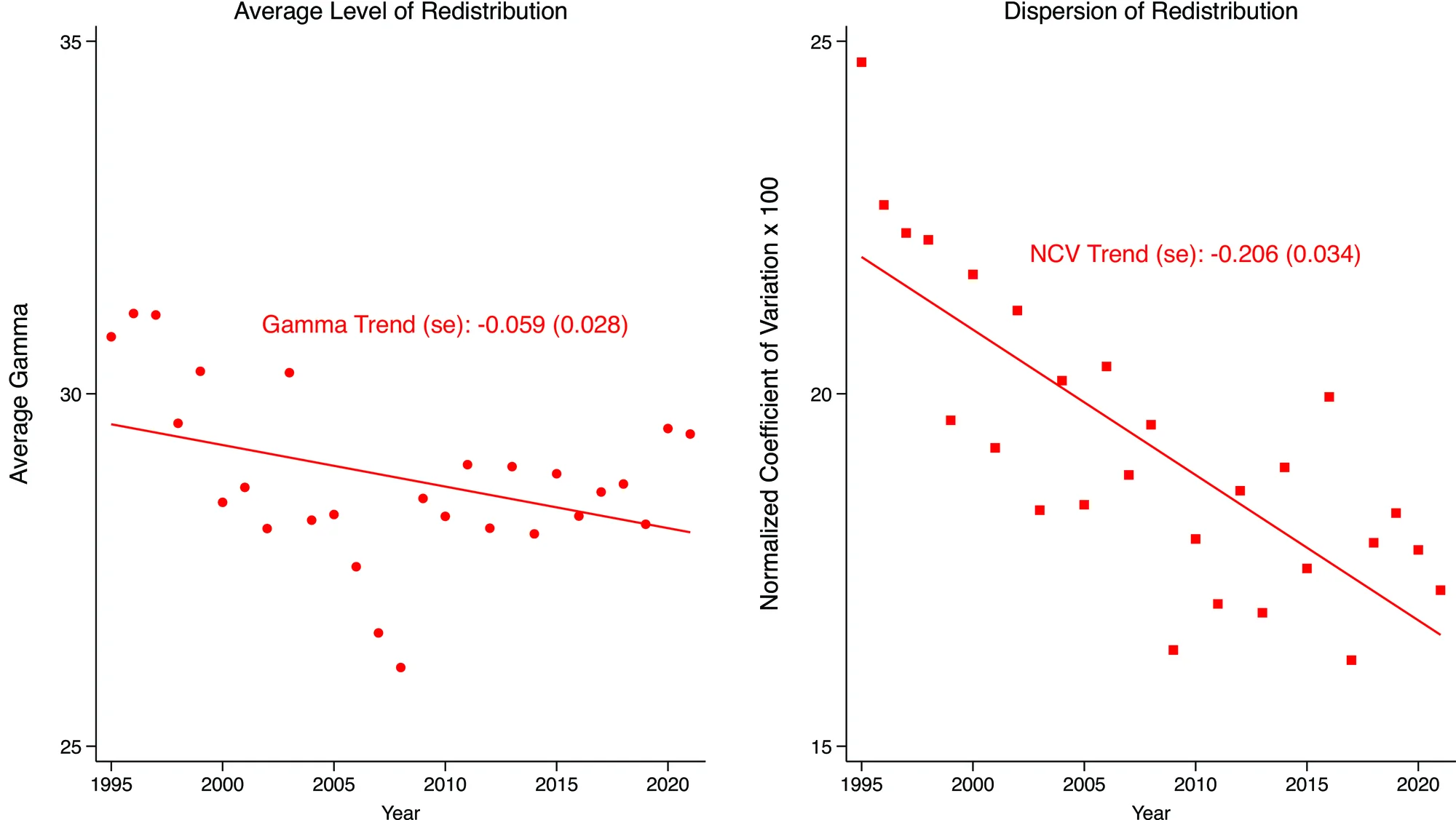
Average levels of and convergence in cross-country redistribution from market income to disposable income among the working-age population. Average Gamma is the cross-country average of Gamma in Fig. 4, the latter defined as 100 times the relative reduction in market income inequality to disposable income inequality as measured by the Gini coefficient. The test of convergence is represented by the coefficient and associated SE on the linear trend line in the *Right*-hand panel. Income includes contributions from household members of all ages, and is equivalized using the Modified OECD scale. Working-age population is defined as those aged 25 to 60.

Fig. 5 presents the estimates of how average effects of redistribution have changed over time in the *Left* panel, while in the *Right* panel we present estimates of dispersion in redistribution. In each panel, we depict the corresponding linear trend and associated SE. We measure dispersion as the normalized coefficient of variation of *Gamma* (times 100). In this context, *sigma*-convergence in redistribution effects would correspond to a decline over time in the cross-country dispersion of Gamma. The evidence in Fig. 5 indicates there has been convergence toward a lower level of redistribution, and this is strongly statistically significant.

Standard models of growth are silent on whether we should expect convergence in political economy decisions involving redistributive tax and transfer policies (62), and indeed a common finding in the growth literature is that convergence in income levels does not occur until after controlling for country-specific differences in institutions (60, 61). Nonetheless, the evidence in Figs. 4 and 5 suggests that historical institutional differences in the effect of tax and transfer programs are declining among this group of advanced economies. Notably, convergence is happening from both directions—the effects of redistribution in some high market-income inequality countries are increasing while the lowest market-income inequality countries with the largest redistributive effects (e.g., the Nordics) are becoming less so—and on average redistribution is declining.

**Fig. 6.**
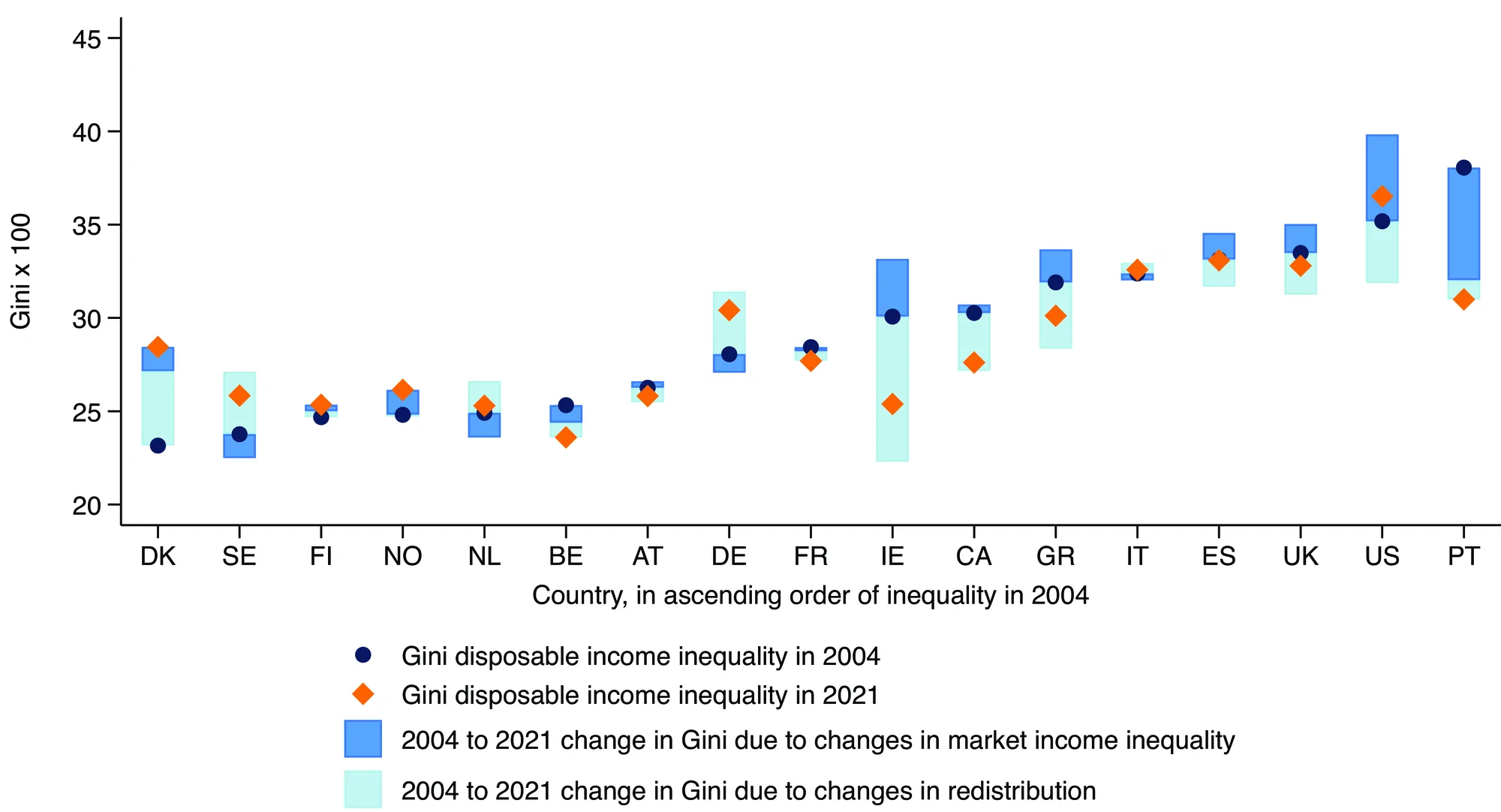
Decomposition of the change in disposable income inequality from 2004–2021 among the working-age population. Inequality is measured by the Gini coefficient times 100. The mid-blue color bar reflects the change in market income inequality from 2004–2021. Redistribution is the change in income inequality in moving from market income inequality to disposable income inequality as reflected in the light-blue color bar. See Eq. (**4**) in *Materials and Methods* for additional details. Income includes contributions from household members of all ages, and is equivalized using the Modified OECD scale. Working-age population is defined as those aged 25 to 60. Countries are listed in ascending order of initial 2004 disposable income inequality. For CA, income in 2004 is from 2012; for IT, income in 2021 is from 2022; and for PT, income in 2004 is from 2007.

Fig. 6 extends the analysis of cross-sectional levels of disposable income inequality to decomposing changes over time. Specifically, we wish to understand how much of the change in disposable income inequality over time is due to changes in market income inequality, and how much is due to changes in the effects of redistribution through the tax and transfer systems (See *Materials and Methods* for details). The figure displays the level of Gini disposable income inequality in 2004 (dark navy circle) and 2021 (orange diamond), where countries are listed in ascending order of initial year (2004) inequality. If the orange diamond lies above the dark navy circle, then that represents an increase in inequality in 2021 compared to 2004, and vice versa. The figure also depicts the change in Gini inequality attributable to changes in market-income inequality (mid blue bar) and the change in Gini inequality due to changes in redistribution (light blue bar). The area of the bars that lies above the 2004 inequality level indicates increases in inequality, whether from the disequalizing forces of rising market income inequality or from reductions in income redistribution. The area of the bars below the 2004 level indicates decreases in inequality, whether from equalizing forces of market incomes becoming more similar or expansions in the effects of redistributive tax and transfer programs. The initial year of 2004 is selected to coincide with maximal country data availability, and the *Robustness* section below discusses alternative time periods and inequality measures.

The results in Fig. 6 suggest that the modal country experienced increases in market income inequality from 2004–2021 with partial or complete offsetting from the effects of redistributive taxes and transfers. For example, the figure shows that inequality increased in the United States with rising market income inequality pulling disposable incomes apart, but with increases in the effects of redistributive taxes and transfers that substantially offset the rise in market income inequality. That is, in the absence of increased effects of redistribution, Gini inequality in the US would have been 0.40 in 2021 but instead it was 0.36. Ireland stands out with massive increases in redistribution more than offsetting increasing market-inequality forces, that therefore results in greater equality in disposable income. Canada, Greece, Spain, and the United Kingdom also experienced reductions in disposable income inequality by 2021 that are driven by higher redistribution more than offsetting rises in market income inequality. Belgium and Portugal are notable in that both greater equality of market incomes and rising redistribution resulted in lower inequality.

There are important exceptions to this pattern signified by retrenchment in the effects of redistribution from historically high redistributive countries. In the case of Denmark the growth in inequality came both from market incomes pulling apart and retrenchment in income redistribution that reinforced those market forces, while in Norway market incomes grew more unequal but the lack of response of income redistribution resulted in higher disposable income inequality (48, 64). Germany (DE), The Netherlands (NL), and Sweden (SE) are striking in that in the absence of the reduction in the effects of redistribution, disposable income inequality would have fallen in both countries by 2021.

### Robustness.

We adjust our income series for changing demographics prior to constructing our measures of inequality and redistribution (See *Materials and Methods*). The RIF adjustment implicitly assumes that the demographic controls are exogenous to inequality; that is, the demographics do not adjust in response to changes in inequality. This is most certainly true for our age and sex controls, but could be a possible concern with education attainment and household structure. While we acknowledge that inequality could affect *flows* into higher education or marriage/cohabitation, the *stock* of college graduates or married couples in the labor market will be less directly affected. To gauge whether the estimates are sensitive to this demographic adjustment, in *SI Appendix*, Figs. S5–S10 we replicate our analyses in the main text using raw data not adjusted for demographic composition. All of the key conclusions hold qualitatively when we do not adjust for demographic change, with one important difference: the unadjusted series reveals no divergence in market income inequality (cf. Fig. 2 and *SI Appendix*, Fig. S6). This is because some of the rising market income inequality across countries has been due to changes in the number of workers in the household, as also manifested in weaker divergence in household earnings inequality inclusive of nonworkers (cf. Fig. 3, panel 4 and *SI Appendix*, Fig. S7). There are also some interesting quantitative differences with the unadjusted series, including that average disposable income inequality increases faster over time (cf. Fig. 2 and *SI Appendix*, Fig. S6), there is less strong evidence of convergence in redistribution (cf. Fig. 5 and *SI Appendix*, Fig. S9), and there is a reordering of initial year inequality in the decomposition between Austria and Belgium, and Portugal and the United States, along with some differences in the relative size of changes in market income inequality and redistribution (cf. Fig. 6 and *SI Appendix*, Fig. S10). Using the RIF adjustment controls for those influences, allowing us to key in on structural and cyclical developments in labor markets and redistributive policies.

The Gini index is only one metric of inequality and it is well known to demonstrate more sensitivity to changes in the middle of the distribution and less sensitivity in the tails of the distribution (65). This potentially misses, for example, the increasing accumulation of income at the very top of the distribution in recent decades in some rich countries (10, 23, 37). *SI Appendix*, Figs. S11–S25 repeat the full set of figures contained in the main text (except Fig. 3 on labor markets), but using three alternatives that better capture changes in the tails of the income distribution. The first set in *SI Appendix*, Figs. S11–S15 is the ratio of average income in the top 5% of the distribution relative to the average income in the bottom 25% of the distribution (T5-B25 Ratio). This helps isolate changes in income accruing to the very rich relative to those for people toward the bottom of the distribution who rely more heavily on income from state transfers. Comparing the trends and patterns in Fig. 1 and *SI Appendix*, Fig. S11 we see little difference between the Gini coefficient and the T5-B25 Ratio, with the exception of a greater pulling away of US inequality where the top 5% average income grew from 8.5 times the bottom 25% to 11.5 times just prior to the COVID-19 crisis.

We do find larger estimates of the effects of redistribution with the T5-B25 Ratio (cf. Fig. 4 and *SI Appendix*, Fig. S13), but in general the decomposition of changes in disposable income inequality into that driven by market income inequality and redistribution components is unchanged (cf. Fig. 6 and *SI Appendix*, Fig. S15). Ireland (IE), Greece (GR), the UK, and US each had a very large increase in market income inequality that was largely offset by greater effects of redistribution from the top of the income distribution, while Belgium (BE) and Portugal (PT) had large decreases in market income inequality offset by a fall in redistribution from the top to the bottom. Overall, the qualitative and quantitative results from the Gini inequality index hold with the T5-B25 Ratio, though the statistical evidence of divergence in market incomes is slightly weaker when using the T5-B25 Ratio (cf. Fig. 2 and *SI Appendix*, Fig. S12).

We can obtain a more nuanced view of inequality changes by dividing the disposable income distribution into the top (top 5%–T5), the broad middle (the 70% between the 25th and 95th percentiles–M70), and the bottom (the bottom 25%–B25). We plot changes in the ratio of the mean income in the T5 to that in the M70 (*SI Appendix*, Figs. S16–S20) and the ratio of the mean income in the M70 to the B25 (*SI Appendix*, Figs. S21–S25). There is no evidence of convergence across countries in the T5-M70 ratio for disposable income or redistribution over time. Instead, the overall convergence apparent in Figs. 2 and 5 stems from convergence in the M70-B25 ratios. This suggests that cross-country convergence in disposable income inequality is occurring in the “lower 95%” of the income distribution. Likewise, we find no evidence of divergence in average market income inequality using the T5-M70 measure, but we do find such divergence in market income inequality for the M70-B25 measure (cf. Fig. 2 and *SI Appendix*, Figs. S17 and S22). There are also some interesting rearrangements across countries when examining upper and lower end inequality. The decomposition of changes in disposable income inequality from 2004–2021 suggests that the big increase in the effects of redistribution in Ireland, and the corresponding retraction in Germany and Sweden, are coming from the lower tail as reflected in M70-B25 (*SI Appendix*, Figs. S20 and S25). On the other hand, the reduction in the effects of redistribution in Denmark stems from changes at the top relative to the middle in the T5-M70 comparisons.

A related concern is treatment of incomes at the very top and bottom of the income distribution. As described in *Materials and Methods*, in order to remove the possible undue influence of extreme values we winsorize the income series for each country at the bottom 0.5% and top 99.5% of the distribution, meaning incomes below the 0.5% are set at the 0.5 percentile value and incomes above 99.5% are set at the 99.5th percentile value. *SI Appendix*, Figs. S26–S31 repeats the baseline Gini coefficient estimates and convergence tests based on an income series that winsorizes at the bottom 0.1% and top 99.9% of the distribution, thereby retaining more extreme high and low values of income. This has no qualitative or quantitative effect on our baseline results, with the exception of some slight reordering of countries in the decomposition of inequality change from 2004–2021 (cf. Fig. 6 and *SI Appendix*, Fig. S31).

We do not have a balanced panel of all countries spanning the entire sample period from 1995–2021. It is possible that some of our convergence tests could be sensitive to the fact that some countries move in and out of the sample over time. In *SI Appendix*, Figs. S32–S37 we repeat the analysis using the balanced subset of eight countries that have data in every year from 1995–2021 (Denmark, Finland, Germany, The Netherlands, Norway, Sweden, United Kingdom, and United States). Quantitatively we find: average disposable income inequalities increase faster over time compared to the base sample; average market income inequalities increase more slowly (cf. Fig. 2, *Left* panel and *SI Appendix*, Fig. S33); the 8 countries converged in disposable incomes slower over time; but they diverged in market incomes more quickly (cf. Fig. 2, *Right* panel and *SI Appendix*, Fig. S34). This implies the convergence in the effects of redistribution in the balanced sample of eight countries was accelerated (cf. Fig. 5 and *SI Appendix*, Fig. S36). However, there is no qualitative change in our central findings of convergence in both disposable income inequality and redistribution, and divergence in market income inequality. Moreover, the longer time horizon further underscores the important role that the retreat from redistribution among highly redistributive states (the Nordics plus Germany and The Netherlands) played in the evolution of income inequality over the past three decades (cf. Fig. 6 and *SI Appendix*, Fig. S37).

Another possible concern over the choice of sample period is that the last 2 y span the COVID-19 pandemic when extraordinary measures were taken in some countries to alleviate the economic contraction. This was especially true in the US where there was an infusion of $5 trillion in safety net spending, and with smaller but still substantive government interventions in Canada and the UK. To test whether our findings are robust to the sample period, in *SI Appendix*, Figs. S38–S43 we present estimates spanning 1995–2019, the year prior to the pandemic. If anything the tests of convergence in disposable income inequality and redistribution, and divergence in market inequality are stronger when ending the sample in 2019. There are some expected differences in the inequality change decomposition for 2004–2019 (cf. Fig. 6 and *SI Appendix*, Fig. S43). Notably the effects of redistribution are attenuated in Canada, the UK, and the US. In the case of Canada this means there is less of a reduction in inequality from 2004–2019 than in 2021, while in the US this has the effect of exacerbating the increase in inequality. In the UK there is little change in inequality as in Fig. 6, but in this case because there is little change in either market inequality or the effects of redistribution.

In computing cross-country averages and dispersion in Figs. 2, 3, 5, and 6 we do not weight by the country population (though the microdata underlying the country inequality series are weighted using the individual sample weights where appropriate). The reason for using unweighted country averages is that we want to understand the role of cross-country differences in labor markets and institutions in explaining income inequality–specifically redistribution through the tax and benefit system–and thus do not want to give too much weight to a large country like the US or too little weight to a small country like Ireland. However, to verify that our convergence results are not unduly affected by any one country, in *SI Appendix*, Table S1 we report the coefficient and SE on the trend coefficient from a series of “leave one country out” regressions of dispersion in disposable income inequality, market income inequality, and the effects of redistribution (Gamma) akin to those reported in Figs. 2 and 5. That is, we omit the country indicated in each of the 17 rows and recompute our measures of dispersion on the remaining 16 countries, and then regress that new measure on the linear trend and constant term. The table shows that our central results of convergence in disposable income inequality and redistribution, and divergence in market income inequality, are uniformly robust to omission of any one country from the analysis.

## Discussion

We focus on the evolution of disposable income inequality across high-income European and North American economies over the past three decades to assess whether the countries are becoming more similar over time in their inequalities. In particular, our aim is to understand whether disposable income inequality has converged, and if so, whether that convergence stems from market income inequality becoming more similar across countries, or whether the impact of country-specific systems of governmental tax and transfer programs designed to reduce inequality has become more similar over time. To address these questions, we assemble an innovative panel of countries over time drawing from administrative, survey, and hybrid administrative-survey data sources.

Our results convey a clear and consistent story that overall disposable income inequality has converged since the mid 1990s, and this convergence is associated with redistribution converging and not market income inequality, which has diverged over time. While disposable income inequality has converged toward a higher level today than 30 years ago, the experience across the 17 countries is quite heterogeneous with inequality increasing among historically low inequality Nordic countries like Denmark, Sweden, and Norway, and inequality declining among the historically high inequality countries of Ireland and Portugal. The United States remains somewhat of an outlier with high and rising inequality over the whole period (66), whereas a plurality of other countries experienced very little change in inequality. For those economies, market income inequality was stable, as was the level of redistribution, resulting in disposable income inequality roughly on par with that experienced at the start of the sample period. In *SI Appendix*, section 1, we use results from the individual country studies in our associated project (51) to place our sample period in longer term context. Our 1995–2021 period has generally smaller movements in disposable income inequality than the preceding 15 y and market income inequality played a bigger role in the earlier period while, as we show here, changes in the effects of the redistributive system are bigger contributors to cross-country patterns in our period.

The countries in our analysis share the common feature of democratically elected governments with decades, and in some cases, centuries, of experience developing and reforming social policies that aim for a more equitable distribution of income. These countries continue to function as “laboratories of democracy” to design tax and transfer programs suitable to their country’s population and political economy environment, and yet despite underlying differences in labor markets, the levels of income redistribution have become more similar over time. Notwithstanding growing similarities in redistribution, countries do often choose to provide different levels of assistance in response to economic shocks. This was underscored in the recent COVID-19 pandemic where Canada and the United States were notable in that they had significant increases in income redistribution in response to the pandemic, suggesting a difference in North American responses to the crisis compared to Continental Europe which relied more on job-retention, wage-subsidy, and furlough-type schemes rather than cash transfers (67).

Even though in any given year, tax and transfer systems are effective in mitigating inequality, the policies have not reversed the trend increase in market income inequality across the 17 countries. This could be a result of political economy within countries, with voters demanding a safety net response to economic shocks—whether idiosyncratic to the household or economy wide—but not a sustained commitment sufficient enough to roll back the rising tide of inequality, especially at the upper tail. There are notable exceptions in Ireland and the Nordic countries. In Ireland, during and in the immediate aftermath of the Great Recession, which had a devastating effect on the Irish economy, redistribution increased substantially, predominantly as a result of the tax and benefit system acting as an “automatic stabilizer,” helped by additional progressive policy changes (68, 69). The Nordic countries, on the other hand, gradually rolled back their redistributive tax and transfer programs through policy reform, and this has resulted in growing disposable income inequality (64, 70). This unwinding of redistribution also occurred in Germany and The Netherlands.

The finding of divergence in market income inequalities in these advanced economies, notably at the level of household earnings, suggests that policies affecting pretax inequality such as education, labor market policies such as minimum wages, or other factors such as technological change, globalization, female labor force participation, and their interactions with marriage and cohabitation patterns, are driving changes in labor-market opportunities across countries. What we have demonstrated in this paper is that these forces have been offset by a greater similarity across countries in the effects of redistributive tax and transfer policies, albeit the convergence has been toward a lower degree of redistribution.

## Materials and Methods

### Data Sources.

The analysis is based on microdata from 17 European and North American countries obtained from administrative tax register data, survey data, and hybrid survey and register data. The countries using register data include Denmark (BEF, IND, UDDA, 1995–2021), The Netherlands (Income Panel, 1995–2011; INHATAB 2011–2021), Norway (Aa-registeret, LTO, Skattemeldingsregisteret, NUDB, 1995–2021), and Sweden (LISA, 1995–2021). The countries using survey-only data are Austria (EU-SILC, 2004–2021), Belgium (European Community Household Panel, 1996–1997, 1999–2001; Socio-Economic Panel, 1997; EU-SILC, 2004–2021), Germany (Socio-Economic Panel, 1995–2021), Greece (EU-SILC, 2004–2021), Ireland (Living in Ireland Survey, 1995–2001; EU-SILC, 2004–2021), Italy (Survey of Households Income and Wealth, 1995; biennially 1998–2016; 2020; EU-SILC 2006–2021), Portugal (EU-SILC, 2006–2021); Spain (EU-SILC, 2004–2021), and the United States (Current Population Survey Annual Social and Economic Supplement, 1995–2021). The countries using a hybrid of administrative and survey data are Canada (Labour Force Survey 1997–2021; Canadian Income Survey, 2012–2021), Finland (Income Distribution Survey, Structure of Earnings Survey, 1995–2021), France (Fiscal Survey linked to administrative income data, 1996–2021), and the United Kingdom (Family Resources Survey with top incomes adjusted using tax records, 1995–2021).

The data for all measures of income inequalities used in this paper and the supplemental appendix are available for readers to download at https://ifs.org.uk/journals/unpacking-transatlantic-income-inequality. At this same address, replication files are available, which consist of a set of Stata .do files. These can be run on the data stored there, allowing readers to reproduce all the results in this paper.

### Data Harmonization.

We harmonize the variable definitions and data processing to ensure consistency across countries. Market income includes labor-market earnings from employers and self-employment, private pensions, financial transfers from relatives and friends, and ordinary capital income from rent, interest, and dividends. Disposable income adds to market income government cash transfers and nonhealth near-cash transfers such as food and housing benefits, and subtracts away income tax payments inclusive of any refundable tax credits. We do not include employer social security, pension, or health insurance contributions toward income. In Swedish data, ordinary capital income is not separately identifiable from capital gains, and so is excluded.

The household is defined as all persons coresiding at the same residence, and financial contributions of all household members regardless of age are included in market and disposable income. Incomes are measured at the annual level, equivalized using the Modified OECD scale, and placed in real, inflation-adjusted terms using the 2019 Consumer Price Index in each country. We then select our analysis sample as persons aged 25 to 60, meaning we use the equivalized household incomes for each sample member, and thus all adults in the same household who meet the age criteria will appear in the individual data with the same level of equivalized incomes. Winsorizing occurs at the 0.5th and 99.5th percentile of the positive distribution for all income variables to minimize the undue influence of extreme values; that is, for households with incomes below the 0.5th percentile they are assigned the 0.5th value, and for those households with incomes above the 99.5th percentile they are assigned the 99.5th value. We then add back to the analysis sample those individuals aged 25 to 60 residing in households reporting zero market or disposable incomes.

We also harmonized our labor-market variables of hourly wages, individual earnings, and equivalized household earnings. Wages are measured as the ratio of annual labor-market earnings to annual hours of labor-market work only for those employed by others and excluding the self-employed. Annual earnings are measured for all workers, both individuals employed by others and the self-employed. Household earnings of workers are measured by adding up annual earnings of all persons working for others or self; and household earnings of all individuals add to the latter sample of workers those households reporting zero earnings from the labor market. In the UK, these variables are measured at the “pay period” level, generally per month or week. For the household measures we include the earnings of all household members regardless of age, and then equivalize using the Modified OECD scale and select our sample of individuals from the working-age population aged 25 to 60. We use the relevant individual sample weight to construct each country’s time series of equivalized inequality measures of wages, earnings, and incomes.

### Controlling for Demographic Compositional Change.

Given the substantial demographic changes across countries over the past three decades—including shifts in age structure, education levels, and household composition—we control for these compositional effects using the Recentered Influence Function (RIF) regression approach (57). This allows us to examine how inequality metrics would have evolved if demographic characteristics had remained fixed at a base year, which we set at 2015; that is, it enables us to separate the effects of structural economic change such as technology shocks or globalization from those driven by shifts in population composition or labor market selection. This is implemented as follows. In the first step we regress our RIF of interest, $RIF(y_{i,j.t},\upsilon_{j,t}\left(F_{Y}\right))$, on a constant term and set of demographic controls $\left(x_{i,j,t}^{k}\right)$:

[1] $$RIF\left(y_{i,j,t},\upsilon_{j,t}\left(F_{Y}\right)\right)=\beta_{j,t}^{0}+\sum_{k=1}^{K}\beta_{j,t}^{k}x_{i,j,t}^{k}+\varepsilon_{i,j,t},$$

where $y_{i,j,t}$ is market or disposable income of individual *i* in country *j* in year *t*, $\upsilon_{j,t}\left(F_{Y}\right)$ is our functional statistic of interest such as the Gini inequality index $I_{j,t}^{\mathrm{Gini}}$, and ε is a mean-zero error term. Our demographic controls include single-year age dummies (age 30 omitted); number of adults age ≧ 19; number of children age ≦ 18; and indicators for presence of child age <5, sex (=1 if female), three education categories based on the International Standard Classification of Education (ISCED 0 to 2, 3 to 5, 6 to 8, with ISCED 3 to 5 omitted), and interactions between sex and education. Education in Finland is based on ISCED 0 to 2, 3 to 6, 7 to 8, while The Netherlands has no information on education linked to income. Note that while we lack sufficient data across countries over time explicitly identifying immigrant status to include as a separate control variable, the influence of immigrants will be partly reflected in changes in the age, education, and household composition of countries.

Because the expected value of the left-hand-side of Eq. (**1**) is the functional statistic, $\upsilon_{j,t}\left(F_{Y}\right)$, then with the estimated coefficients, $\hat{\beta }$, we form a counterfactual Gini by predicting what Gini inequality would be at the country level *j* in a given period *t* if the same set of demographic controls were fixed at their 2015 mean values in country *j* $\left({\bar{x}}_{j,2015}^{k}\right)$:

[2] $${\hat{I}}_{j,t}^{\mathrm{Gini}}={\hat{\beta }}_{j,t}^{0}+\sum_{k=1}^{K}{\hat{\beta }}_{j,t}^{k}{\bar{x}}_{j,2015}^{k}.$$

This approach is followed in constructing the Gini series for both market income inequality and disposable income inequality. Indeed, it can be applied to any quantile of the income distribution, and as such we estimate each centile of the distribution to form the counterfactual at each centile anchored to 2015 mean values of demographics. This is then used in constructing the T5-B25 Ratio, as well as the T5-M70 and M70-B25 Ratios in the *SI Appendix*.

### Measuring Convergence in Inequality.

We formally test whether inequality and redistribution across countries has converged using the metric of *sigma*-convergence, which focuses on whether the dispersion of inequality (or redistribution) across countries narrows over time (36, 38–40, 45, 52, 63, 65–69). Our measure of dispersion is the normalized coefficient of variation (*NCV*), defined as $NCV=\frac{\mathrm{CV}}{\left(1+CV\right)}$, where CV=σ/μ is the coefficient of variation with σ the SD and μ the mean. The *NCV* is unit free and bounded between 0 and 1 such that as it approaches 0 dispersion falls and as it approaches 1 dispersion increases. For ease of presentation in the figures, we multiply the Gini index and NCV by 100. As discussed in the Robustness section, all estimates of cross-country averages and dispersion are not weighted by country population in order to give equal weight to each country’s labor market and institutions.

### Measuring Redistribution.

To measure the role of redistribution in shaping inequality, we use a slightly modified version of the Reynolds-Smolensky index, the latter of which compares the level difference of market income inequality and disposable income inequality (71). Our version places it in terms of percentage change (47). Specifically, in the context of the Gini coefficient from the counterfactual RIF regression framework in Eq. (**2**) this is estimated for country *j* in time *t* as

[3] $${\hat{\gamma }}_{j,t}^{\mathrm{Gini}}=100*\left(1-\frac{{\hat{I}}_{j,t}^{\mathrm{DispGini}}}{{\hat{I}}_{j,t}^{\mathrm{MarketGini}}}\right),$$

where ${\hat{I}}_{j,t}^{\mathrm{DispGini}}$ and ${\hat{I}}_{j,t}^{\mathrm{MarketGini}}$ are the predicted Gini coefficients for disposable and market income, respectively. Higher values of gamma (${\hat{\gamma }}_{j,t}^{\mathrm{Gini}}$) imply more redistribution that occurs through the tax and transfer system. Estimates of gamma are also obtained using the T5-B25, T5-M70, and M70-B25 Ratios presented in the *SI Appendix*.

In addition, we decompose the evolution of disposable income inequality over time into changes in market income inequality and changes in redistribution through taxes and transfers. In Fig. 6 this is done between the years 2004 and 2021 for the Gini coefficient as

[4] $$\begin{array}{c} {\hat{I}}_{j,2021}^{\mathrm{DispGini}}-{\hat{I}}_{j,2004}^{\mathrm{DispGini}}=\left({\hat{I}}_{j,2021}^{\mathrm{MarketGini}}-{\hat{I}}_{j,2004}^{\mathrm{MarketGini}}\right)\\ +\left\{\left({\hat{I}}_{j,2021}^{\mathrm{DispGini}}-{\hat{I}}_{j,2021}^{\mathrm{MarketGini}}\right)-\left({\hat{I}}_{j,2004}^{\mathrm{DispGini}}-{\hat{I}}_{j,2004}^{\mathrm{MarketGini}}\right)\right\}, \end{array}$$

where the left-hand-side is the change in disposable income inequality, the first term on the right-hand-side reflects changes in market income inequality (the mid blue color bar in Fig. 6), and the second term between curly brackets reflects changes in redistribution (the light blue color bar in Fig. 6). This decomposition can be conducted across any pair of years, and for other measures of inequality such as found in the *SI Appendix*.

## Supplementary Material

Appendix 01 (PDF)

## Data Availability

The data for all measures of income inequalities used in this paper and the supplemental appendix are available for readers to download at https://ifs.org.uk/journals/unpacking-transatlantic-income-inequality (72). At this same address, replication files are available, which consist of a set of Stata .do files. These can be run on the data stored there, allowing readers to reproduce all the results in this paper.
