# Supplementary material for: Unpacking transatlantic income inequality
Source: Proc Natl Acad Sci U S A. 2026 Nov 3;123(45):e2625474123. doi: 10.1073/pnas.2625474123 (PMC13751648; doi:10.1073/pnas.2625474123)
Supplement: Supplementary file 1 [file pnas.2625474123.sapp.pdf]

PNAS

# Supporting Information for
## Unpacking Trans-Atlantic Income Inequality

James P. Ziliak, Jonathan Cribb, Richard Blundell, James Banks, David Green, Antoine Bozio, Malka Guillot, Barra Roantree, Maxime To, Brindusa Anghel, Cristina Barceló, Jim Been, Patrick Bennett, Maximilian Blömer, Olympia Bover, Koen Caminada, Bart Capéau, Daniele Checchi, Svetoslav Danchev, André Decoster, Bram De Rock, Kelly Foley, Giorgos Gatopoulos, Martin Halla, Bradley Hardy, Elena Herold, Laura Hospido, Tullio Jappelli, Egbert Jongen, Niki Kalavrezou, Arizo Karimi, Elizabeth Krause, Max Lay, Soren Leth-Petersen, Charlotte Lucke, Immacolata Marino, Antonis Mavropoulos, Carlos Oliveira, Mårten Palme, Tuuli Paukkeri, Grigoris Pavlou, Andreas Peichl, Pedro Portugal, Lukas Puschnig, Pedro Raposo, Ann-Christin Rathje, Terhi Ravaska, Craig Riddell, Marja Riihelä, Johan Saeverud, Kjell G. Salvanes, Annalisa Scognamiglio, Anne Steuernagel, Jonas Vanderkelen, Heike Vethaak, Nikos Vettas, Andrea Weber, Thomas Wernham

Richard Blundell
Email: r.blundell@ucl.ac.uk

**This PDF file includes:**

- Supporting text
- Figures S1 to S43
- Tables S1 to S2
- Additional References

**Supporting Information Text**

**1. Market Income Inequality**

Fig. S1 presents trends in market income inequality for each country and year using the Gini coefficient. Compared to disposable income inequality, inequality in market incomes is higher in each year for all countries, with notable changes in Ireland and the Nordic countries where there is much more variation (cf. Fig. 1; Fig. S1).

It is useful to place the movements in our sample period (1995 to 2021) in the context of longer-term trends. The surge in inequality (both in market and disposable income) in the US and UK in the 1980s is perhaps the best-known element of the longer-term international income inequality trends (1,2). After 1990, inequality has continued to grow in the US, but at a slower rate than in the headline decade of the 1980s (3). An advantage of our data is that we are able to see, using harmonized data, whether the pattern in the UK and US of slower inequality growth from the mid-1990s onwards is a common pattern. Consistent microdata are not available for many countries (data availability dictates our sample period in this paper). However, for a significant number of countries we are able to see longer term trends. Those trends are presented in a collection of papers published in *Fiscal Studies* in 2024, each discussing the trends and composition of inequality in one of the countries in this project (4).

Working with the data in the individual countries, we find that the pattern of substantial increases in inequality in the 1980s and early 1990s followed by either no or slower growth beginning in the mid-1990s is present in many of our countries (the UK, Canada, Finland, Italy, and the Netherlands). Within this group, the UK, Canada, and Finland adopt essentially flat inequality trends in our sample period while the US, Italy, and to some extent, the Netherlands have further (though smaller) disposable income inequality increases after 1995 relative to before. Ireland, Norway and France also see much bigger changes in inequality prior to our sample period but in their cases, the prior changes are declines. For Ireland and Norway, the declines occurred in the 10 to 15 years prior to our sample period while for France they occurred much earlier, in the 1970s.

Thus, a general conclusion is that our sample period is one of more temperate changes in disposable income inequality (up or down) than the preceding period. To some degree, also, the changes in the 1980 to 1995 period were driven more by changes in market income inequality while changes in the effects of the redistributive system make up more of the story after 1995, as our analysis shows. The main exceptions to the general pattern of our sample period being quieter for inequality movements are Germany, Denmark and Sweden, all of which experienced their larger inequality increases in our sample period than in the years preceding the mid 1990s.

**2. Labor-Market Inequalities**

We explore whether the underlying source of divergence in market income inequality in the main text emanates from the labor market. Specifically we examine hourly wages of employees, defined as average hourly earnings for those employed by others and excluding the self employed; individual earnings of workers, defined as annual earnings of individuals employed by others or self; household earnings of workers, defined by adding up annual earnings of all persons working for others or self; and household earnings of all individuals, which includes non-working households meaning there are those in this group with zero earnings at the household level. For the household measures we include the incomes of all household members regardless of age, and then equivalize using the Modified OECD scale and select our sample of individuals from the working-age population aged 25-60.

Fig. S2 depicts the average level of Gini inequality times 100 across the 17 countries for each of these four labor-market outcomes, along with a linear trend line and its associated coefficient and standard error. The figure makes clear that inequality is trending upward over our sample period

for wages, earnings, and household earnings. This rate of increase is most rapid for household earnings of workers, suggesting that differences in the number of workers and labor-market effort (i.e. part-time/full-time) across countries is driving some of the growth in labor-market inequality. Fig S3 underscores this latter point by presenting the share of households in each country over time that do not have any workers, i.e. they report zero earnings. Among English-speaking countries, Ireland and the UK stand out with elevated rates of workless households, with a strong surge during the Great Recession for Ireland. Belgium is notable for high rates of workless households among the Central European countries, while Greece has above-average rates among Southern European countries. Lastly, the main text focuses on convergence in labor-market inequality measured by the Gini index. Fig. S4 focuses on upper-tail and lower-tail inequality by depicting the ratio of the $90^{th}$ to $50^{th}$ percentiles and the $50^{th}$ to $10^{th}$ percentiles, respectively. The figure shows that convergence in wage inequality across the 17 countries is because of convergence at the lower tail of the wage distribution, while divergence in earnings is occurring in both the upper and lower tails of the respective distributions.

## 3. Inequality not Adjusted for Demographic Composition

In the main text we adjust all of our income levels and inequality measures using Recentered Influence Function (RIF) estimators anchored to the 2015 calendar year. In Fig. S5-S10 we replicate our analyses in the main text using raw data not adjusted for changing demographic composition. All of the results hold qualitatively when we do not adjust for demographic change, with the notable exception that we find no evidence of divergence in market incomes (Fig. S6), which operates through workless households (Fig. S7). This suggests that some of the divergence in inequality in labor-market outcomes is operating through shifting demographics, underscoring the importance of conditioning on those changes with the RIF methodology.

The remaining figures in this appendix (Figs. S1-S4 and S11-S43) use demographically-adjusted data series with the RIF method described in the *Materials and Methods* of the main text.

## 4. Alternative Measures of Inequality

The main text Fig. 1-6 are all based on the Gini coefficient. In this section we test the sensitivity of our findings to choice of inequality measure.

The first alternative measure is reported in Subsection A Fig. S11-S15, where we repeat the analysis of the main text with a measure of inequality that may better capture changes at the top of the income distribution relative to the bottom. Specifically, we compute the ratio of the average income for the top 5% of the distribution relative to the average income in the bottom 25% (T5-B25 Ratio). Overall, the qualitative and quantitative results from the Gini inequality index hold with the T5-B25 Ratio, though the statistical evidence of divergence in market incomes is a little weaker when using the T5-B25 Ratio (cf. Fig. 2; Fig. S12). We do find a greater pulling away of US inequality from the other 16 countries where the top 5% average income grew from 8.5 times the bottom 25% to 11.5 times just prior to the Covid-19 crisis. We also find larger estimates of redistribution with the T5-B25 Ratio (cf. Fig. 4; Fig. S13), but with the exception of The Netherlands (NL) and Norway (NO) switching places along with Italy (IT) and Spain (ES), the ordering of the decomposition into market income inequality and redistribution components is unchanged (cf. Fig. 6; Fig. S15).

In Subsections B and C corresponding to Fig. S16-S25 we examine whether the patterns we document in the T5-B25 measure emanate from changes in the upper-tail of the distribution by comparing the top 5% average income relative to the average income of the middle 70% group just below (T5-M70 Ratio), or from changes in the lower-tail of the distribution by comparing the average income of the “middle 70%” to the average income in the bottom 25% (M70-B25 Ratio). The results suggest that convergence in Gini disposable income inequality in the main text is being driven by changes at the bottom 95% of the distribution as we find no evidence of convergence in disposable income or redistribution between the top 5% and middle 70% (cf. Fig.

2 and 5 to Fig. S17 and S19), whereas we find strong evidence of convergence in these outcomes when comparing the middle 70% to bottom 25% (cf. Fig. 2 and 5 to Fig. S22 and S24). Likewise, we find no evidence of divergence in market incomes for the T5-M70, but we do find such divergence in market incomes for the M70-B25 measure (cf. Fig. 2 to Fig. S17 and S22). The decomposition of changes in disposable income inequality from 2004 to 2021 suggests that the expansion in redistribution in Ireland, and the corresponding retraction in Germany and Sweden, are coming from the lower tail (Fig. S20 and S25). On the other hand, such retraction in redistribution in Denmark stems from changes at the top relative to the middle.

### 5. Alternative Methods of Data Censoring

In the main text we winsorize the income series at the 0.5 and 99.5 percentiles. In Fig. S26-S31 we repeat the analysis when we winsorize at the 0.1 and 99.9 percentiles, which retains more extreme high- and low-income values in the sample. This has no qualitative or quantitative effect on our baseline results, with the exception of weaker statistical evidence of divergence in market incomes (cf. Fig. 2; Fig. S27) and some reordering of countries in the decomposition of inequality change from 2004-2021 (cf. Fig. 6; Fig. S31).

An issue that has been shown to affect major household surveys is the underreporting of income transfers (5-6), including some of those used in this study. Recent evidence in the context of the US utilizing regression-based imputation methods suggests that transfer underreporting has some effect on the level of absolute poverty rates, but no effect on poverty trends (7). The attenuated effect on inequality levels compared to poverty rates likely stems from the fact that some social insurance programs such as unemployment and disability reach much higher in the income distribution, thus dispersing the potential impact across more of the distribution. While making such adjustments across all the countries using survey-based data is beyond the scope of this project, based on the US evidence we do not believe our convergence tests of inequality are likely affected.

### 6. Balanced Panel of Countries

We have an unbalanced sample of countries over the 1995-2021 sample period. Thus, to test whether our key conclusions are robust to imposing a balanced sample, in Fig. S32-S37 we repeat the analysis using the 8 countries for whom we have complete data for all sample years: Denmark, Finland, Germany, The Netherlands, Norway, Sweden, United Kingdom, and United States. Quantitatively we find that average disposable income inequalities increase faster over time compared to the base sample, and average market income inequalities more slowly, the 8 countries converged in disposable incomes slower over time, but diverged in market incomes more quickly (cf. Fig. 2; Fig. S33). This implies the convergence in redistribution in the balanced sample of 8 countries was accelerated (cf. Fig. 5; Fig. S36). However, there is no qualitative change in our central findings of convergence in disposable income inequality emanating from convergence in redistribution and not market incomes when looking at the balanced panel over a longer time horizon.

### 7. Pre-COVID-19 Sample Period

The baseline sample used in our main text ends in 2021, which means the last two years of the sample overlap with the onset and early recovery from the COVID-19 pandemic. Because several countries substantially expanded redistribution through social insurance and means-tested transfers to support households through the pandemic, it is possible that some of our tests of convergence of disposable income and redistribution could be affected by extraordinary schemes. To test whether our findings are robust to the sample period, in Fig. S38-S43 we present estimates spanning 1995 to 2019, the year prior to the pandemic. Overall the tests of convergence in disposable income inequality and redistribution, and divergence in market income inequality, are stronger when ending the sample in 2019. There are some expected differences in the inequality change decomposition for 2004-2019 (cf. Fig. 6; Fig. S43). Notably the effects of

redistribution are attenuated in Canada, the UK, and the US. In the case of Canada this means there is less of a reduction in inequality from 2004 to 2019 than in 2021, while in the US this has the effect of exacerbating the increase in inequality. In the UK there is little change in inequality (as in Fig. 6), but in this case because there is little change in either market inequality or the effects of redistribution, rather than offsetting effects.

**8. Leave One Country Out**

In order to verify that our convergence results are not unduly affected by any one country, in Table S1 we report the coefficient and standard error on the trend coefficient from a series of "leave one country out" regressions of dispersion in disposable income inequality, market income inequality, and the effects of redistribution (Gamma). To operationalize the test, we omit the country indicated in each of the 17 rows and recompute our measures of dispersion on the remaining 16 countries. We then regress that new measure on the linear trend and constant term. The table shows that our central results of convergence in disposable income inequality and redistribution, and divergence in market income inequality, are uniformly robust to omission of any one country from the analysis.

**9. Beta Convergence**

Our tests of convergence are based on the notion of *sigma*-convergence whereby we test whether the dispersion in inequality has declined across countries over time. An alternative approach, and the workhorse method employed in scores of cross-country growth papers on income levels (8-13), is known as *beta*-convergence. In our case the test of *beta*-convergence is whether the level of inequality or redistribution, not the dispersion, has converged across countries. Notably, *beta*-convergence is necessary, but not sufficient, for *sigma*-convergence, and this is an additional justification for our emphasis on tests of *sigma*-convergence (dispersion) in the main text (14-15).

However, in Table S2 we report the results of tests of *beta*-convergence in disposable income inequality, market income inequality, and Gamma redistribution. The table reports estimates of the coefficient $\beta$ and its standard error from the following cross-country panel-data regressions:

(S1) $\Delta lny_{jt} = \alpha + \beta lny_{jt-1} + u_{jt}$,

where $\Delta lny_{jt}$ is the change from year $(t-1)$ to $t$ in the natural log of inequality or redistribution in country $j$, $lny_{jt-1}$ is the lagged level of the dependent variable, $\alpha$ is a regression constant term, and $u_{jt}$ is a model error term.

The test of *beta*-convergence is whether the estimated coefficient $\hat{\beta}$ is negative and statistically significantly different from 0. For each of the three dependent variables we perform two tests, one of "absolute" *beta*-convergence, which involves estimating equation (S1) as a pooled cross-section time series regression (pooled OLS regression), and the second test of "conditional" *beta*-convergence, which involves adding indicator variables for each country to the regression (country fixed effects regression). Absolute beta-convergence occurs when countries share a set of common "deep parameters", e.g. discount factors, capital share, population growth, and converge to a common steady state level of inequality or redistribution, while the conditional beta-convergence occurs only after controlling for those fixed country-specific factors and thus countries converge to their own steady state (8).

Table S2 shows that there is evidence of both absolute and conditional *beta*-convergence in levels of both market and disposable income inequality, as well as in (Gamma) redistribution. Note that in Fig. 2 we find divergence in the dispersion of market-income inequality, whereas in Table S2 we find evidence of convergence in the levels of market-income inequality. This

underscores the importance of our tests of *sigma*-convergence in the main text as we confirm here that *beta*-convergence is not sufficient for sigma-convergence.

## Figures

### 1. Market Income Inequality

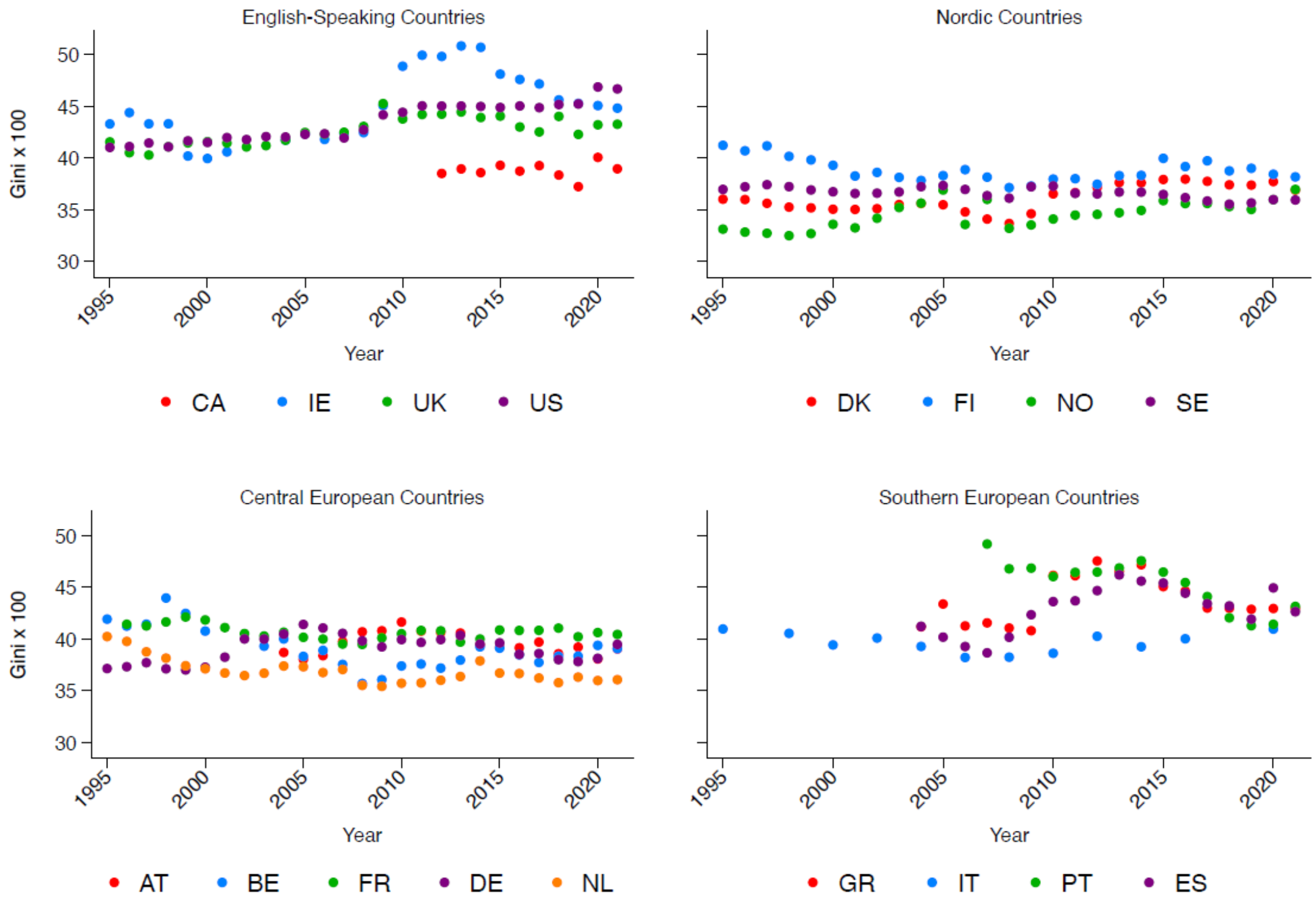


**Fig. S1.** Cross-Country Trends in Market Income Inequality among the Working-Age Population. Inequality is measured by the Gini coefficient. Market income is the sum of labor earnings, ordinary capital income, and nongovernmental transfers. Income includes contributions from household members of all ages, and is equivalized using the Modified OECD scale. Working age population is defined as those aged 25-60. Countries are grouped into English-speaking nations of Canada (CA), Ireland (IE), United Kingdom (UK), and United States (US); Nordic countries of Denmark (DK), Finland (FI), Norway (NO), and Sweden (SE); Central European countries of Austria (AT), Belgium (BE), France (FR), Germany (DE), and The Netherlands (NL); and Southern European nations of Greece (GR), Italy (IT), Portugal (PT), and Spain (ES).

## 2. Labor-Market Inequalities

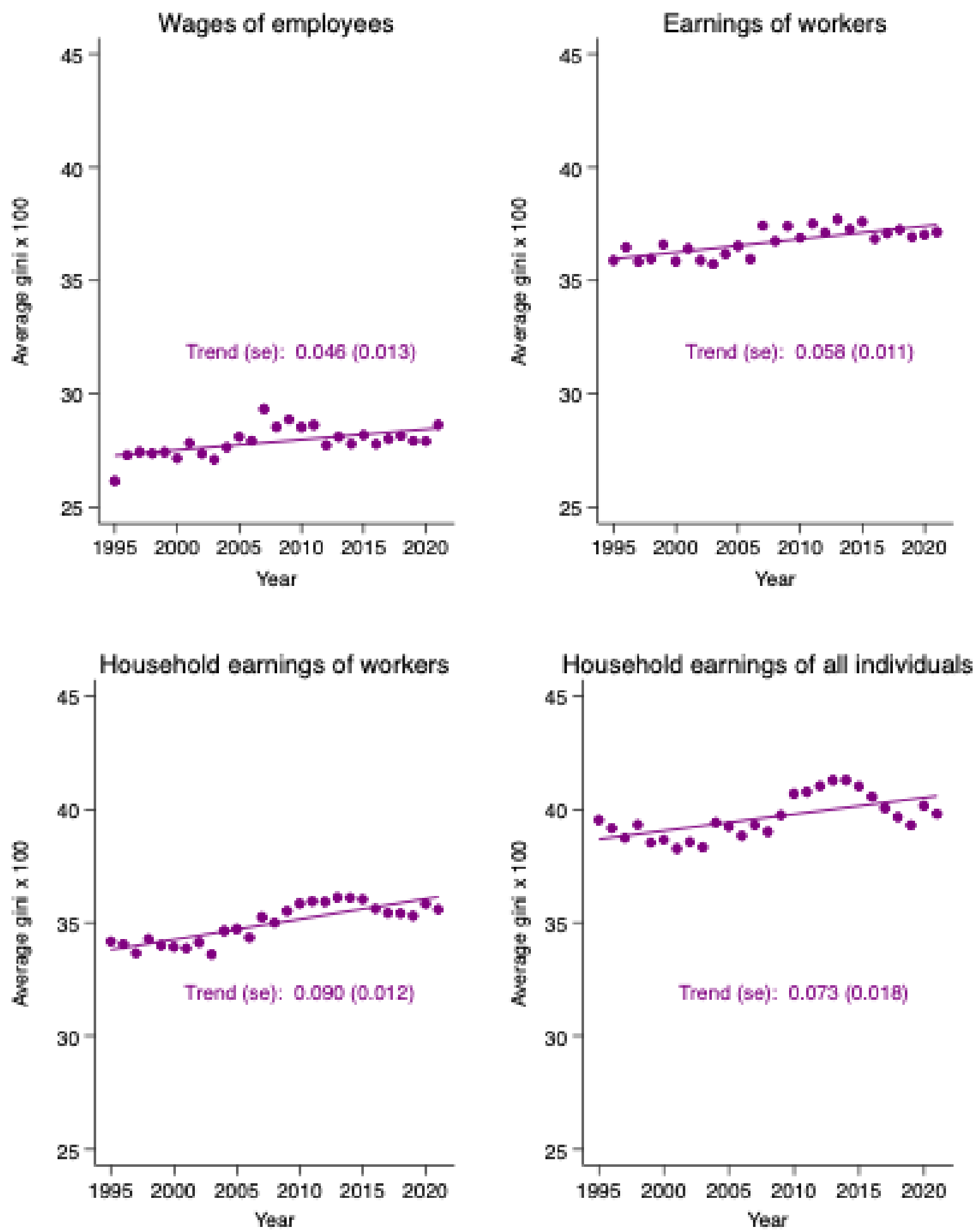


**Fig. S2.** Trends in Average Labor-Market Inequalities among the Working-Age Population. Inequality is measured by the Gini coefficient times 100. Wages of employees are average hourly earnings for employees. Earnings of workers are annual earnings of employees and self-employed workers. Household earnings of workers add up annual earnings of all persons who are employees or self-employed workers**.** Household earnings of all individuals include non-working households. Household earnings are equivalized using the Modified OECD scale. Working-age population is defined as those aged 25-60.

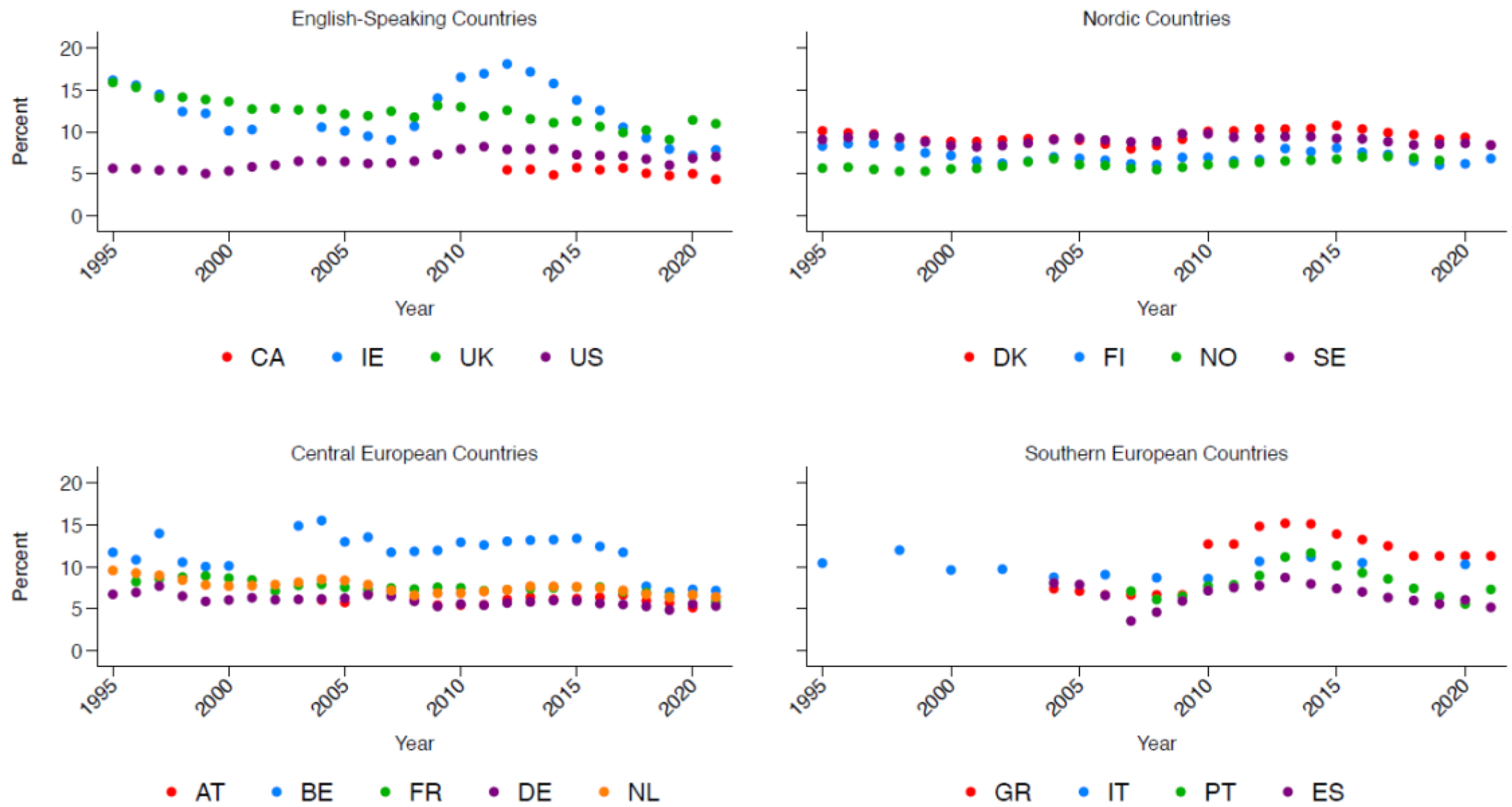


**Fig. S3.** Cross-Country Trends in the Share of Workless Households among the Working-Age Population. The figure depicts the percentage of households in each year that report no labor-market earnings. Working-age population is defined as those aged 25-60. Countries are grouped into English-speaking nations of Canada (CA), Ireland (IE), United Kingdom (UK), and United States (US); Nordic countries of Denmark (DK), Finland (FI), Norway (NO), and Sweden (SE); Central European countries of Austria (AT), Belgium (BE), France (FR), Germany (DE), and The Netherlands (NL); and Southern European nations of Greece (GR), Italy (IT), Portugal (PT), and Spain (ES).

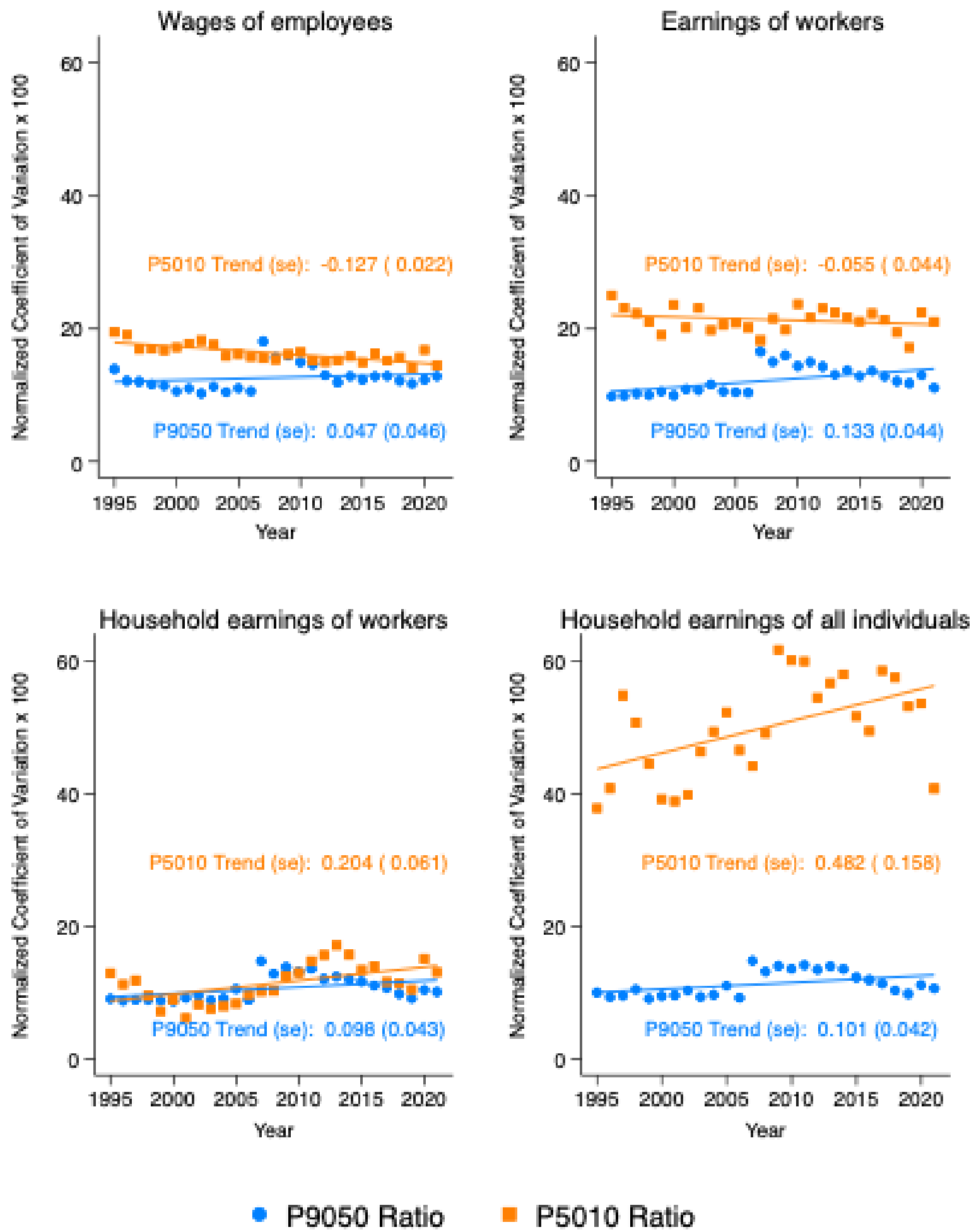


**Fig. S4.** Tests of Convergence in Upper-Tail and Lower-Tail Labor-Market Inequalities among Working-Age Population. Inequality is measured by the ratio of the 90$^{th}$ to 50$^{th}$ percentiles (upper tail) and ratio of the 50$^{th}$ to 10$^{th}$ percentiles (lower tail). Dispersion is measured by the normalized coefficient of variation of inequality ($\boldsymbol{NCV} = \frac{\boldsymbol{CV}}{1+\boldsymbol{CV}}$) times 100, where CV is the ratio of the standard deviation to the mean, $\boldsymbol{CV} = {}^{\boldsymbol{\sigma}}/_{\boldsymbol{\mu}}$. The test of convergence is represented by the coefficient and associated standard error (se) on the linear trend line through the NCV in the figure. Wages of employees are average hourly earnings for employees. Earnings of workers are annual earnings of employees and self-employed workers. Household earnings of workers add up annual earnings of all persons who are employees or self-employed workers. Household earnings of all individuals include non-working households. Household earnings are equivalized using the Modified OECD scale. Working-age population is defined as those aged 25-60.

### 3. Inequality not Adjusted for Demographic Composition

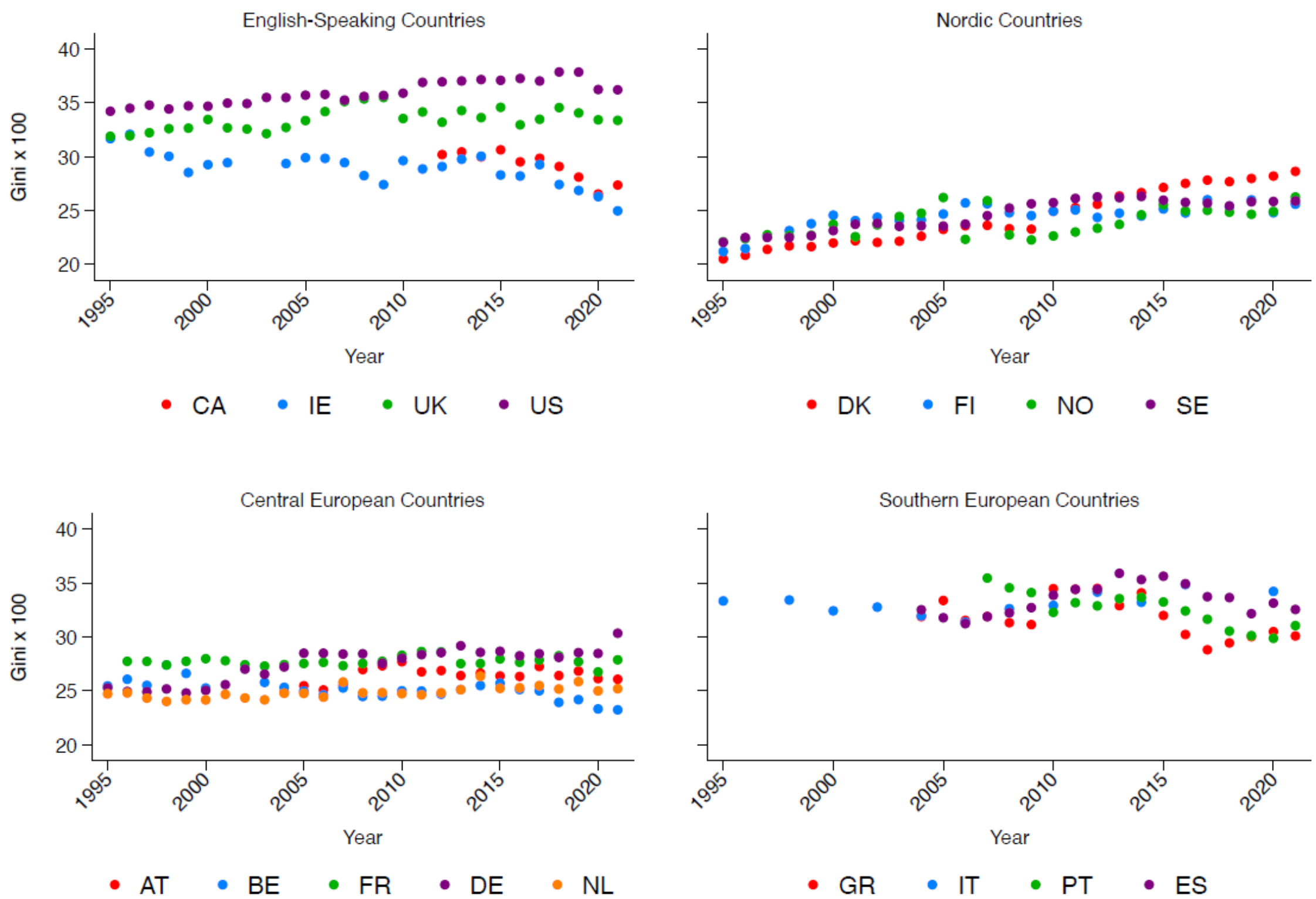


**Fig. S5.** Cross-Country Trends in Disposable Income Inequality among the Working Age Population without Demographic Adjustment. Inequality is measured by the Gini coefficient times 100. Disposable income is the sum of market income from labor earnings, ordinary capital income, nongovernmental transfers, and governmental cash and near-cash transfer income, less tax payments inclusive of refundable credits. Income includes contributions from household members of all ages, and is equivalized using the Modified OECD scale. The sample used in the figure is the working-age population defined as those aged 25-60. Countries are grouped into English-speaking nations of Canada (CA), Ireland (IE), United Kingdom (UK), and United States (US); Nordic countries of Denmark (DK), Finland (FI), Norway (NO), and Sweden (SE); Central European countries of Austria (AT), Belgium (BE), France (FR), Germany (DE), and The Netherlands (NL); and Southern European nations of Greece (GR), Italy (IT), Portugal (PT), and Spain (ES).

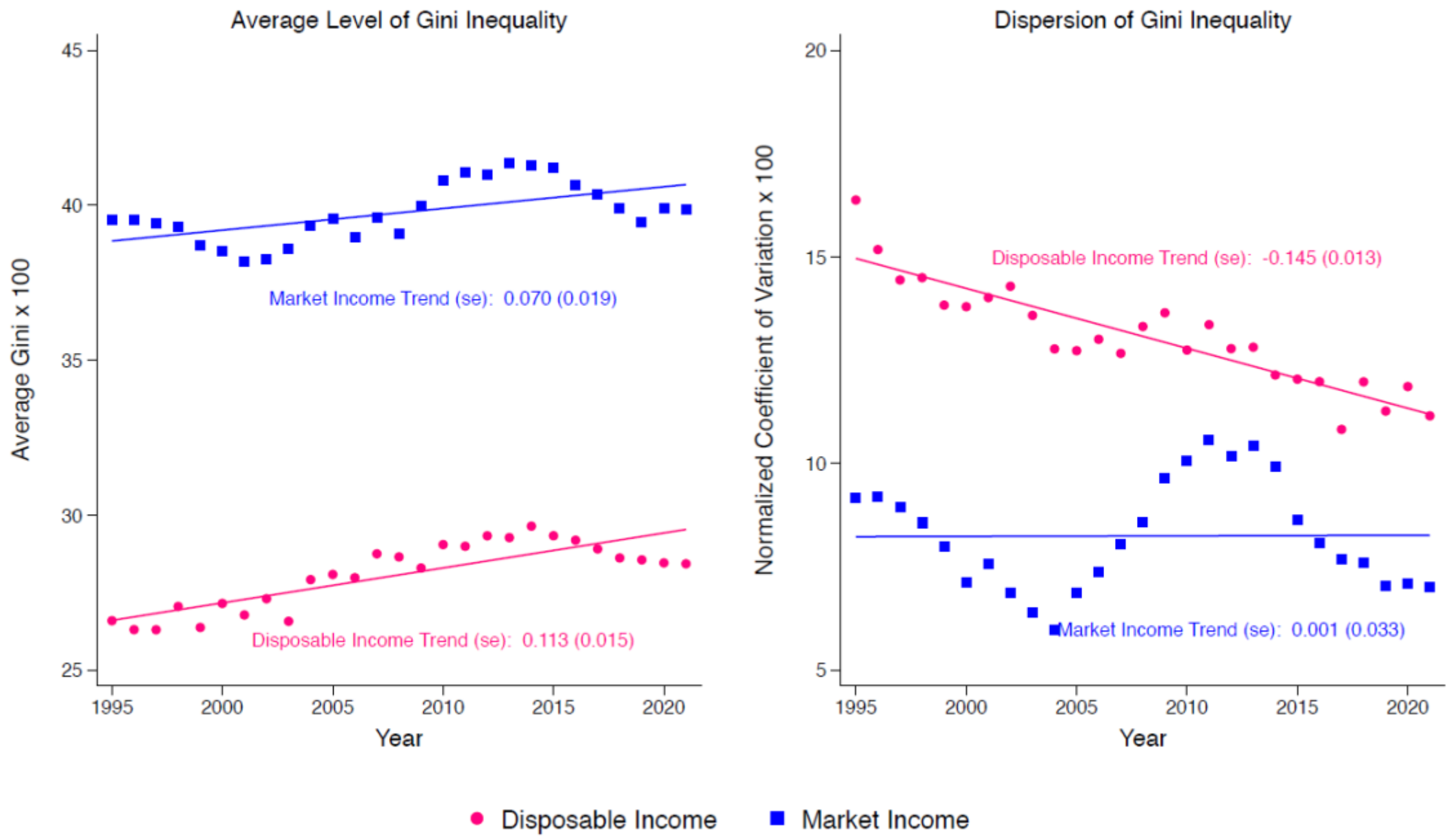


**Fig. S6.** Average Level of and Convergence in Cross-Country Disposable and Market Income Inequality among the Working-Age Population without Demographic Adjustment. Inequality is measured by the Gini coefficient times 100. Dispersion is measured by the normalized coefficient of variation of inequality ($\boldsymbol{NCV} = \frac{CV}{1+CV}$) times 100, where CV is the ratio of the standard deviation to the mean, $\boldsymbol{CV} = {}^{\sigma}/_{\mu}$. The test of convergence is represented by the coefficient and associated standard error (se) on the linear trend line through the NCV in the figure. Market income is the sum of labor earnings, ordinary capital income, and nongovernmental transfers. Disposable income is market income plus cash and near-cash governmental transfers less tax payments inclusive of refundable credits. Income includes contributions from household members of all ages, and is equivalized using the Modified OECD scale. Working-age population is defined as those aged 25-60.

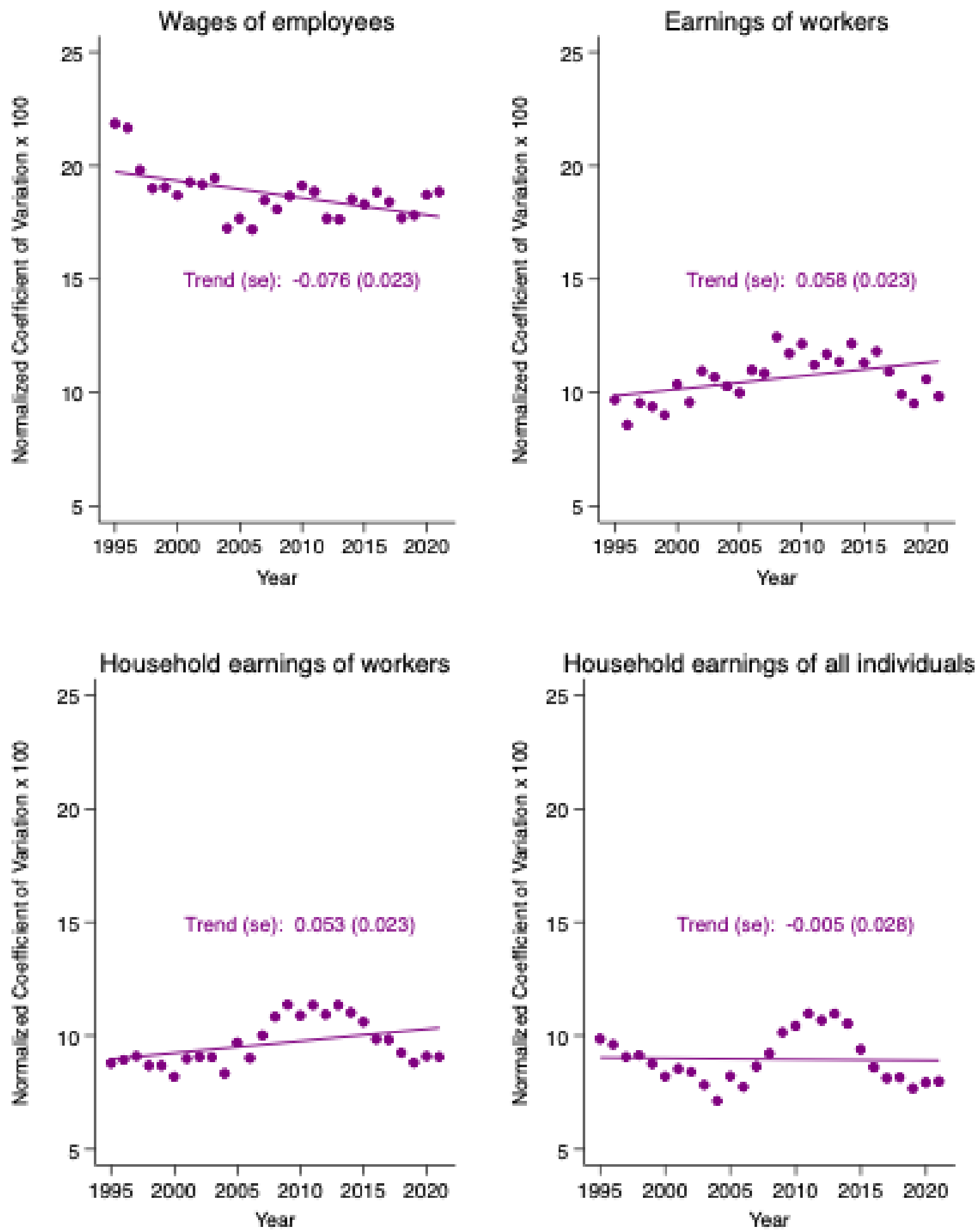


**Fig. S7.** Tests of Convergence in Cross-Country Labor-Market Inequalities among Working-Age Population without Demographic Adjustment. Inequality is measured by the Gini coefficient times 100. Dispersion is measured by the normalized coefficient of variation of inequality ($\boldsymbol{NCV} = \frac{CV}{1+CV}$) times 100, where CV is the ratio of the standard deviation to the mean, $\boldsymbol{CV} = \sigma/\mu$. The test of convergence is represented by the coefficient and associated standard error (se) on the linear trend line through the NCV in the figure. Wages of employees are average hourly earnings for employees. Earnings of workers are annual earnings of employees and self-employed workers. Household earnings of workers add up annual earnings of all persons who are employees or self-employed workers. Household earnings of all individuals include non-working households. Household earnings are equivalized using the Modified OECD scale. Working-age population is defined as those aged 25-60.

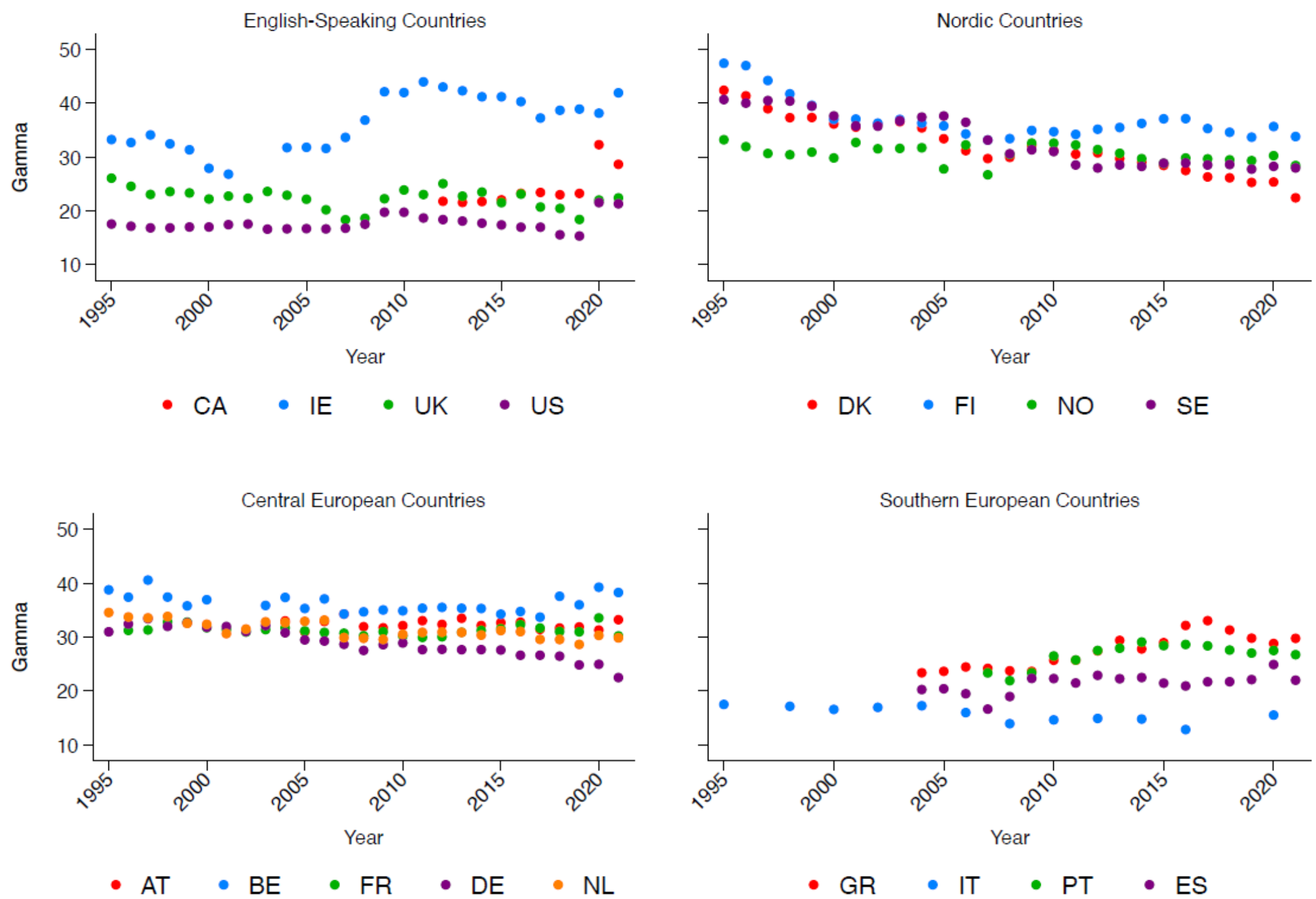


**Fig. S8.** Cross-Country Trends in Redistribution from Market Income to Disposable Income for Working-Age Population without Demographic Adjustment. Inequality is measured by the Gini coefficient. Gamma is 100 times the relative reduction in market income inequality to disposable income inequality defined as $\hat{\gamma}_{j,t}^{Gini} = 100 * (1 - \frac{\hat{I}_{j,t}^{Disp\ Gini}}{\hat{I}_{j,t}^{Market\ Gini}})$. Market income is the sum of labor earnings, ordinary capital income, and nongovernmental transfers. Disposable income is market income plus cash and near-cash governmental transfers less tax payments inclusive of refundable credits. Income includes contributions from household members of all ages, and is equivalized using the Modified OECD scale. Working-age population is defined as those aged 25-60. Countries are grouped into English-speaking nations of Canada (CA), Ireland (IE), United Kingdom (UK), and United States (US); Nordic countries of Denmark (DK), Finland (FI), Norway (NO), and Sweden (SE); Central European countries of Austria (AT), Belgium (BE), France (FR), Germany (DE), and The Netherlands (NL); and Southern European nations of Greece (GR), Italy (IT), Portugal (PT), and Spain (ES).

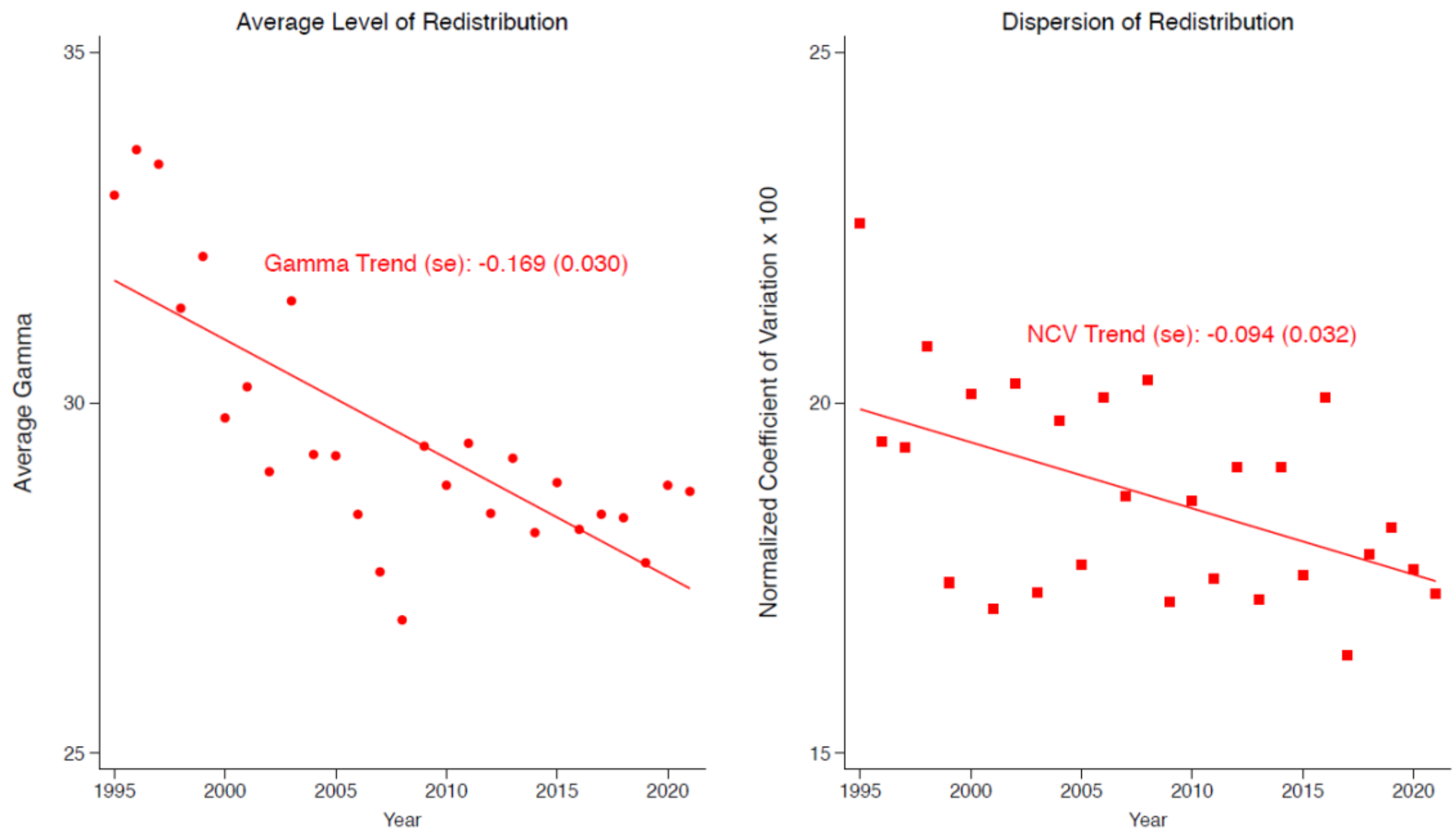


**Fig. S9.** Average Level of and Convergence in Cross-Country Redistribution from Market Income to Disposable Income among the Working-Age Population without Demographic Adjustment. Average Gamma is the cross-country average of 100 times the relative reduction in market income inequality to disposable income inequality defined as $\hat{\boldsymbol{\gamma}}_{j,t}^{Gini} = \mathbf{100} * (\mathbf{1} - \frac{\hat{I}_{j,t}^{Disp\ Gini}}{\hat{I}_{j,t}^{Market\ Gini}})$. Inequality is measured by the Gini coefficient. Dispersion is measured by the normalized coefficient of variation of gamma ($\boldsymbol{NCV} = \frac{CV}{1+CV}$) times 100, where CV is the ratio of the standard deviation to the mean, $\boldsymbol{CV} = {}^{\sigma}/_{\mu}$. The test of convergence is represented by the coefficient and associated standard error (se) on the linear trend line through the NCV in the figure. Market income is the sum of labor earnings, ordinary capital income, and nongovernmental transfers. Disposable income is market income plus cash and near-cash governmental transfers less tax payments inclusive of refundable credits. Income includes contributions from household members of all ages, and is equivalized using the Modified OECD scale. Working-age population is defined as those aged 25-60.

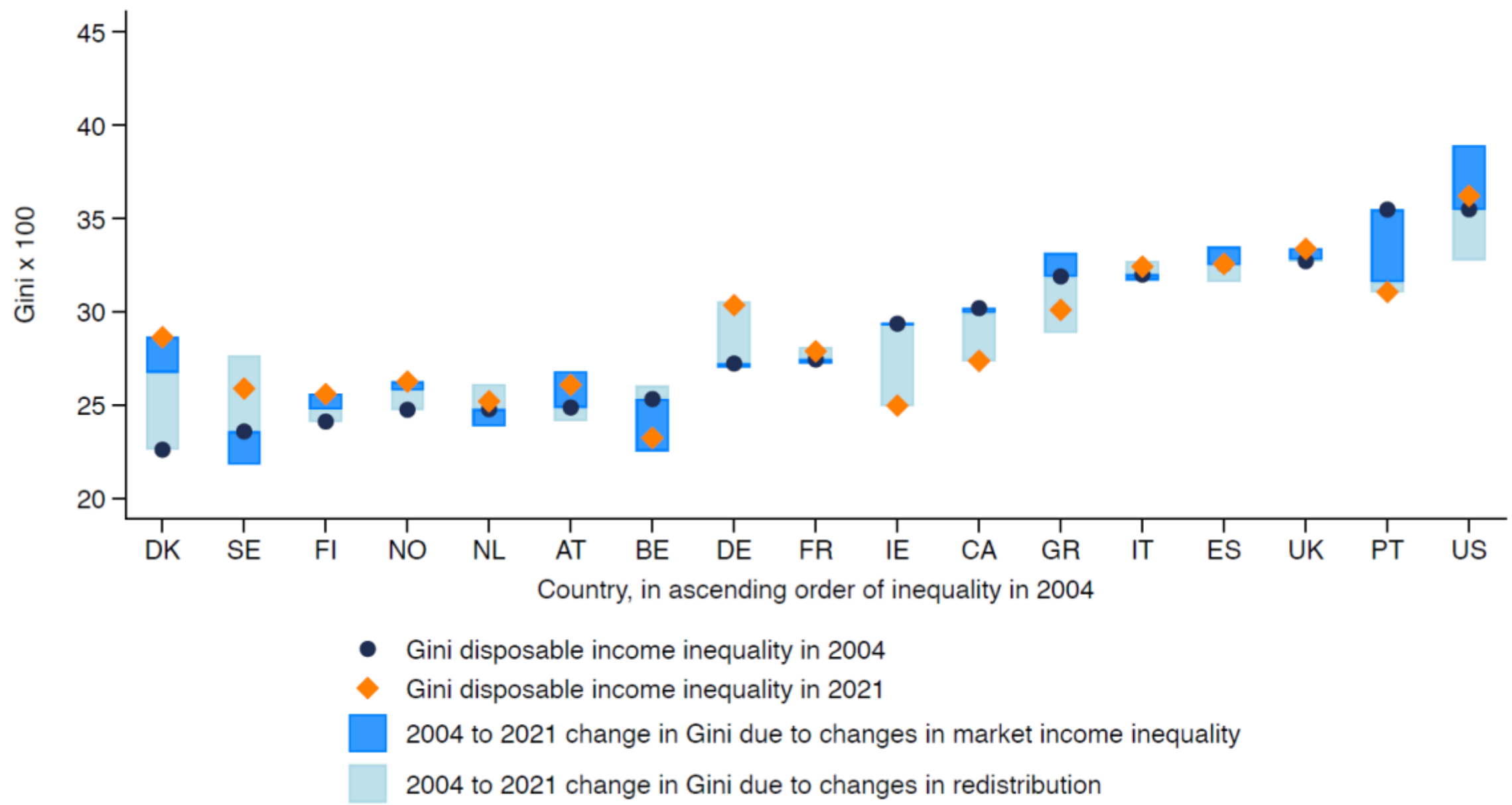


**Fig. S10.** Decomposition of the Change in Disposable Income Inequality from 2004-2021 among the Working-Age Population without Demographic Adjustment. Inequality is measured by the Gini coefficient times 100. Market income is the sum of labor earnings, ordinary capital income, and nongovernmental transfers, and the mid-blue color bar reflects the change in market income inequality from 2004-2021. Disposable income is market income plus cash and near-cash governmental transfers less tax payments inclusive of refundable credits. Redistribution is the change in income inequality in moving from market income inequality to disposable income inequality as reflected in the light-blue color bar. See equation (4) in *Materials and Methods* for additional details. Income includes contributions from household members of all ages, and is equivalized using the Modified OECD scale. Working-age population is defined as those aged 25-60. Countries are listed in ascending order of initial 2004 disposable income inequality as Denmark (DK), Sweden (SE), Finland (FI), Norway (NO), The Netherlands (NL), Austria (AT), Belgium (BE), Germany (DE), France (FR), Ireland (IE), Canada (CA), Greece (GR), Italy (IT), Spain (ES), United Kingdom (UK), Portugal (PT), and United States (US). For CA, income in 2004 is from 2012; for IT, income in 2021 is from 2022; and for PT, income in 2004 is from 2007.

## 4. Alternative Measures of Inequality

A. Ratio of Average income in Top 5% to Bottom 25% (T5-B25 Ratio)

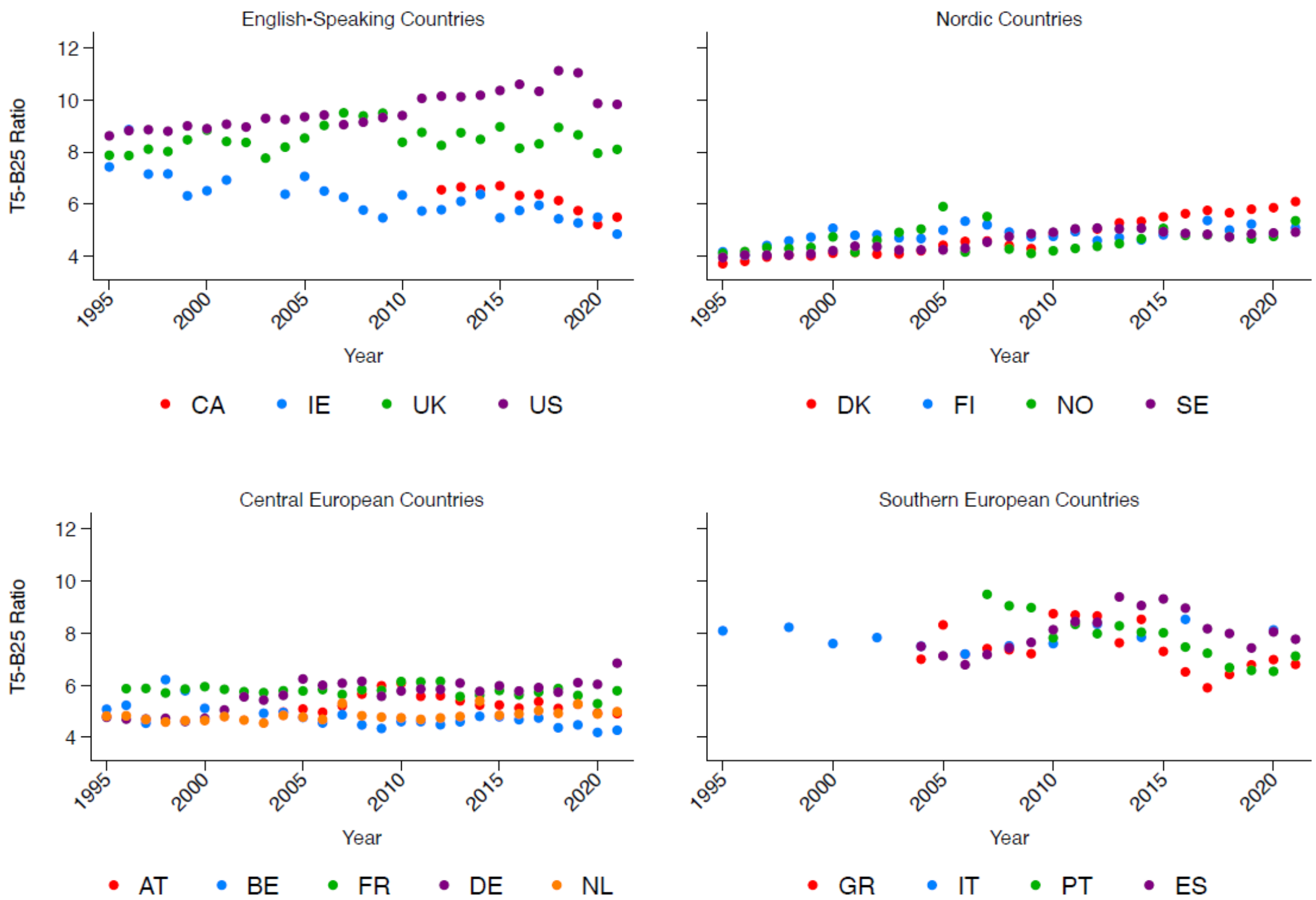


**Fig. S11.** Cross-Country Trends in Disposable Income Inequality among the Working-Age Population. Inequality is measured by the T5-B25 Ratio, which is the average income received by the top 5% of households divided by the average income received by the bottom 25% of households. Disposable income is the sum of market income from labor earnings, ordinary capital income, nongovernmental transfers, and governmental cash and near-cash transfer income, less tax payments inclusive of refundable credits. Income includes contributions from household members of all ages, and is equivalized using the Modified OECD scale. The sample used in the figure is the working-age population defined as those aged 25-60. Countries are grouped into English-speaking nations of Canada (CA), Ireland (IE), United Kingdom (UK), and United States (US); Nordic countries of Denmark (DK), Finland (FI), Norway (NO), and Sweden (SE); Central European countries of Austria (AT), Belgium (BE), France (FR), Germany (DE), and The Netherlands (NL); and Southern European nations of Greece (GR), Italy (IT), Portugal (PT), and Spain (ES).

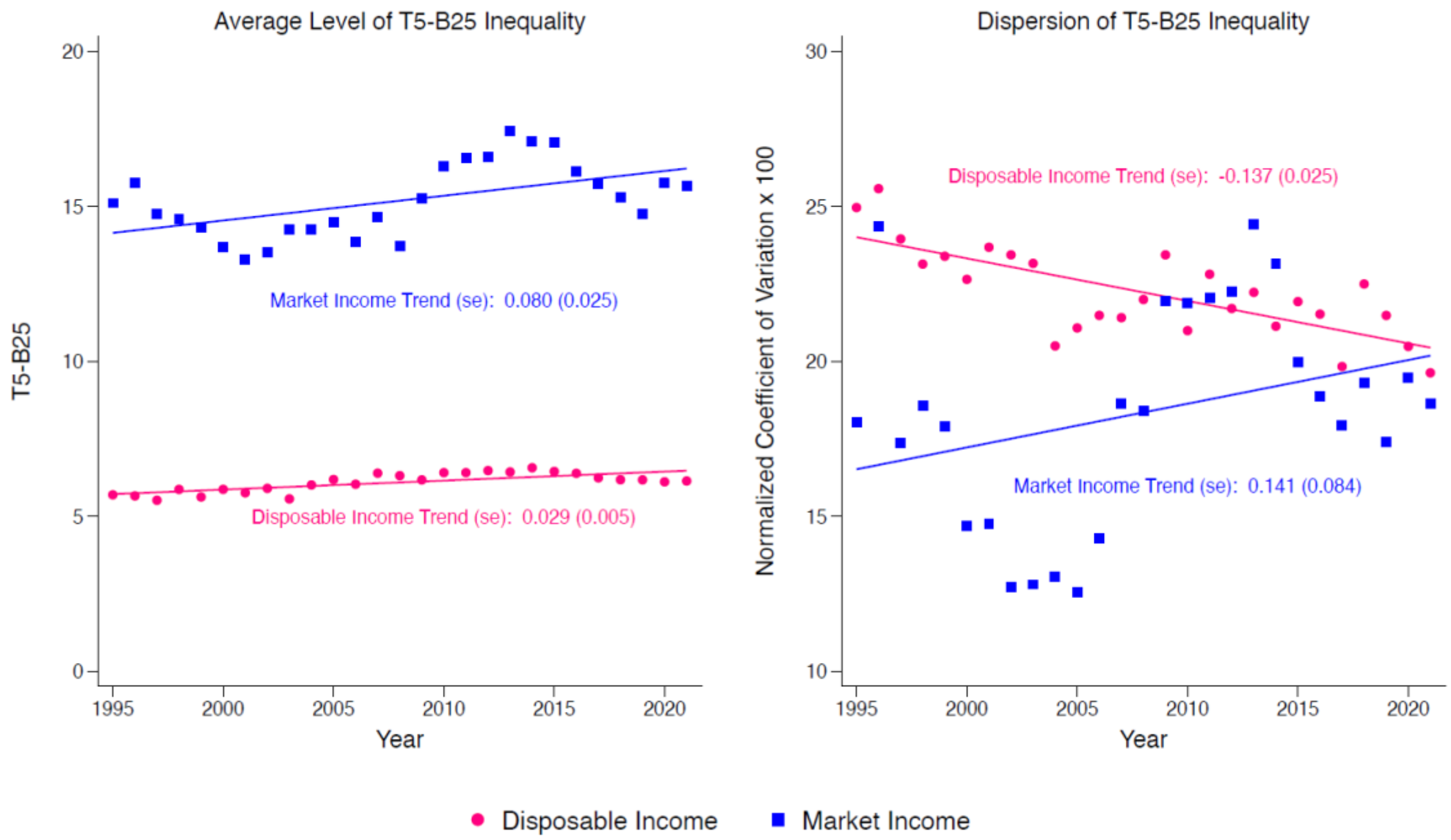


**Fig. S12.** Average Level of and Convergence in Cross-Country Disposable and Market Income Inequality among the Working-Age Population. Inequality is measured by the T5-B25 Ratio, which is the average income received by the top 5% of households divided by the average income received by the bottom 25% of households. Dispersion is measured by the normalized coefficient of variation of inequality ($\boldsymbol{NCV} = \frac{\boldsymbol{CV}}{\boldsymbol{1+CV}}$) times 100, where CV is the ratio of the standard deviation to the mean, $\boldsymbol{CV} = {}^{\sigma}/_{\mu}$. The test of convergence is represented by the coefficient and associated standard error (se) on the linear trend line through the NCV in the figure. Market income is the sum of labor earnings, ordinary capital income, and nongovernmental transfers. Disposable income is market income plus cash and near-cash governmental transfers less tax payments inclusive of refundable credits. Income includes contributions from household members of all ages, and is equivalized using the Modified OECD scale. Working-age population is defined as those aged 25-60.

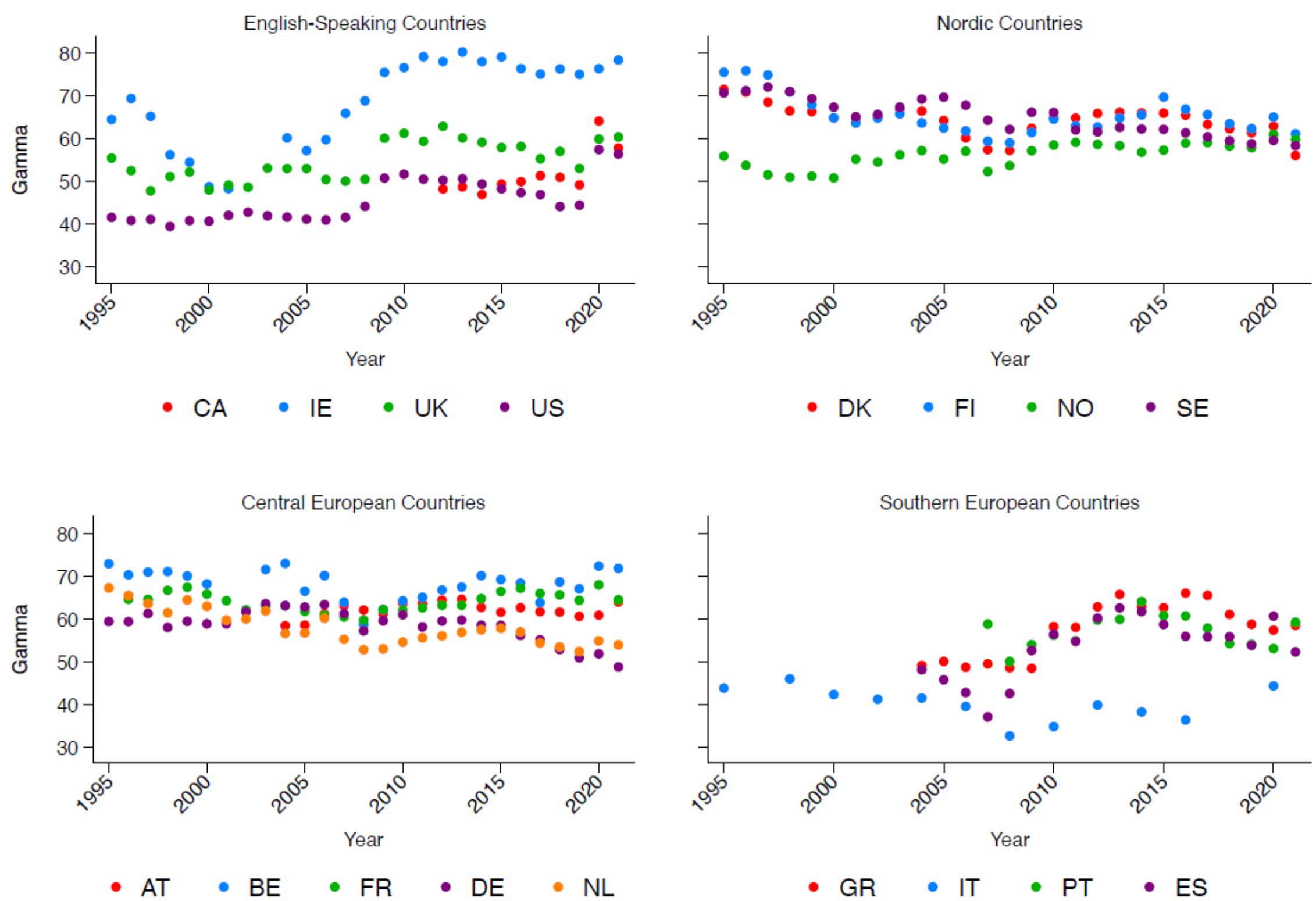


**Fig. S13.** Cross-Country Trends in Redistribution from Market Income to Disposable Income for Working-Age Population. Inequality is measured by the T5-B25 Ratio, which is the average income received by the top 5% of households divided by the average income received by the bottom 25% of households. Gamma is 100 times the relative reduction in market income inequality to disposable income inequality from redistribution defined as $\hat{\gamma}_{j,t}^{T5-B25} = 100 * \left(1 - \frac{\hat{I}_{j,t}^{Disp\,T5-B25}}{\hat{I}_{j,t}^{Market\,T5-B25}}\right)$. Market income is the sum of labor earnings, ordinary capital income, and nongovernmental transfers. Disposable income is market income plus cash and near-cash governmental transfers less tax payments inclusive of refundable credits. Income includes contributions from household members of all ages, and is equivalized using the Modified OECD scale. Working-age population is defined as those aged 25-60. Countries are grouped into English-speaking nations of Canada (CA), Ireland (IE), United Kingdom (UK), and United States (US); Nordic countries of Denmark (DK), Finland (FI), Norway (NO), and Sweden (SE); Central European countries of Austria (AT), Belgium (BE), France (FR), Germany (DE), and The Netherlands (NL); and Southern European nations of Greece (GR), Italy (IT), Portugal (PT), and Spain (ES).

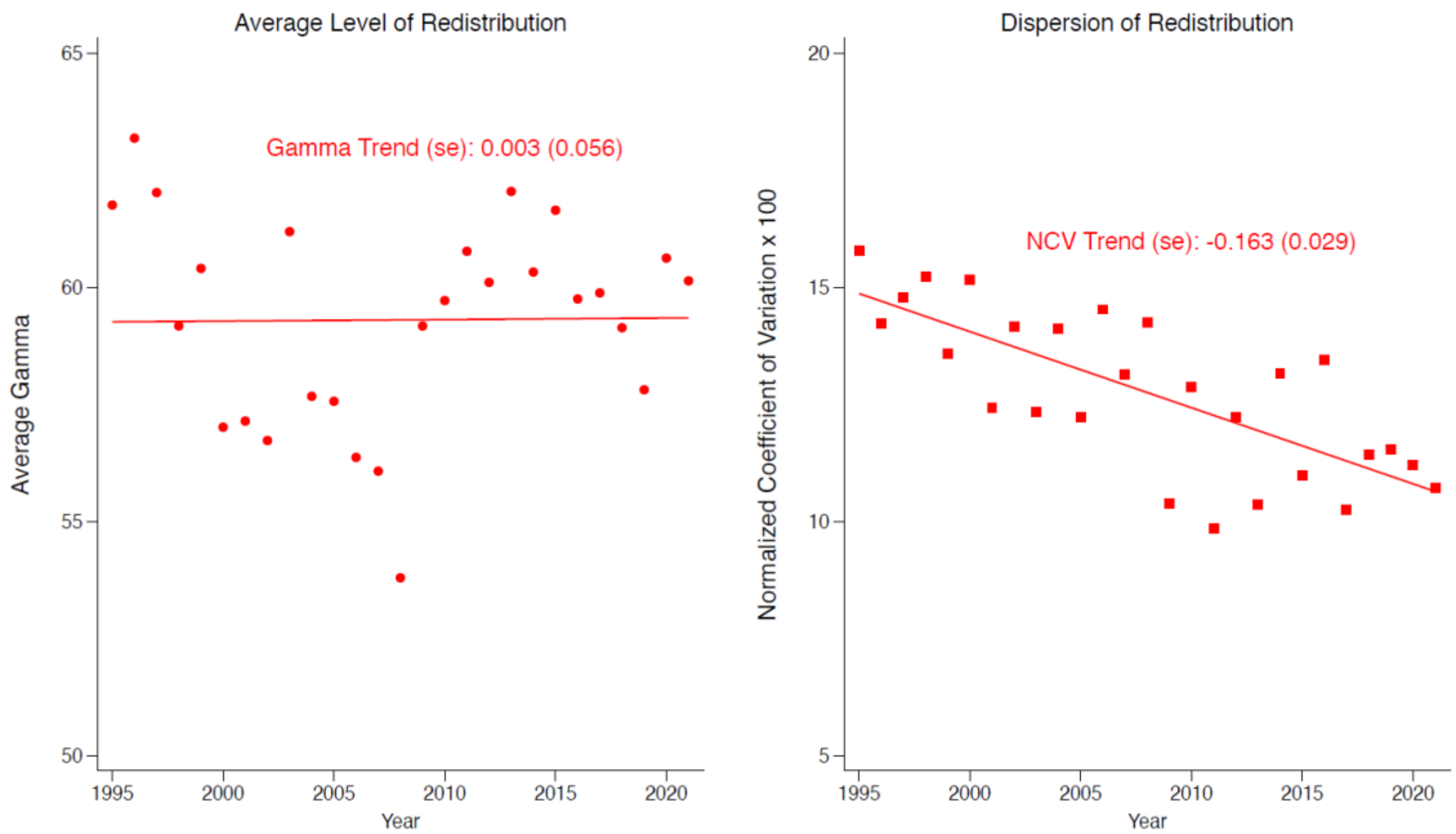


**Fig. S14.** Average Level of and Convergence in Cross-Country Redistribution from Market Income to Disposable Income among the Working-Age Population. Average Gamma is the cross-country average of 100 times the relative reduction in market income inequality to disposable income inequality from redistribution. Inequality is measured by the T5-B25 Ratio, which is the average income received by the top 5% of households divided by the average income received by the bottom 25% of households. Dispersion is measured by the normalized coefficient of variation of gamma ($NCV = \frac{CV}{1+CV}$) times 100, where CV is the ratio of the standard deviation to the mean, $CV = {}^{\sigma}/_{\mu}$. The test of convergence is represented by the coefficient and associated standard error (se) on the linear trend line through the NCV in the figure. Market income is the sum of labor earnings, ordinary capital income, and nongovernmental transfers. Disposable income is market income plus cash and near-cash governmental transfers less tax payments inclusive of refundable credits. Income includes contributions from household members of all ages, and is equivalized using the Modified OECD scale. Working-age population is defined as those aged 25-60.

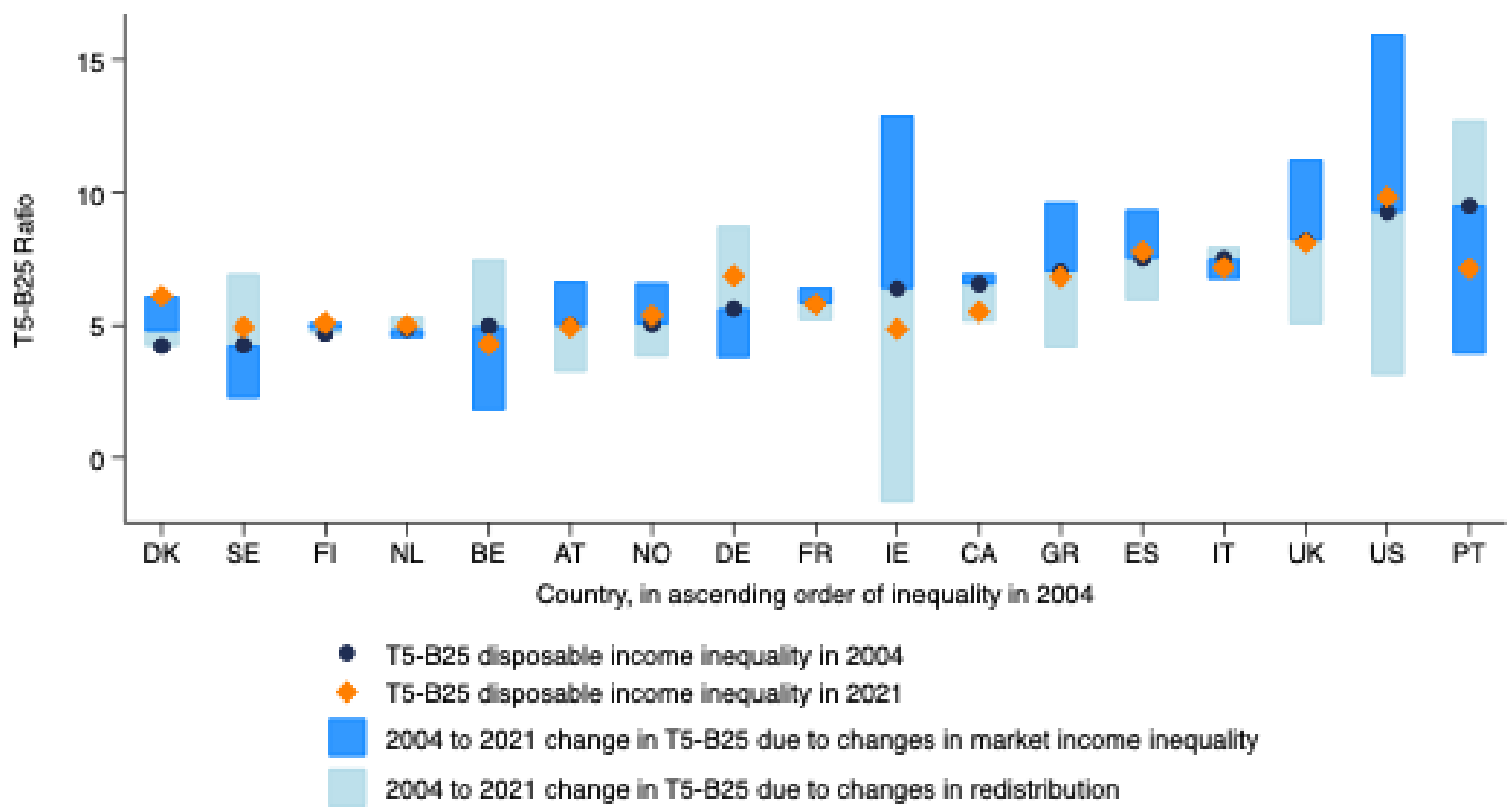


**Fig. S15.** Decomposition of the Change in Disposable Income Inequality from 2004-2021 among the Working-Age Population. Inequality is measured by the T5-B25 Ratio, which is the average income received by the top 5% of households divided by the average income received by the bottom 25% of households. Market income is the sum of labor earnings, ordinary capital income, and nongovernmental transfers, and the mid-blue color bar reflects the change in market income inequality from 2004-2021. Disposable income is market income plus cash and near-cash governmental transfers less tax payments inclusive of refundable credits. Redistribution is the change in income inequality in moving from market income inequality to disposable income inequality as reflected in the light-blue color bar. See equation (4) in *Materials and Methods* for additional details. Income includes contributions from household members of all ages, and is equivalized using the Modified OECD scale. Working-age population is defined as those aged 25-60. Countries are listed in ascending order of initial 2004 disposable income inequality as Denmark (DK), Sweden (SE), Finland (FI), The Netherlands (NL), Belgium (BE), Austria (AT), Norway (NO), Germany (DE), France (FR), Ireland (IE), Canada (CA), Greece (GR), Spain (ES), Italy (IT), United Kingdom (UK), United States (US), and Portugal (PT). For CA, income in 2004 is from 2012; for IT, income in 2021 is from 2022; and for PT, income in 2004 is from 2007.

B. Ratio of average income in Top 5% to Middle 70% (T5-M70 Ratio)

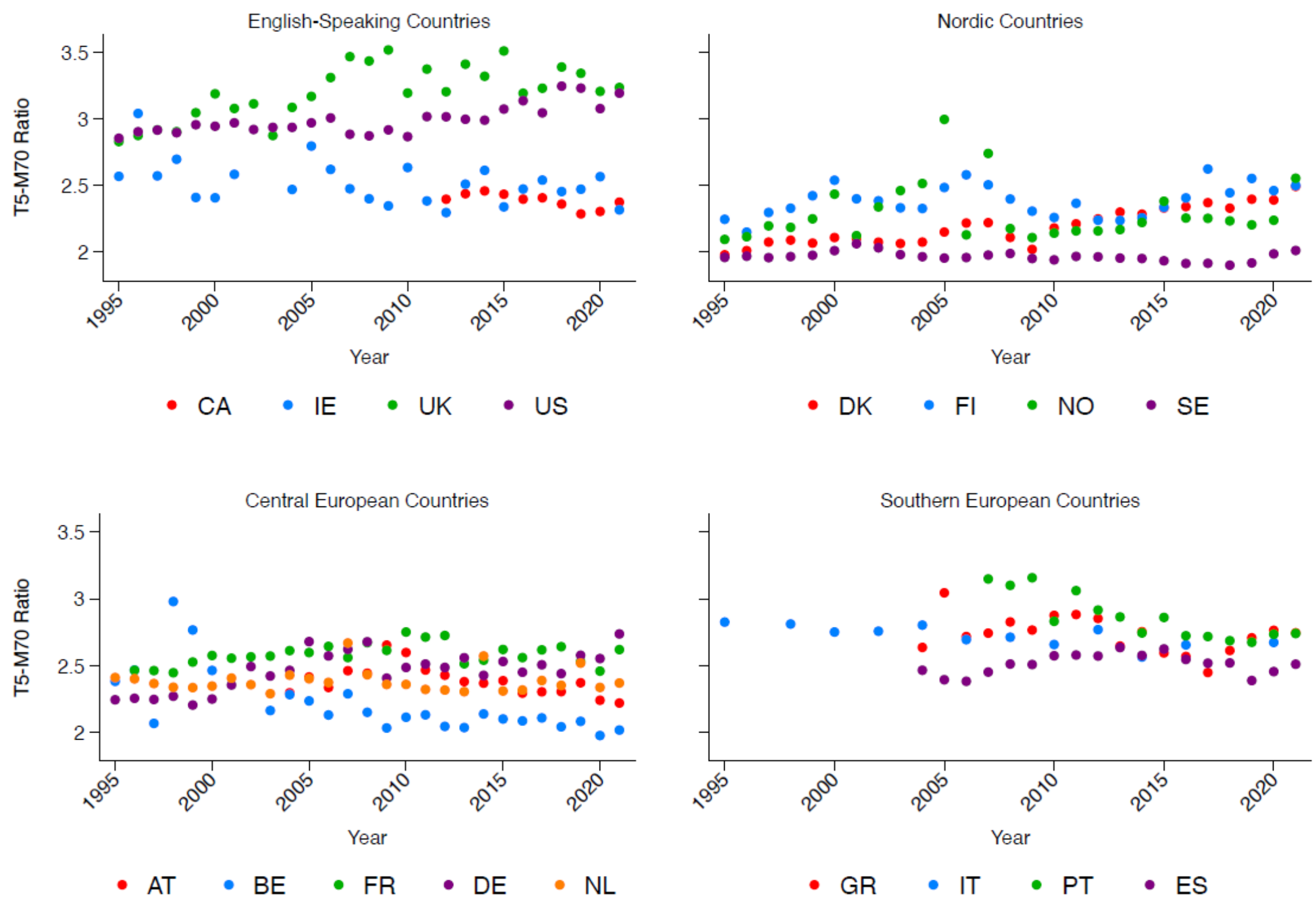


**Fig. S16.** Cross-Country Trends in Disposable Income Inequality among the Working-Age Population. Inequality is measured by the T5-M70 Ratio, which is the average income received by the top 5% of households divided by the average income received by the middle 70% of households. Disposable income is the sum of market income from labor earnings, ordinary capital income, nongovernmental transfers, and governmental cash and near-cash transfer income, less tax payments inclusive of refundable credits. Income includes contributions from household members of all ages, and is equivalized using the Modified OECD scale. The sample used in the figure is the working-age population defined as those aged 25-60. Countries are grouped into English-speaking nations of Canada (CA), Ireland (IE), United Kingdom (UK), and United States (US); Nordic countries of Denmark (DK), Finland (FI), Norway (NO), and Sweden (SE); Central European countries of Austria (AT), Belgium (BE), France (FR), Germany (DE), and The Netherlands (NL); and Southern European nations of Greece (GR), Italy (IT), Portugal (PT), and Spain (ES).

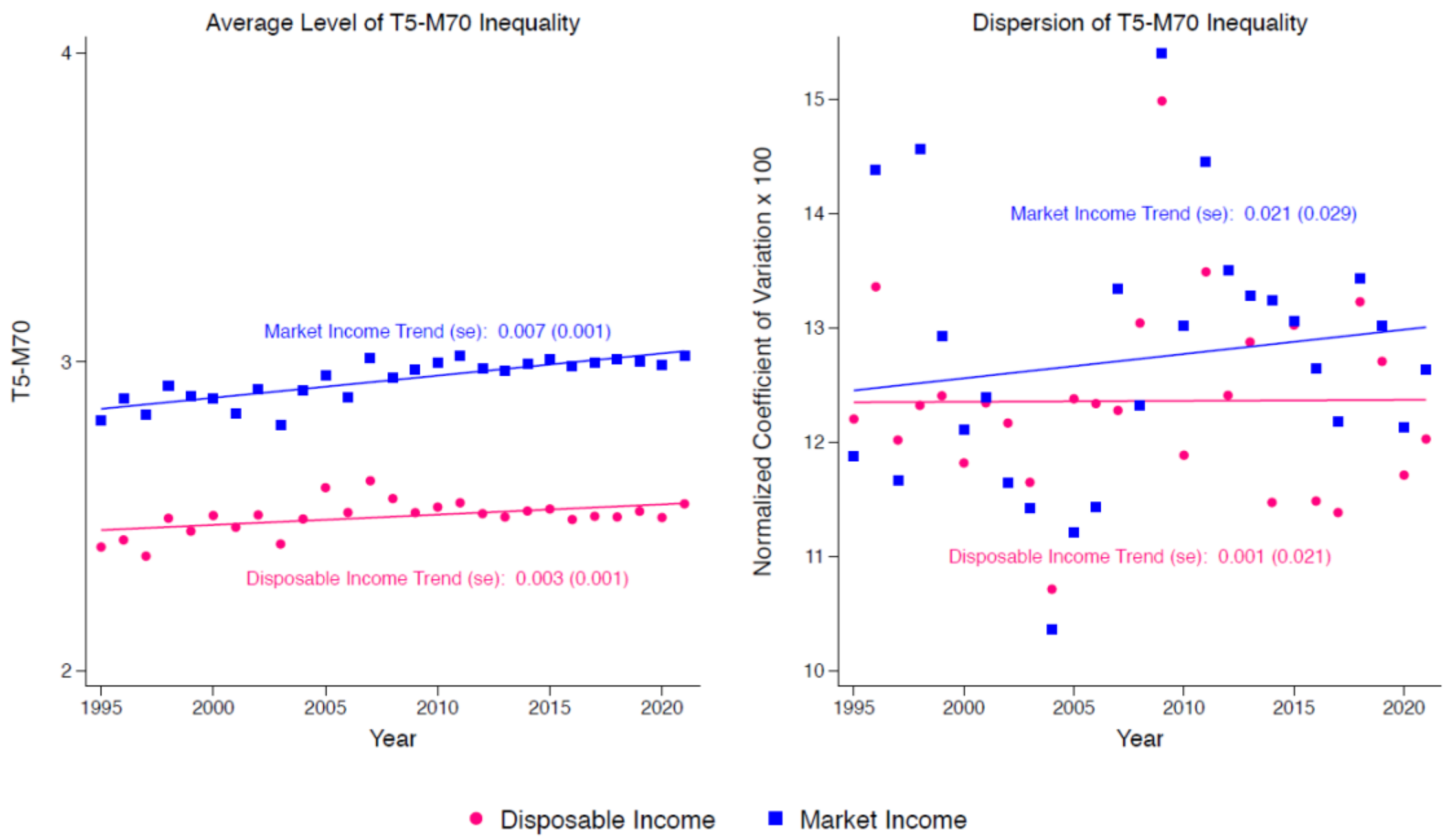


**Fig. S17.** Average Level of and Convergence in Cross-Country Disposable and Market Income Inequality among the Working-Age Population. Inequality is measured by the T5-M70 Ratio, which is the average income received by the top 5% of households divided by the average income received by the middle 70% of households. Dispersion is measured by the normalized coefficient of variation of inequality ($\boldsymbol{NCV} = \frac{\boldsymbol{CV}}{\boldsymbol{1+CV}}$) times 100, where CV is the ratio of the standard deviation to the mean, $\boldsymbol{CV} = {}^{\boldsymbol{\sigma}}/_{\boldsymbol{\mu}}$. The test of convergence is represented by the coefficient and associated standard error (se) on the linear trend line through the NCV in the figure. Market income is the sum of labor earnings, ordinary capital income, and nongovernmental transfers. Disposable income is market income plus cash and near-cash governmental transfers less tax payments inclusive of refundable credits. Income includes contributions from household members of all ages, and is equivalized using the Modified OECD scale. Working-age population is defined as those aged 25-60.

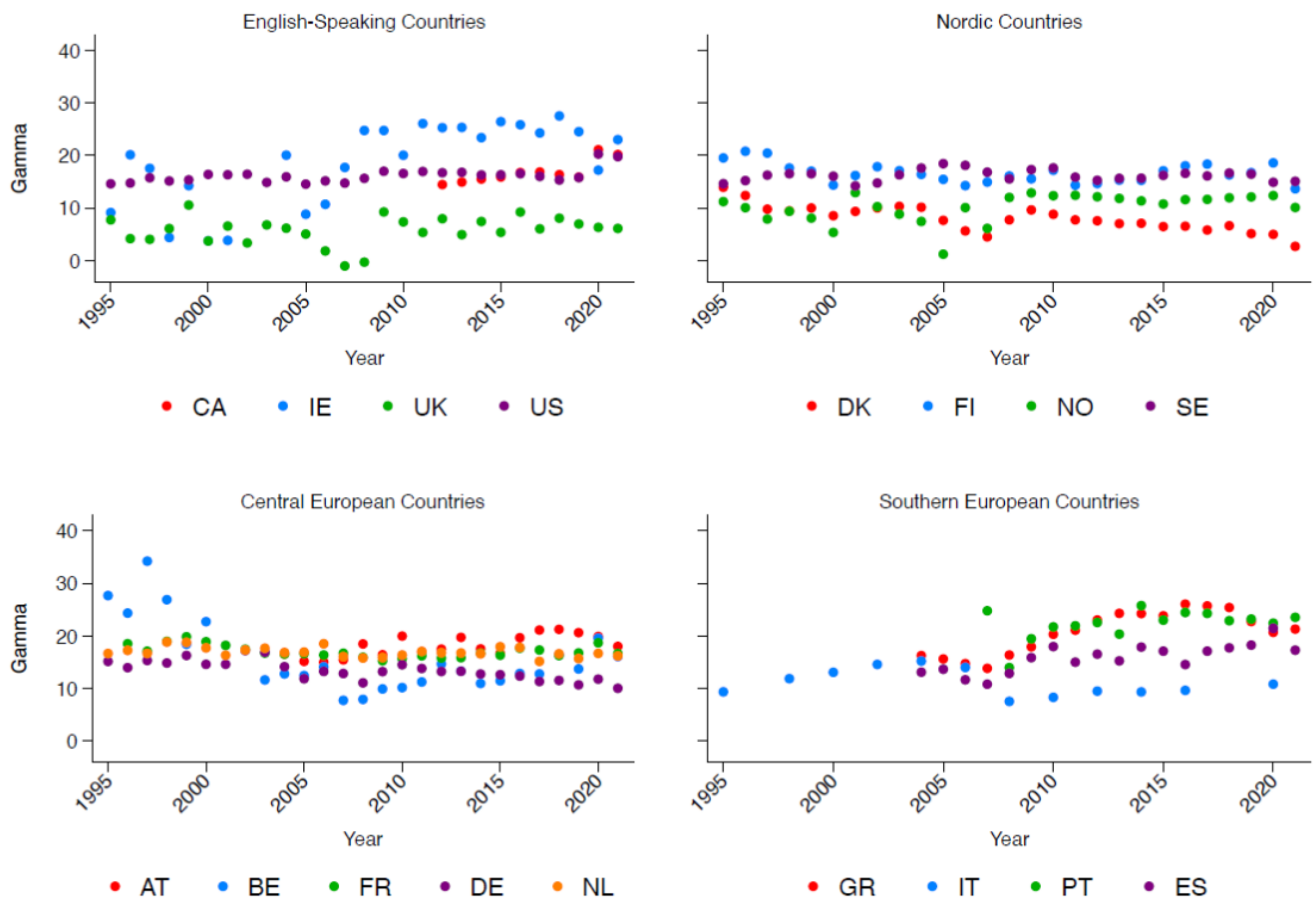


**Fig. S18.** Cross-Country Trends in Redistribution from Market Income to Disposable Income for Working-Age Population. Inequality is measured by the T5-M70 Ratio, which is the average income received by the top 5% of households divided by the average income received by the middle 70% of households. Gamma is 100 times the relative reduction in market income inequality to disposable income inequality from redistribution defined as $\hat{\gamma}_{j,t}^{T5-M70} = 100 * (1 - \frac{\hat{I}_{j,t}^{Disp\,T5-M70}}{\hat{I}_{j,t}^{Market\,T5-M70}})$. Market income is the sum of labor earnings, ordinary capital income, and nongovernmental transfers. Disposable income is market income plus cash and near-cash governmental transfers less tax payments inclusive of refundable credits. Income includes contributions from household members of all ages, and is equivalized using the Modified OECD scale. Working-age population is defined as those aged 25-60. Countries are grouped into English-speaking nations of Canada (CA), Ireland (IE), United Kingdom (UK), and United States (US); Nordic countries of Denmark (DK), Finland (FI), Norway (NO), and Sweden (SE); Central European countries of Austria (AT), Belgium (BE), France (FR), Germany (DE), and The Netherlands (NL); and Southern European nations of Greece (GR), Italy (IT), Portugal (PT), and Spain (ES).

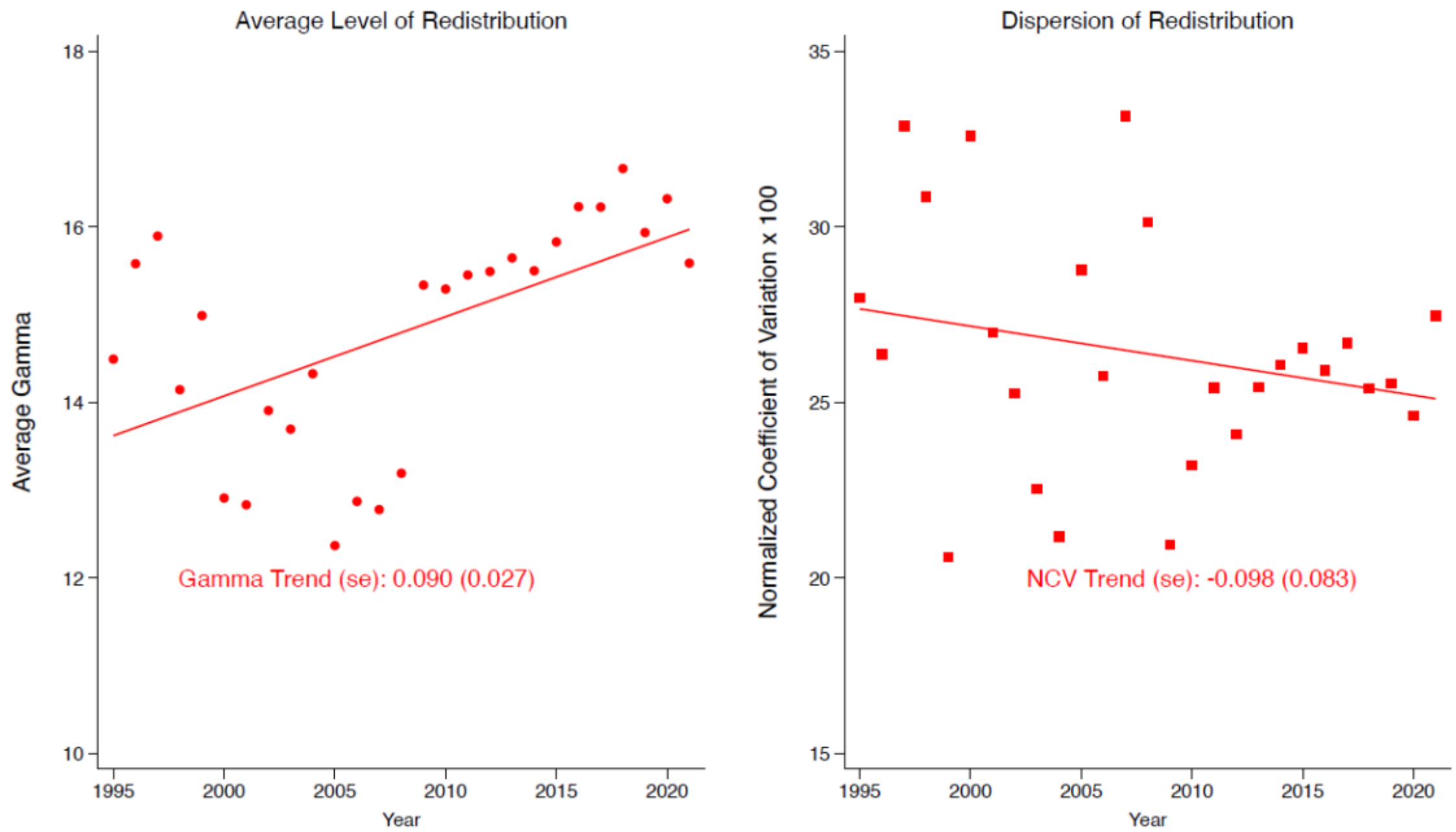


**Fig. S19.** Average Level of and Convergence in Cross-Country Redistribution from Market Income to Disposable Income among the Working-Age Population. Average Gamma is the cross-country average of 100 times the relative reduction in market income inequality to disposable income inequality from redistribution. Inequality is measured by the T5-M70 Ratio, which is the average income received by the top 5% of households divided by the average income received by the middle 70% of households. Dispersion is measured by the normalized coefficient of variation of gamma ($NCV = \frac{CV}{1+CV}$) times 100, where CV is the ratio of the standard deviation to the mean, $CV = {}^{\sigma}/_{\mu}$. The test of convergence is represented by the coefficient and associated standard error (se) on the linear trend line through the NCV in the figure. Market income is the sum of labor earnings, ordinary capital income, and nongovernmental transfers. Disposable income is market income plus cash and near-cash governmental transfers less tax payments inclusive of refundable credits. Income includes contributions from household members of all ages, and is equivalized using the Modified OECD scale. Working-age population is defined as those aged 25-60.

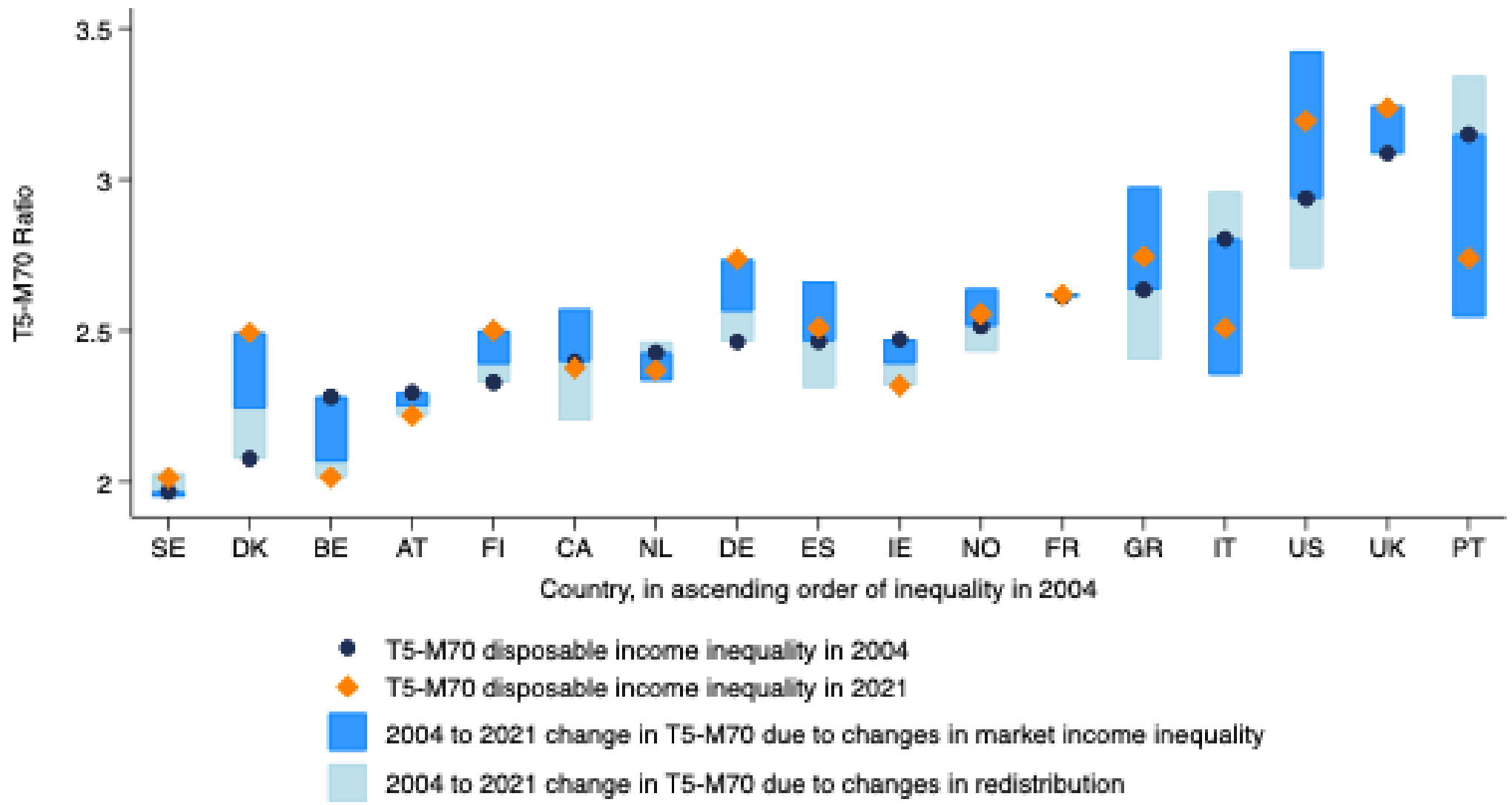


**Fig. S20.** Decomposition of the Change in Disposable Income Inequality from 2004-2021 among the Working-Age Population. Inequality is measured by the T5-M70 Ratio, which is the average income received by the top 5% of households divided by the average income received by the middle 70% of households. Market income is the sum of labor earnings, ordinary capital income, and nongovernmental transfers, and the mid-blue color bar reflects the change in market income inequality from 2004-2021. Disposable income is market income plus cash and near-cash governmental transfers less tax payments inclusive of refundable credits. Redistribution is the change in income inequality in moving from market income inequality to disposable income inequality as reflected in the light-blue color bar. See equation (4) in *Materials and Methods* for additional details. Income includes contributions from household members of all ages, and is equivalized using the Modified OECD scale. Working-age population is defined as those aged 25-60. Countries are listed in ascending order of initial 2004 disposable income inequality as Sweden (SE), Denmark (DK), Belgium (BE), Austria (AT), Finland (FI), Canada (CA), The Netherlands (NL), Germany (DE), Spain (ES), Ireland (IE), Norway (NO), France (FR), Greece (GR), Italy (IT), United Kingdom (UK), United States (US), and Portugal (PT). For CA, income in 2004 is from 2012; for IT, income in 2021 is from 2022; and for PT, income in 2004 is from 2007.

C. Ratio of Average income in Middle 70% to Bottom 25% (M70-B25 Ratio)

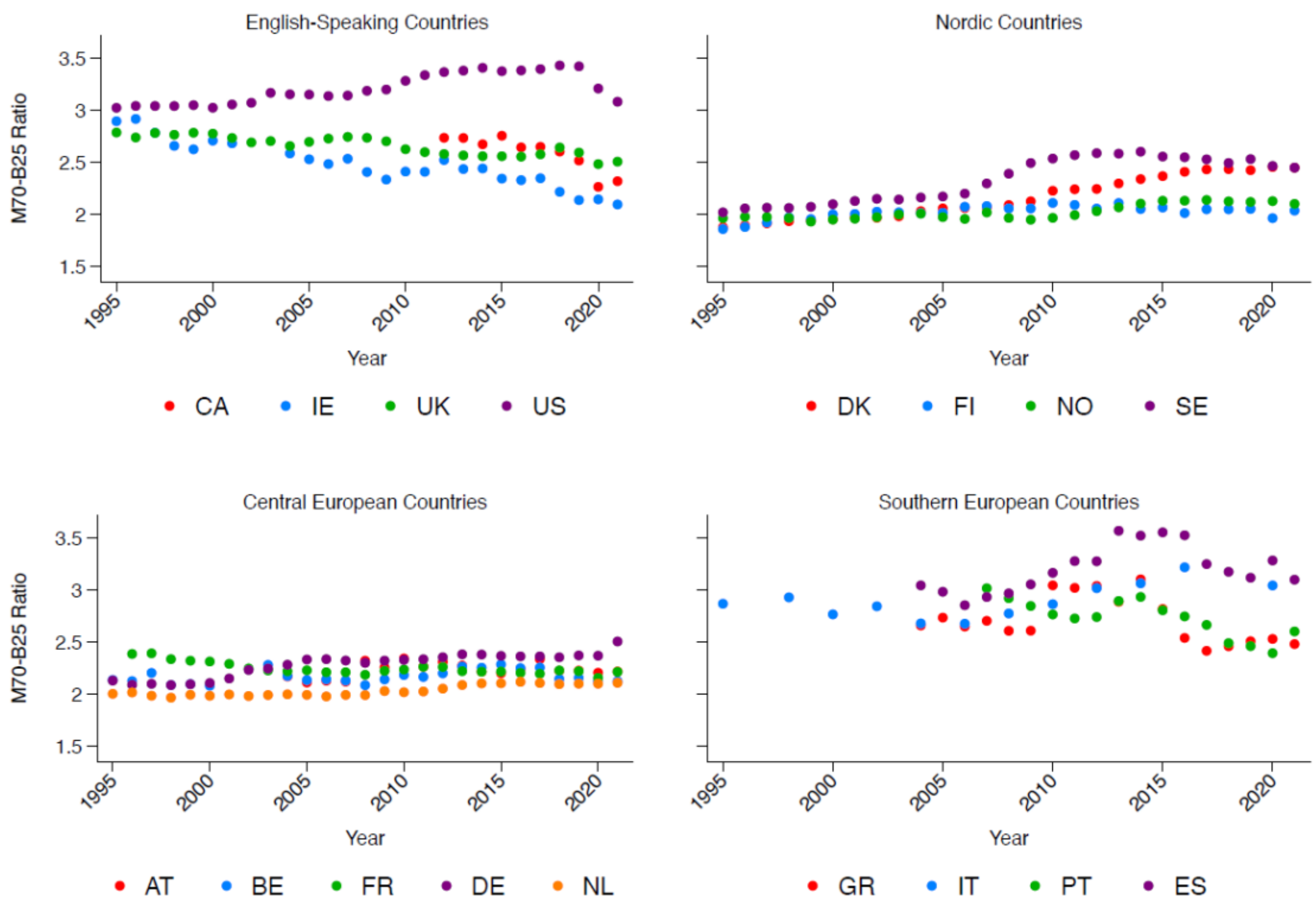


**Fig. S21.** Cross-Country Trends in Disposable Income Inequality among the Working-Age Population. Inequality is measured by the M70-B25 Ratio, which is the average income received by the middle 70% of households divided by the average income received by the bottom 25% of households. Disposable income is the sum of market income from labor earnings, ordinary capital income, nongovernmental transfers, and governmental cash and near-cash transfer income, less tax payments inclusive of refundable credits. Income includes contributions from household members of all ages, and is equivalized using the Modified OECD scale. The sample used in the figure is the working-age population defined as those aged 25-60. Countries are grouped into English-speaking nations of Canada (CA), Ireland (IE), United Kingdom (UK), and United States (US); Nordic countries of Denmark (DK), Finland (FI), Norway (NO), and Sweden (SE); Central European countries of Austria (AT), Belgium (BE), France (FR), Germany (DE), and The Netherlands (NL); and Southern European nations of Greece (GR), Italy (IT), Portugal (PT), and Spain (ES).

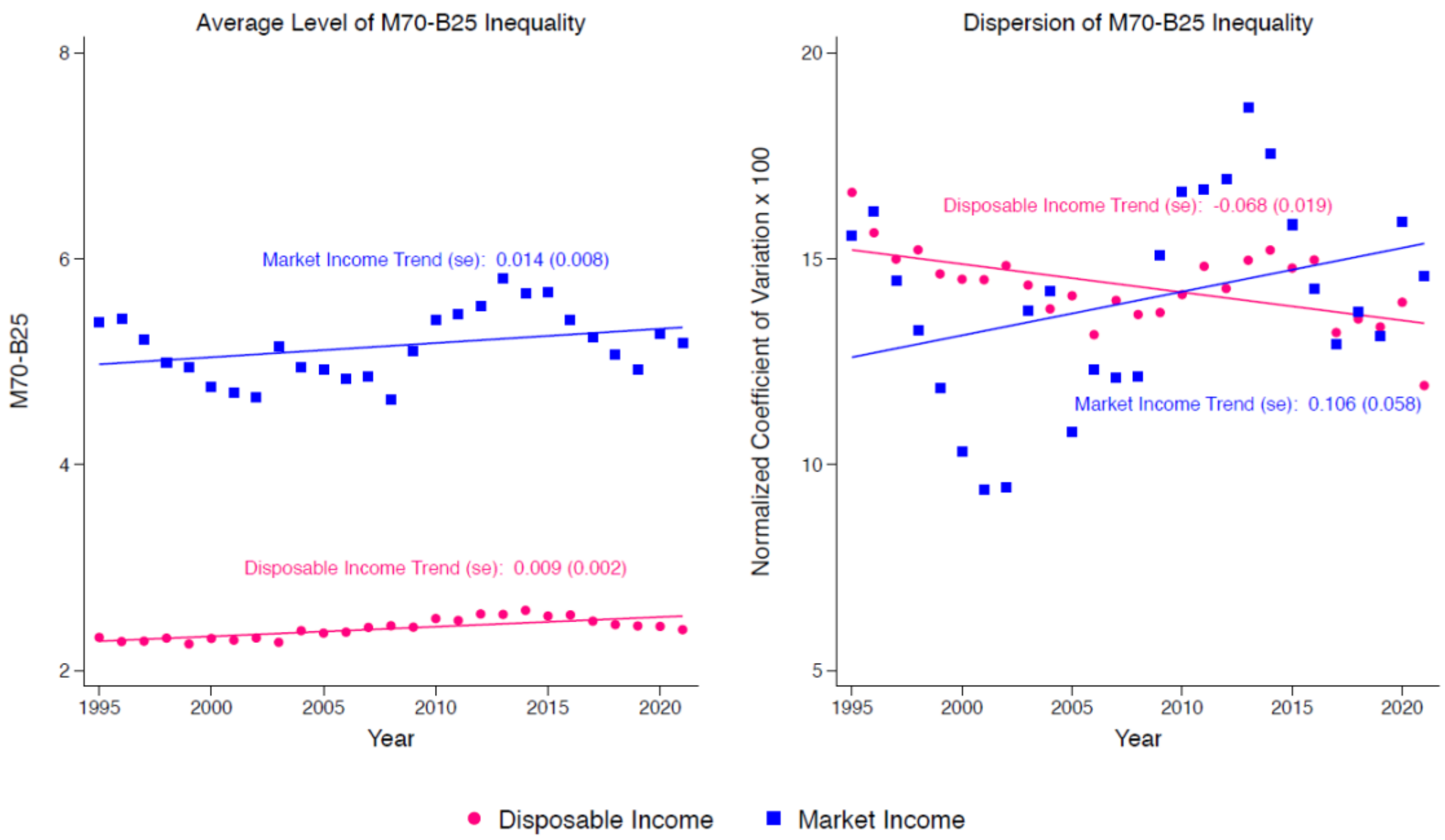


**Fig. S22.** Average Level of and Convergence in Cross-Country Disposable and Market Income Inequality among the Working-Age Population. Inequality is measured by the M70-B25 Ratio, which is the average income received by the middle 70% of households divided by the average income received by the bottom 25% of households. Dispersion is measured by the normalized coefficient of variation of inequality ($\boldsymbol{NCV} = \frac{\boldsymbol{CV}}{\boldsymbol{1+CV}}$) times 100, where CV is the ratio of the standard deviation to the mean, $\boldsymbol{CV} = {}^{\boldsymbol{\sigma}}/_{\boldsymbol{\mu}}$. The test of convergence is represented by the coefficient and associated standard error (se) on the linear trend line through the NCV in the figure. Market income is the sum of labor earnings, ordinary capital income, and nongovernmental transfers. Disposable income is market income plus cash and near-cash governmental transfers less tax payments inclusive of refundable credits. Income includes contributions from household members of all ages, and is equivalized using the Modified OECD scale. Working-age population is defined as those aged 25-60.

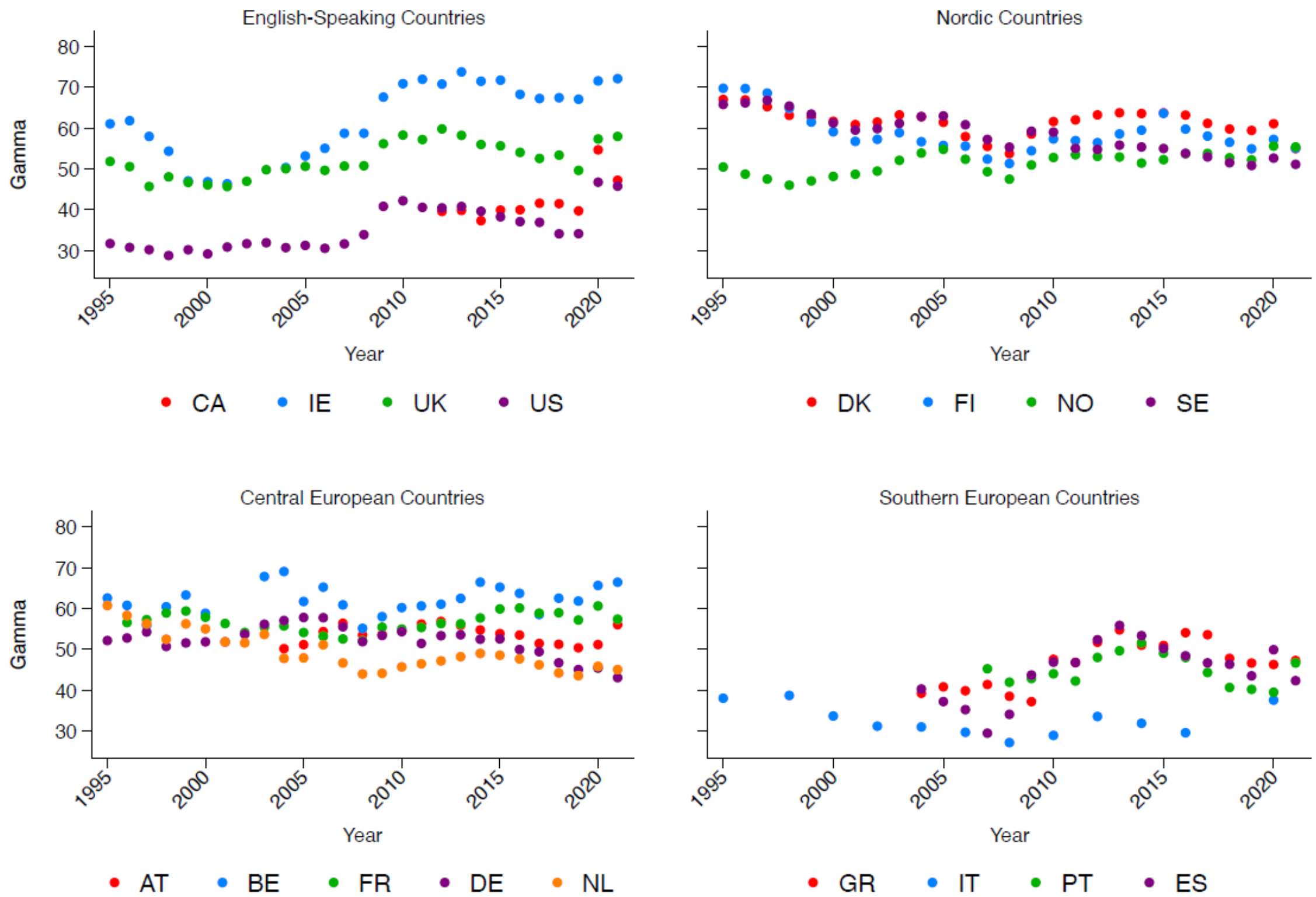


**Fig. S23.** Cross-Country Trends in Redistribution from Market Income to Disposable Income for Working-Age Population. Inequality is measured by the M70-B25 Ratio, which is the average income received by the middle 70% of households divided by the average income received by the bottom 25% of households. Gamma is 100 times the relative reduction in market income inequality to disposable income inequality from redistribution defined as $\hat{\gamma}_{j,t}^{M70-B25} = \mathbf{100} * (\mathbf{1} - \frac{\hat{I}_{j,t}^{Disp\ M70-B25}}{\hat{I}_{j,t}^{Market\ M70-B25}})$. Market income is the sum of labor earnings, ordinary capital income, and nongovernmental transfers. Disposable income is market income plus cash and near-cash governmental transfers less tax payments inclusive of refundable credits. Income includes contributions from household members of all ages, and is equivalized using the Modified OECD scale. Working-age population is defined as those aged 25-60. Countries are grouped into English-speaking nations of Canada (CA), Ireland (IE), United Kingdom (UK), and United States (US); Nordic countries of Denmark (DK), Finland (FI), Norway (NO), and Sweden (SE); Central European countries of Austria (AT), Belgium (BE), France (FR), Germany (DE), and The Netherlands (NL); and Southern European nations of Greece (GR), Italy (IT), Portugal (PT), and Spain (ES).

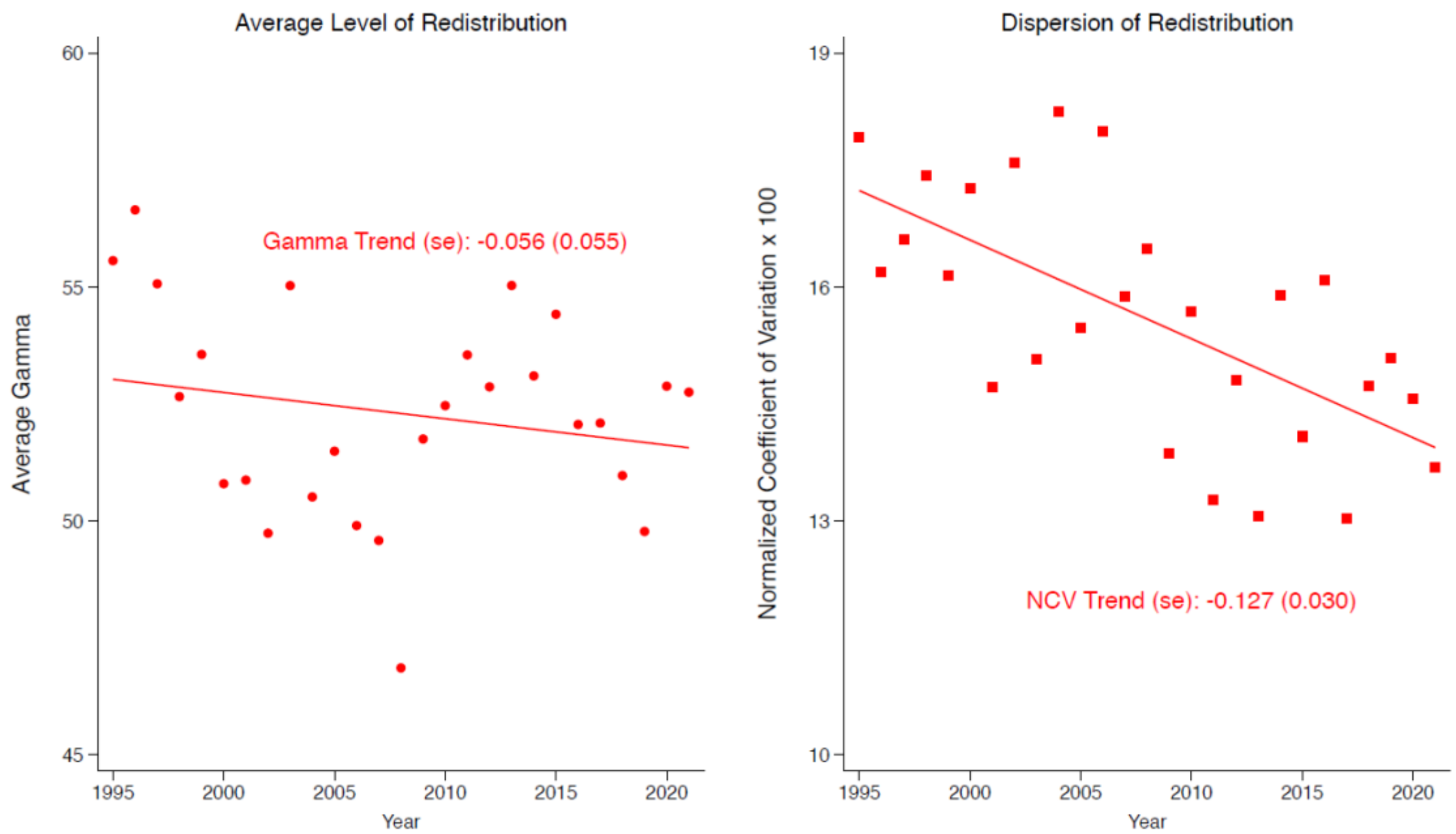


**Fig. S24.** Average Level of and Convergence in Cross-Country Redistribution from Market Income to Disposable Income among the Working-Age Population. Average Gamma is the cross-country average of 100 times the relative reduction in market income inequality to disposable income inequality from redistribution. Inequality is measured by the M70-B25 Ratio, which is the average income received by the middle 70% of households divided by the average income received by the bottom 25% of households. Dispersion is measured by the normalized coefficient of variation of gamma ($NCV = \frac{CV}{1+CV}$) times 100, where CV is the ratio of the standard deviation to the mean, $CV = {}^{\sigma}/_{\mu}$. The test of convergence is represented by the coefficient and associated standard error (se) on the linear trend line through the NCV in the figure. Market income is the sum of labor earnings, ordinary capital income, and nongovernmental transfers. Disposable income is market income plus cash and near-cash governmental transfers less tax payments inclusive of refundable credits. Income includes contributions from household members of all ages, and is equivalized using the Modified OECD scale. Working-age population is defined as those aged 25-60.

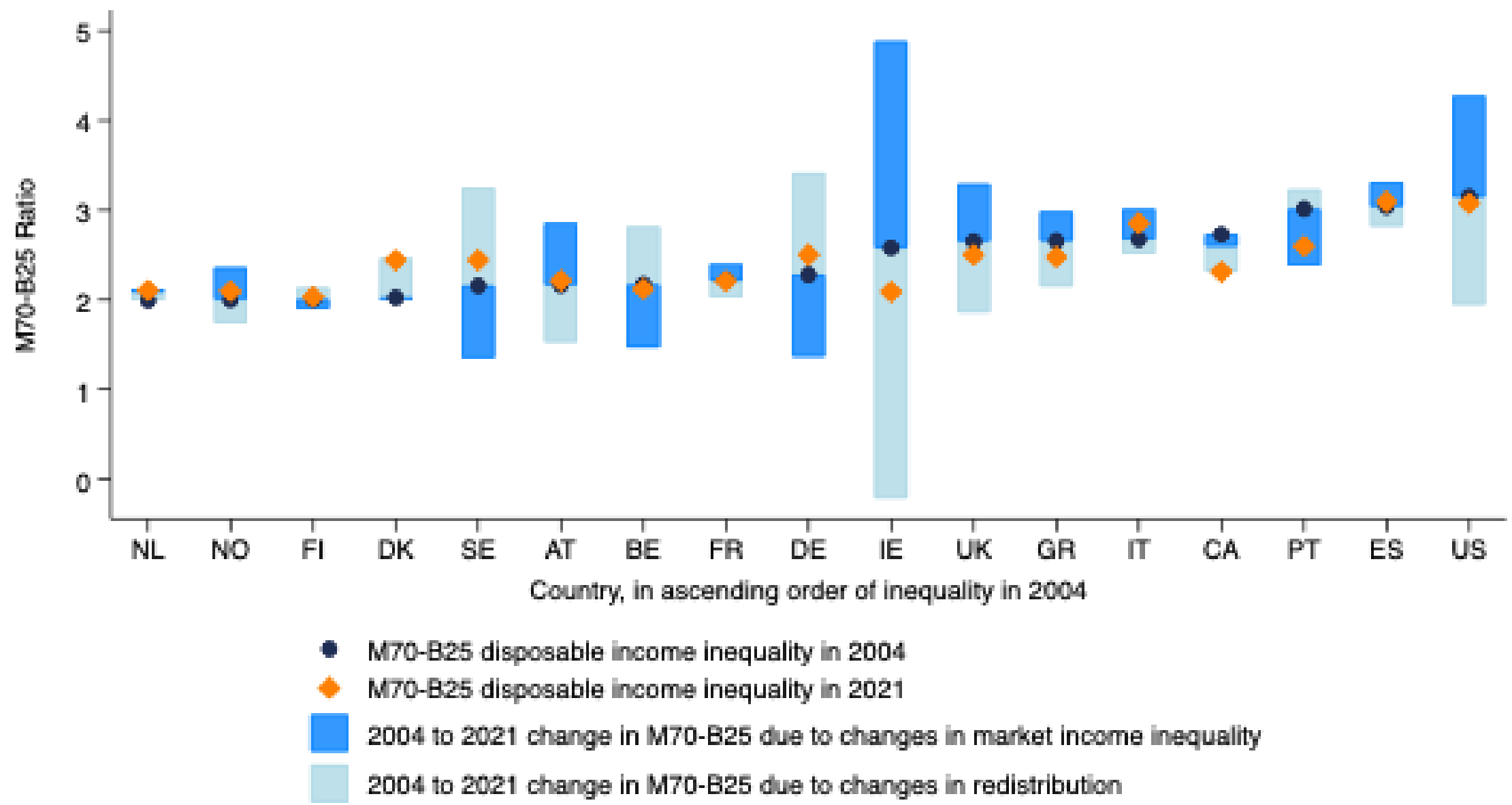


**Fig. S25.** Decomposition of the Change in Equivalized Household Disposable Income Inequality from 2004-2021 among the Working-Age Population. Inequality is measured by the M70-B25 Ratio, which is the average income received by the middle 70% of households divided by the average income received by the bottom 25% of households. Market income is the sum of labor earnings, ordinary capital income, and nongovernmental transfers, and the mid-blue color bar reflects the change in market income inequality from 2004-2021. Disposable income is market income plus cash and near-cash governmental transfers less tax payments inclusive of refundable credits. Redistribution is the change in income inequality in moving from market income inequality to disposable income inequality as reflected in the light-blue color bar. See equation (4) in *Materials and Methods* for additional details. Income includes contributions from household members of all ages, and is equivalized using the Modified OECD scale. Working-age population is defined as those aged 25-60. Countries are listed in ascending order of initial 2004 disposable income inequality as The Netherlands (NL), Norway (NO), Finland (FI), Denmark (DK), Sweden (SE), Austria (AT), Belgium (BE), France (FR), Germany (DE), Ireland (IE), United Kingdom (UK), Greece (GR), Italy (IT), Canada (CA), Portugal (PT), Spain (ES), and United States (US). For CA, income in 2004 is from 2012; for IT, income in 2021 is from 2022; and for PT, income in 2004 is from 2007.

**5. Alternative Methods of Data Censoring**

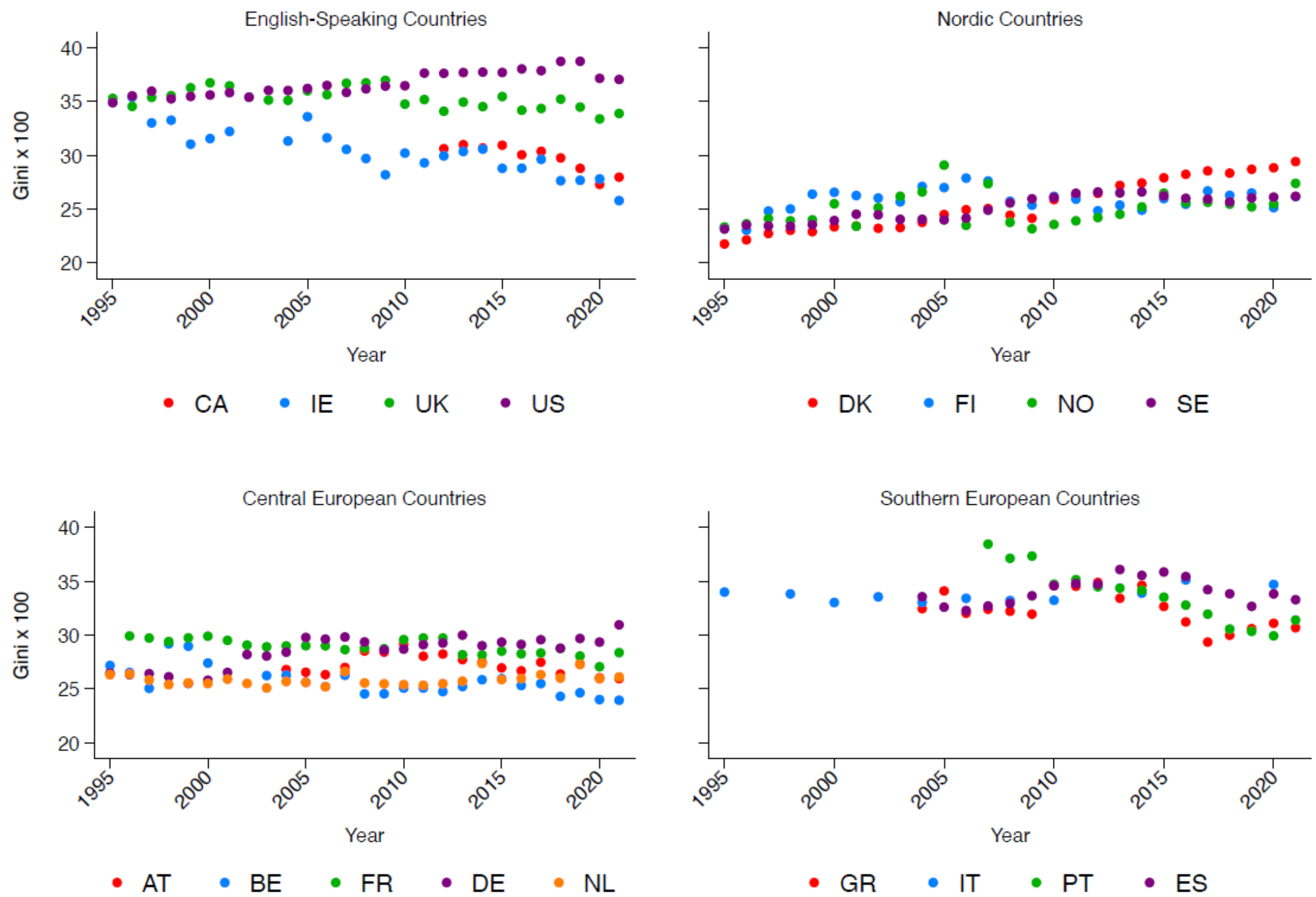


**Fig. S26.** Cross-Country Trends in Disposable Income Inequality among the Working-Age Population with Winsorizing at 0.1 and 99.9 Percentiles. Inequality is measured by the Gini coefficient times 100. Disposable income is the sum of market income from labor earnings, ordinary capital income, nongovernmental transfers, and governmental cash and near-cash transfer income, less tax payments inclusive of refundable credits. Income includes contributions from household members of all ages, and is equivalized using the Modified OECD scale. The sample used in the figure is the working-age population defined as those aged 25-60. Countries are grouped into English-speaking nations of Canada (CA), Ireland (IE), United Kingdom (UK), and United States (US); Nordic countries of Denmark (DK), Finland (FI), Norway (NO), and Sweden (SE); Central European countries of Austria (AT), Belgium (BE), France (FR), Germany (DE), and The Netherlands (NL); and Southern European nations of Greece (GR), Italy (IT), Portugal (PT), and Spain (ES).

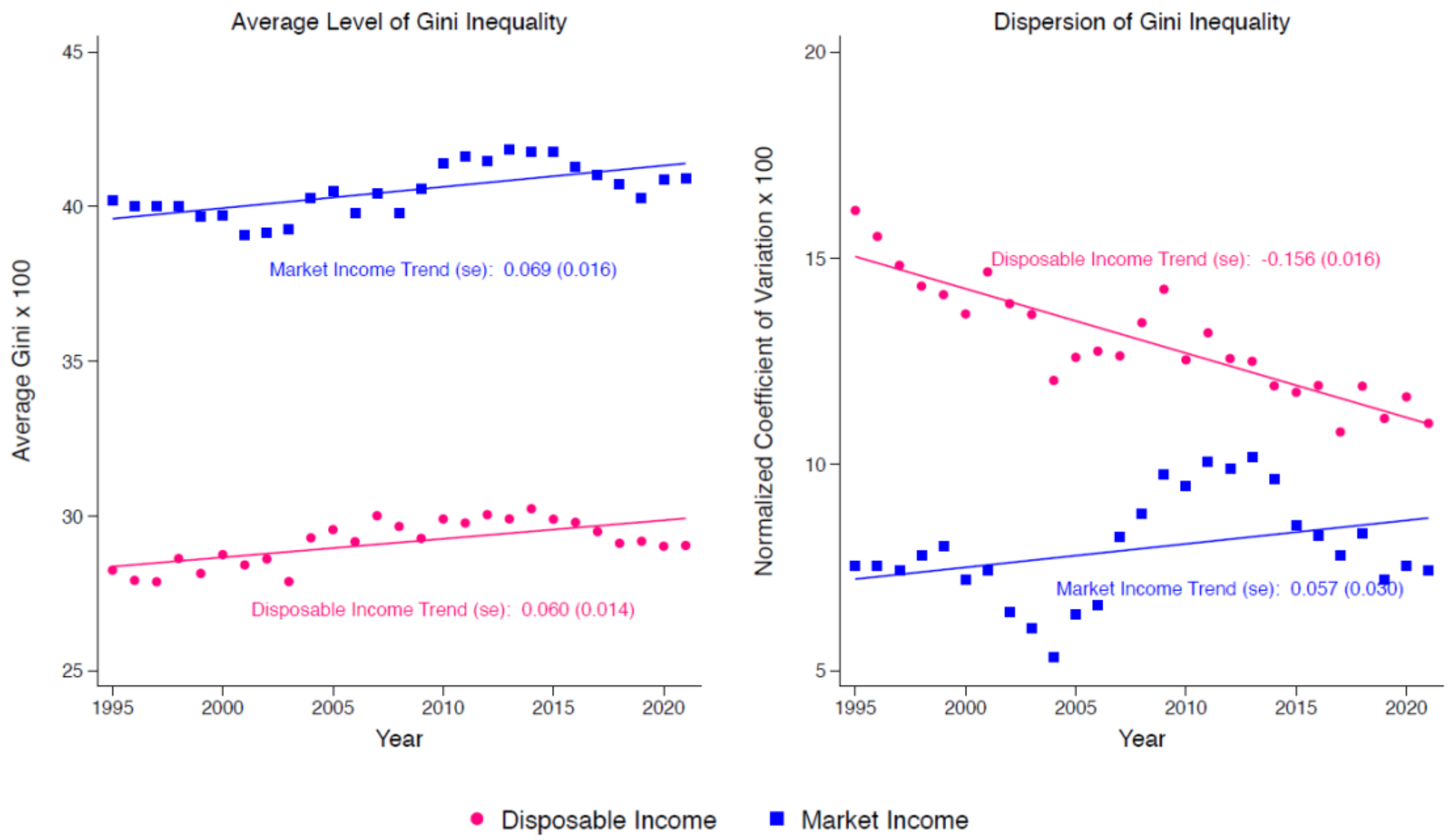


**Fig. S27.** Average Level of and Convergence in Cross-Country Disposable and Market Income Inequality among the Working-Age Population with Winsorizing at the 0.1 and 99.9 Percentiles. Inequality is measured by the Gini coefficient times 100. Dispersion is measured by the normalized coefficient of variation of inequality ($NCV = \frac{CV}{1+CV}$) times 100, where CV is the ratio of the standard deviation to the mean, $CV = \sigma/\mu$. The test of convergence is represented by the coefficient and associated standard error (se) on the linear trend line through the NCV in the figure. Market income is the sum of labor earnings, ordinary capital income, and nongovernmental transfers. Disposable income is market income plus cash and near-cash governmental transfers less tax payments inclusive of refundable credits. Income includes contributions from household members of all ages, and is equivalized using the Modified OECD scale. Working-age population is defined as those aged 25-60.

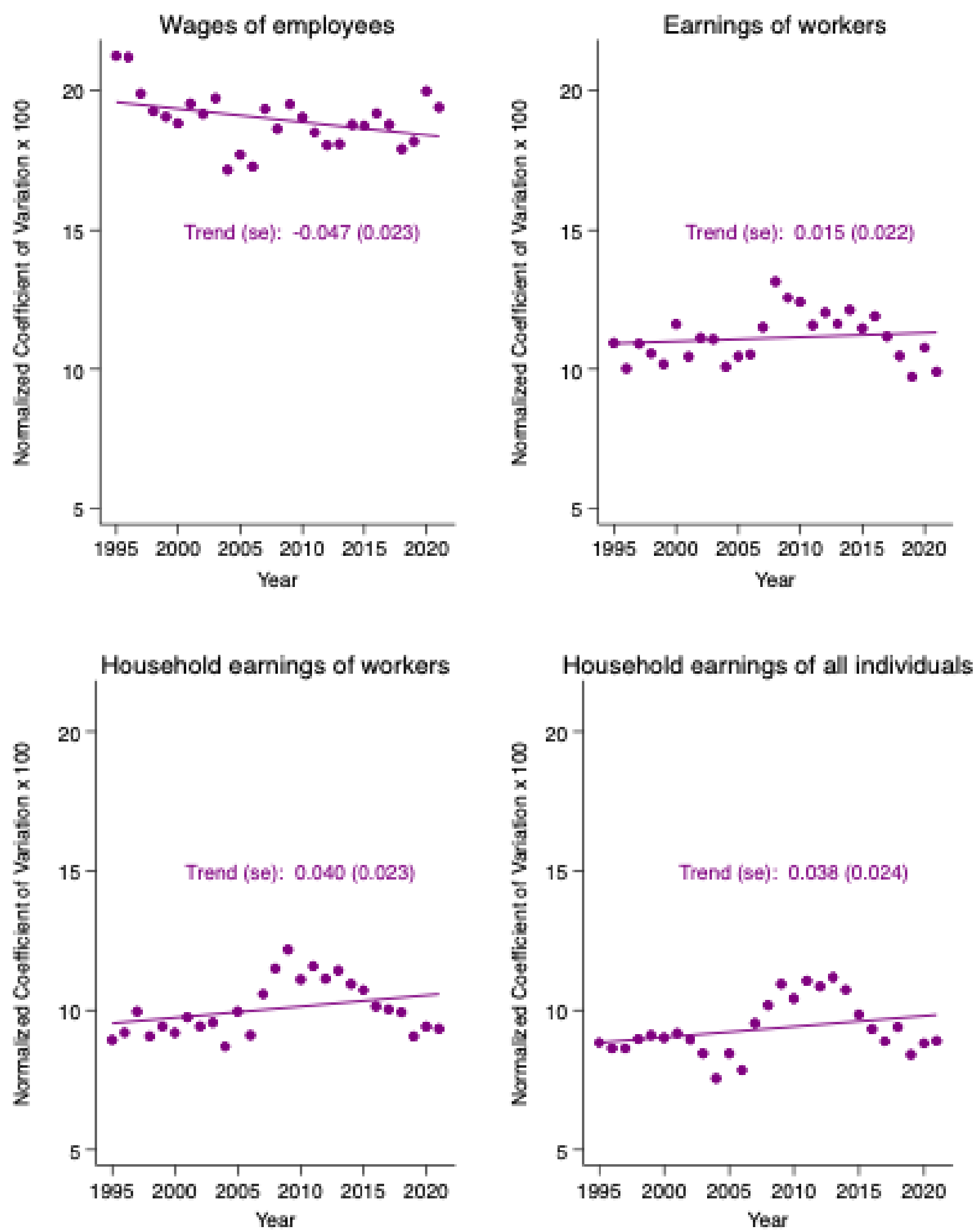


**Fig. S28.** Tests of Convergence in Cross-Country Labor-Market Inequalities among Working-Age Population with Winsorizing at the 0.1 and 99.9 Percentiles. Inequality is measured by the Gini coefficient times 100. Dispersion is measured by the normalized coefficient of variation of inequality ($\boldsymbol{NCV} = \frac{\boldsymbol{CV}}{1+\boldsymbol{CV}}$) times 100, where CV is the ratio of the standard deviation to the mean, $\boldsymbol{CV} = {}^{\sigma}/_{\mu}$. The test of convergence is represented by the coefficient and associated standard error (se) on the linear trend line through the NCV in the figure. Wages of employees are average hourly earnings for employees. Earnings of workers are annual earnings of employees and self-employed workers. Household earnings of workers add up annual earnings of all persons who are employees or self-employed workers Household earnings of all individuals include non-working households. Household earnings are equivalized using the Modified OECD scale. Working-age population is defined as those aged 25-60.

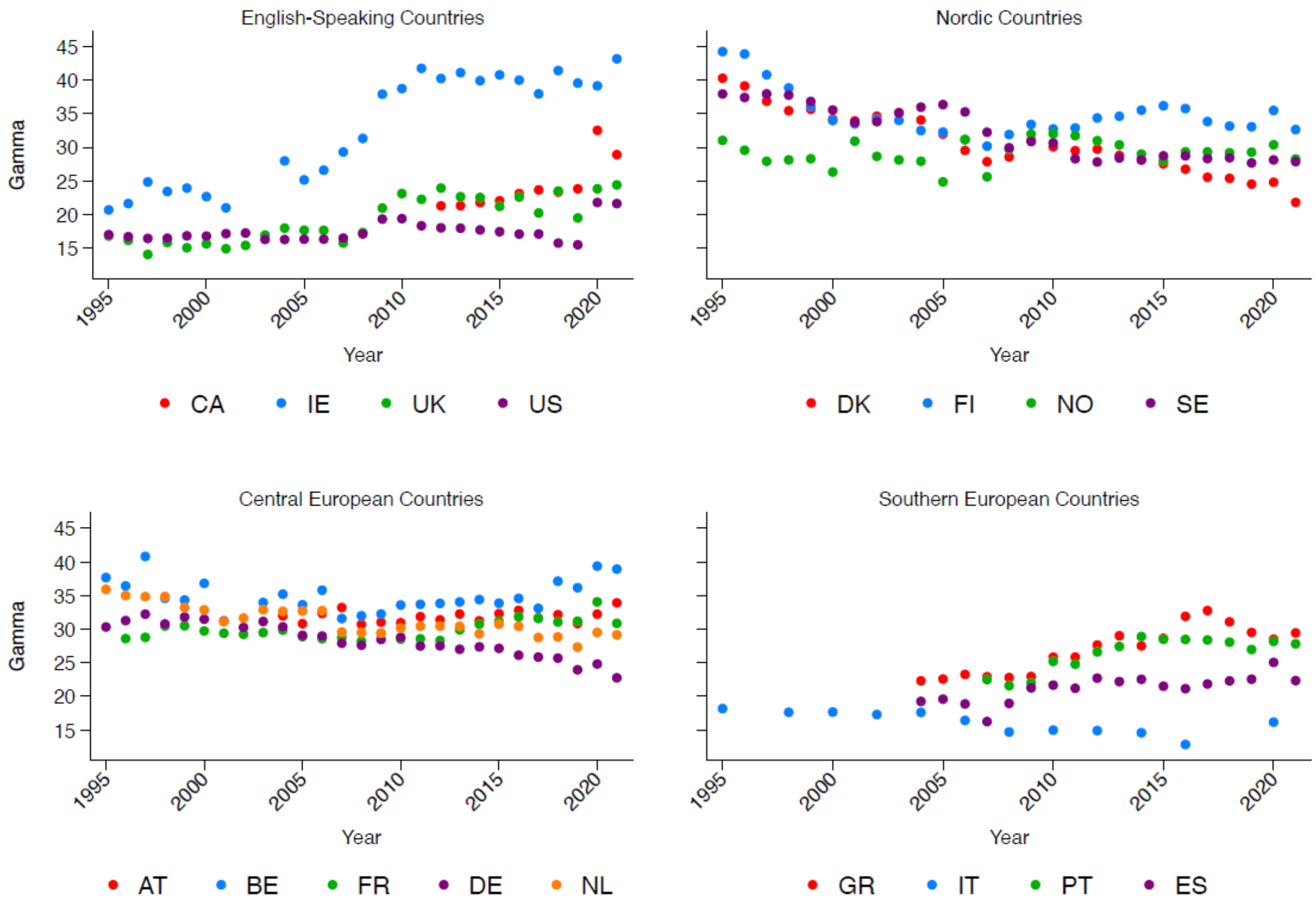


**Fig. S29.** Cross-Country Trends in Redistribution from Market Income to Disposable Income for Working-Age Population with Winsorizing at the 0.1 and 99.9 Percentiles. Inequality is measured by the Gini coefficient. Gamma is 100 times the relative reduction in market income inequality to disposable income inequality defined as $\hat{\gamma}_{j,t}^{Gini} = 100 * (1 - \frac{\hat{I}_{j,t}^{Disp\ Gini}}{\hat{I}_{j,t}^{Market\ Gini}})$. Market income is the sum of labor earnings, ordinary capital income, and nongovernmental transfers. Disposable income is market income plus cash and near-cash governmental transfers less tax payments inclusive of refundable credits. Income includes contributions from household members of all ages, and is equivalized using the Modified OECD scale. Working-age population is defined as those aged 25-60. Countries are grouped into English-speaking nations of Canada (CA), Ireland (IE), United Kingdom (UK), and United States (US); Nordic countries of Denmark (DK), Finland (FI), Norway (NO), and Sweden (SE); Central European countries of Austria (AT), Belgium (BE), France (FR), Germany (DE), and The Netherlands (NL); and Southern European nations of Greece (GR), Italy (IT), Portugal (PT), and Spain (ES).

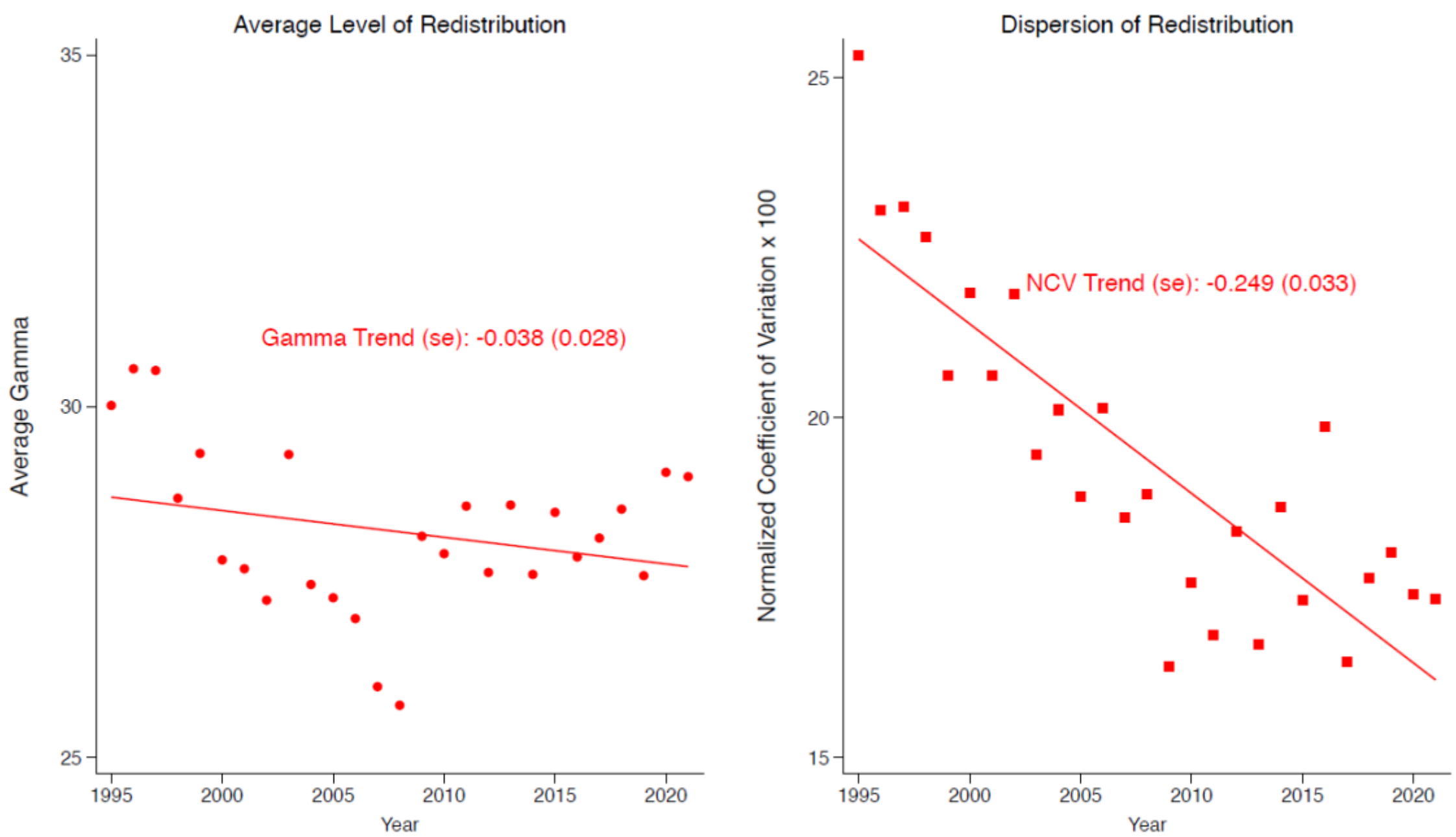


**Fig. S30.** Average Level of and Convergence in Cross-Country Redistribution from Market Income to Disposable Income among the Working-Age Population with Winsorizing at the 0.1 and 99.9 Percentiles. Average Gamma is the cross-country average of 100 times the relative reduction in market income inequality to disposable income inequality defined as $\hat{\gamma}_{j,t}^{Gini} = \mathbf{100} * (\mathbf{1} - \frac{\hat{I}_{j,t}^{Disp\ Gini}}{\hat{I}_{j,t}^{Market\ Gini}})$. Inequality is measured by the Gini coefficient. Dispersion is measured by the normalized coefficient of variation of gamma ($\boldsymbol{NCV} = \frac{CV}{1+CV}$) times 100, where CV is the ratio of the standard deviation to the mean, $\boldsymbol{CV} = {}^{\sigma}/_{\mu}$. The test of convergence is represented by the coefficient and associated standard error (se) on the linear trend line through the NCV in the figure. Market income is the sum of labor earnings, ordinary capital income, and nongovernmental transfers. Disposable income is market income plus cash and near-cash governmental transfers less tax payments inclusive of refundable credits. Income includes contributions from household members of all ages, and is equivalized using the Modified OECD scale. Working-age population is defined as those aged 25-60.

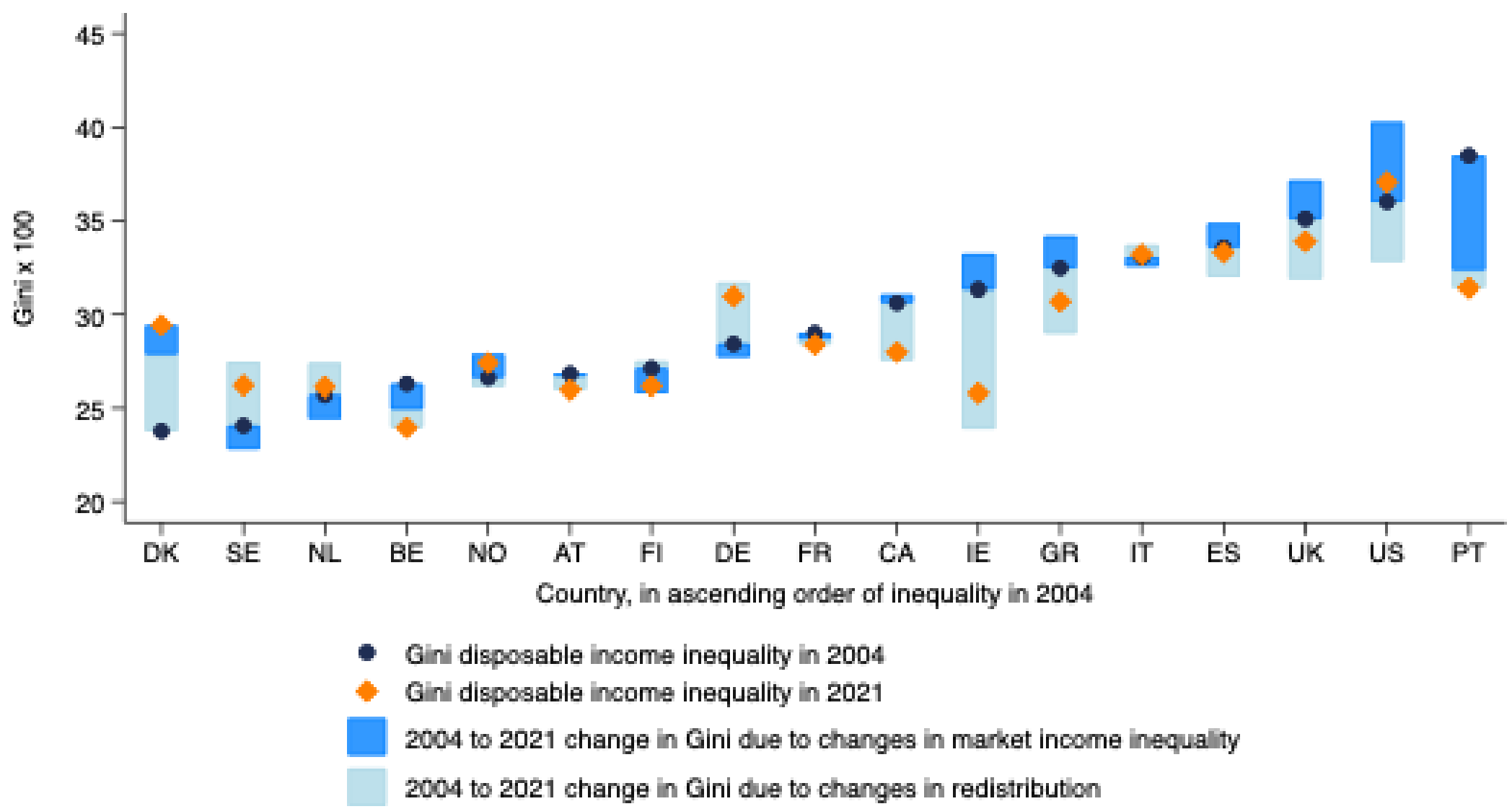


**Fig. S31.** Decomposition of the Change in Disposable Income Inequality from 2004-2021 among the Working-Age Population with Winsorizing at the 0.1 and 99.9 Percentiles. Inequality is measured by the Gini coefficient times 100. Market income is the sum of labor earnings, ordinary capital income, and nongovernmental transfers, and the mid-blue color bar reflects the change in market income inequality from 2004-2021. Disposable income is market income plus cash and near-cash governmental transfers less tax payments inclusive of refundable credits. Redistribution is the change in income inequality in moving from market income inequality to disposable income inequality as reflected in the light-blue color bar. See equation (4) in *Materials and Methods* for additional details. Income includes contributions from household members of all ages, and is equivalized using the Modified OECD scale. Working-age population is defined as those aged 25-60. Countries are listed in ascending order of initial 2004 disposable income inequality as Denmark (DK), Sweden (SE), The Netherlands (NL), Belgium (BE), Norway (NO), Austria (AT), Finland (FI), Germany (DE), France (FR), Canada (CA), Ireland (IE), Greece (GR), Italy (IT), Spain (ES), United Kingdom (UK), United States (US), and Portugal (PT). For CA, income in 2004 is from 2012; for IT, income in 2021 is from 2022; and for PT, income in 2004 is from 2007.

## 6. Balanced Panel of Countries

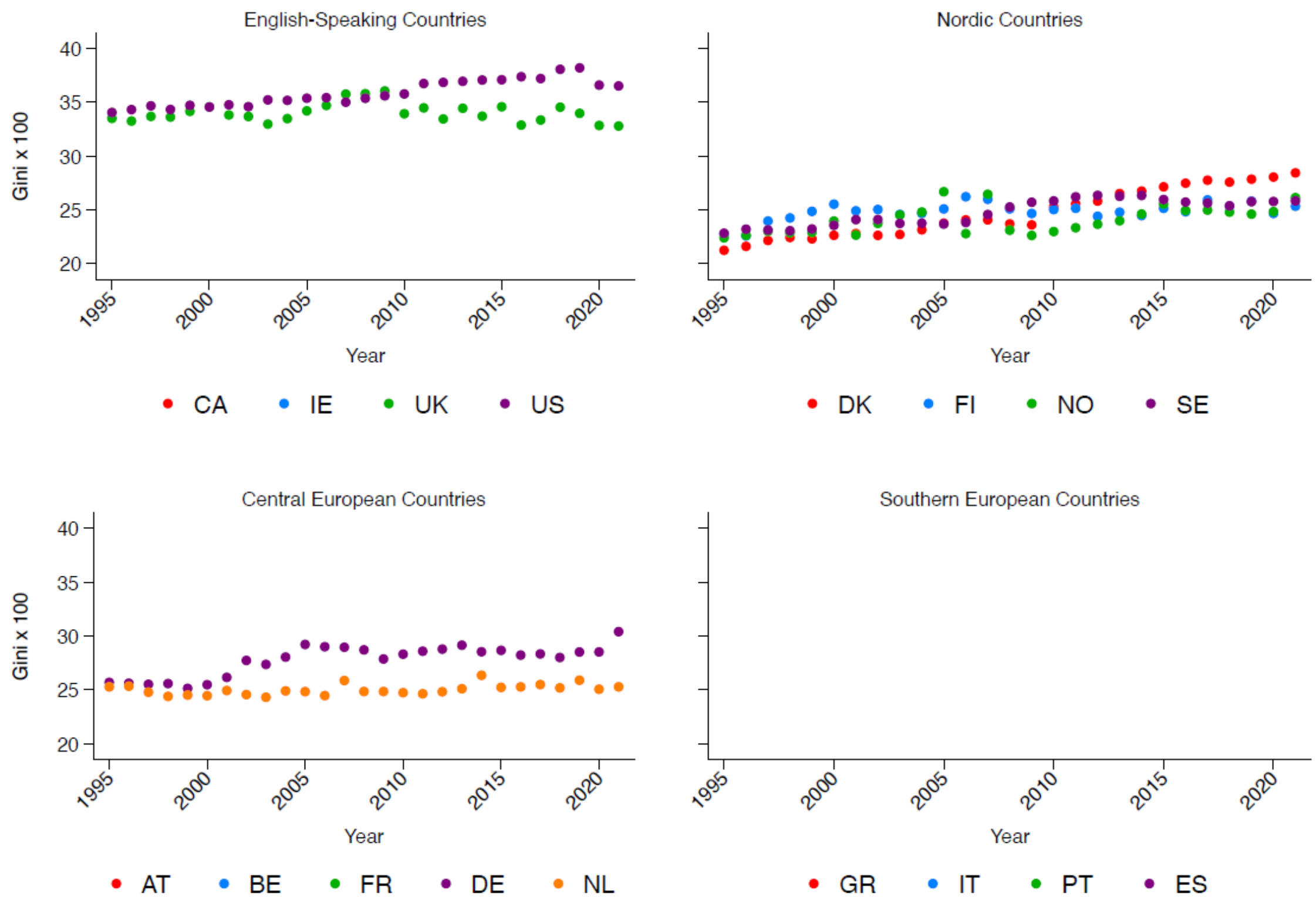


**Fig. S32.** Cross-Country Trends in Disposable Income Inequality among the Working-Age Population with a Balanced Panel of 8 Countries, 1995-2021. Inequality is measured by the Gini coefficient times 100. Disposable income is the sum of market income from labor earnings, ordinary capital income, nongovernmental transfers, and governmental cash and near-cash transfer income, less tax payments inclusive of refundable credits. Income includes contributions from household members of all ages, and is equivalized using the Modified OECD scale. The sample used in the figure is the working-age population defined as those aged 25-60. Countries are grouped into English-speaking nations of Canada (CA), Ireland (IE), United Kingdom (UK), and United States (US); Nordic countries of Denmark (DK), Finland (FI), Norway (NO), and Sweden (SE); Central European countries of Austria (AT), Belgium (BE), France (FR), Germany (DE), and The Netherlands (NL); and Southern European nations of Greece (GR), Italy (IT), Portugal (PT), and Spain (ES).

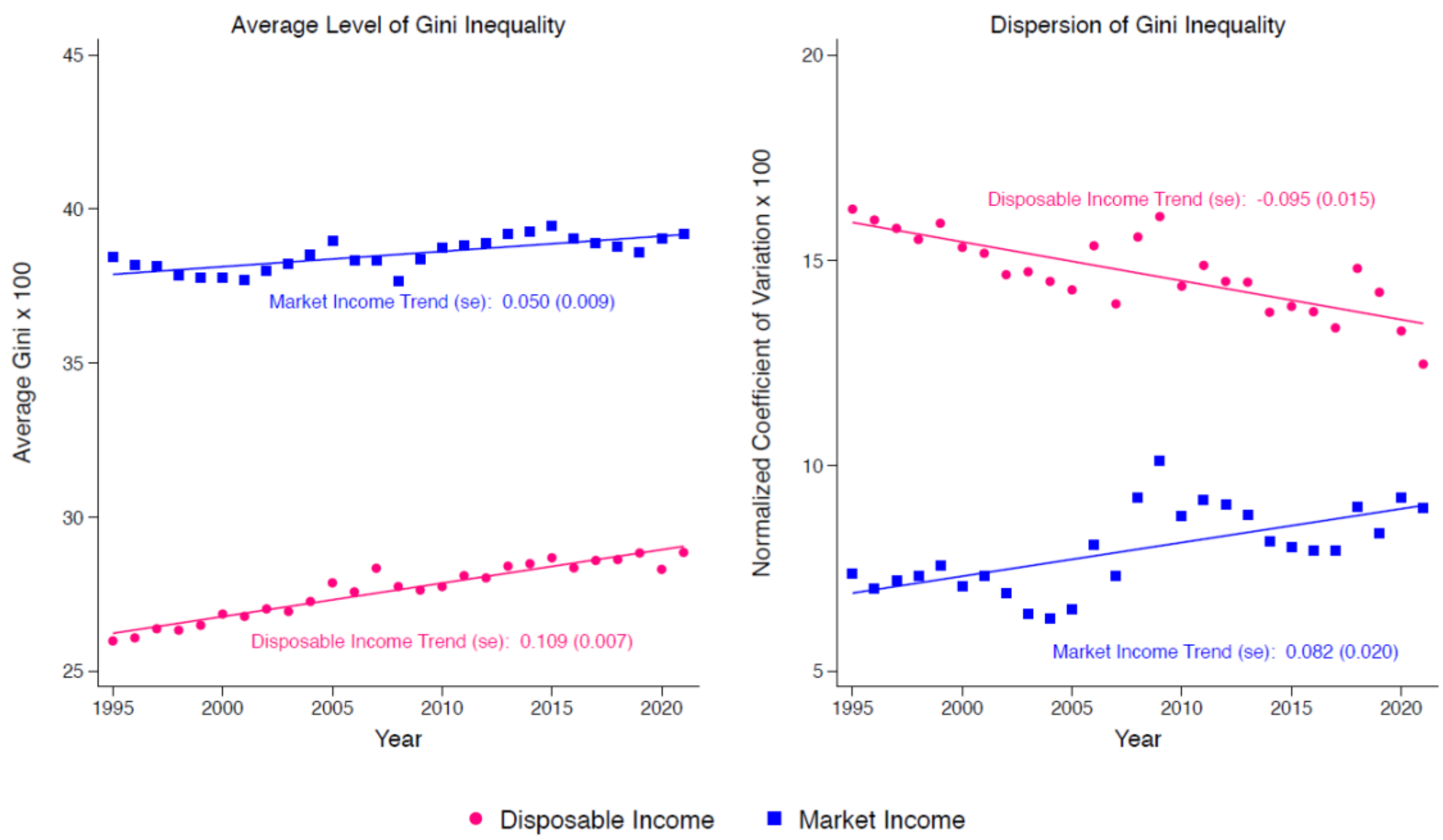


**Fig. S33.** Average Level of and Convergence in Cross-Country Disposable and Market Income Inequality among the Working-Age Population with a Balanced Panel of 8 Countries, 1995-2021. Inequality is measured by the Gini coefficient times 100. Dispersion is measured by the normalized coefficient of variation of inequality ($\boldsymbol{NCV} = \frac{\boldsymbol{CV}}{\boldsymbol{1+CV}}$) times 100, where CV is the ratio of the standard deviation to the mean, $\boldsymbol{CV} = {}^{\boldsymbol{\sigma}}/_{\boldsymbol{\mu}}$. The test of convergence is represented by the coefficient and associated standard error (se) on the linear trend line through the NCV in the figure. Market income is the sum of labor earnings, ordinary capital income, and nongovernmental transfers. Disposable income is market income plus cash and near-cash governmental transfers less tax payments inclusive of refundable credits. Income includes contributions from household members of all ages, and is equivalized using the Modified OECD scale. Working-age population is defined as those aged 25-60.

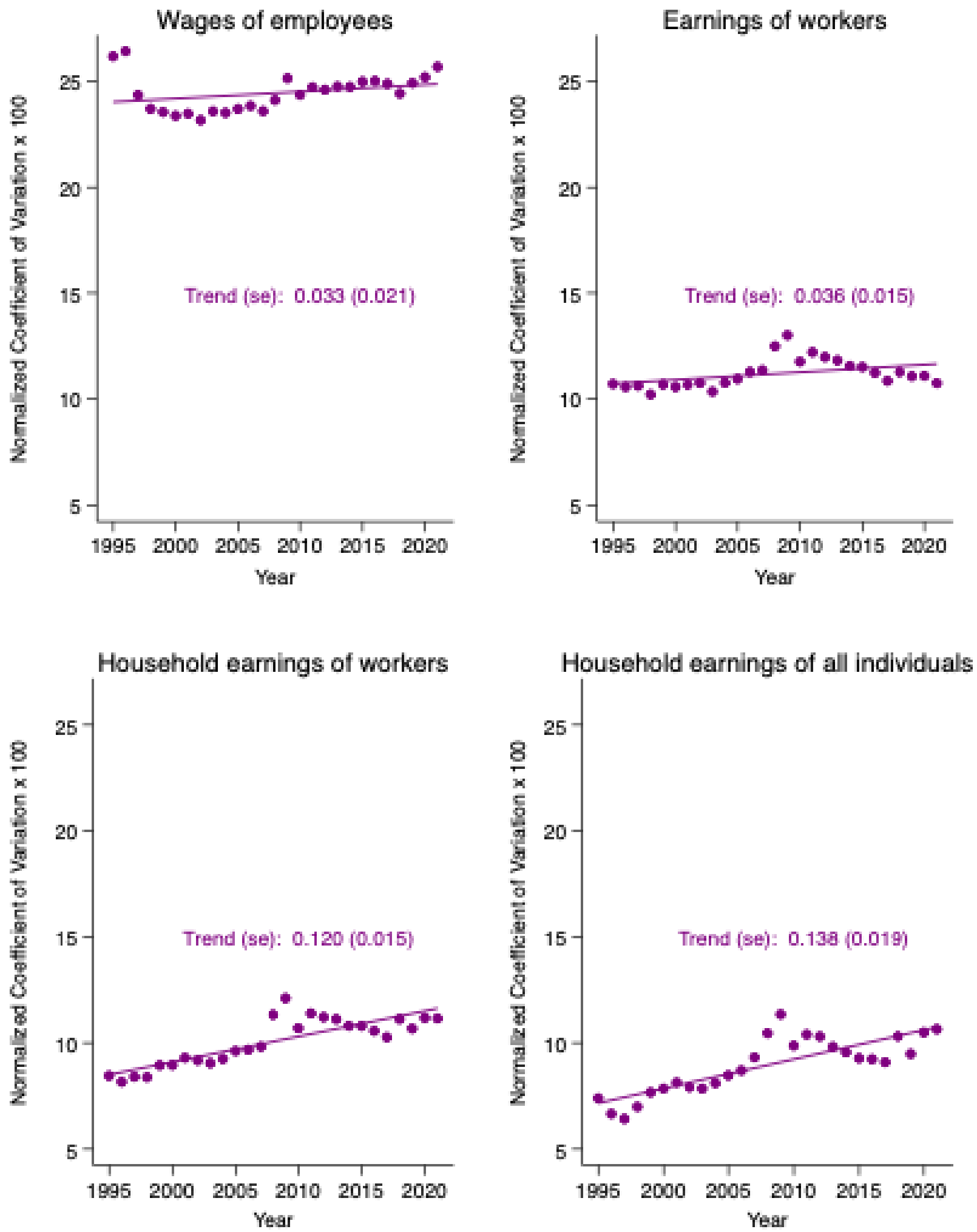


**Fig. S34.** Tests of Convergence in Cross-Country Labor-Market Inequalities among Working-Age Population with a Balanced Panel of 8 Countries, 1995-2021. Inequality is measured by the Gini coefficient times 100. Dispersion is measured by the normalized coefficient of variation of inequality ($\boldsymbol{NCV} = \frac{\boldsymbol{CV}}{1+\boldsymbol{CV}}$) times 100, where CV is the ratio of the standard deviation to the mean, $\boldsymbol{CV} = {}^{\sigma}/_{\mu}$. The test of convergence is represented by the coefficient and associated standard error (se) on the linear trend line through the NCV in the figure. Wages of employees are average hourly earnings for employees. Earnings of workers are annual earnings of employees and self-employed workers. Household earnings of workers add up annual earnings of all persons who are employees or self-employed workers. Household earnings of all individuals include non-working households. Household earnings are equivalized using the Modified OECD scale. Working-age population is defined as those aged 25-60.

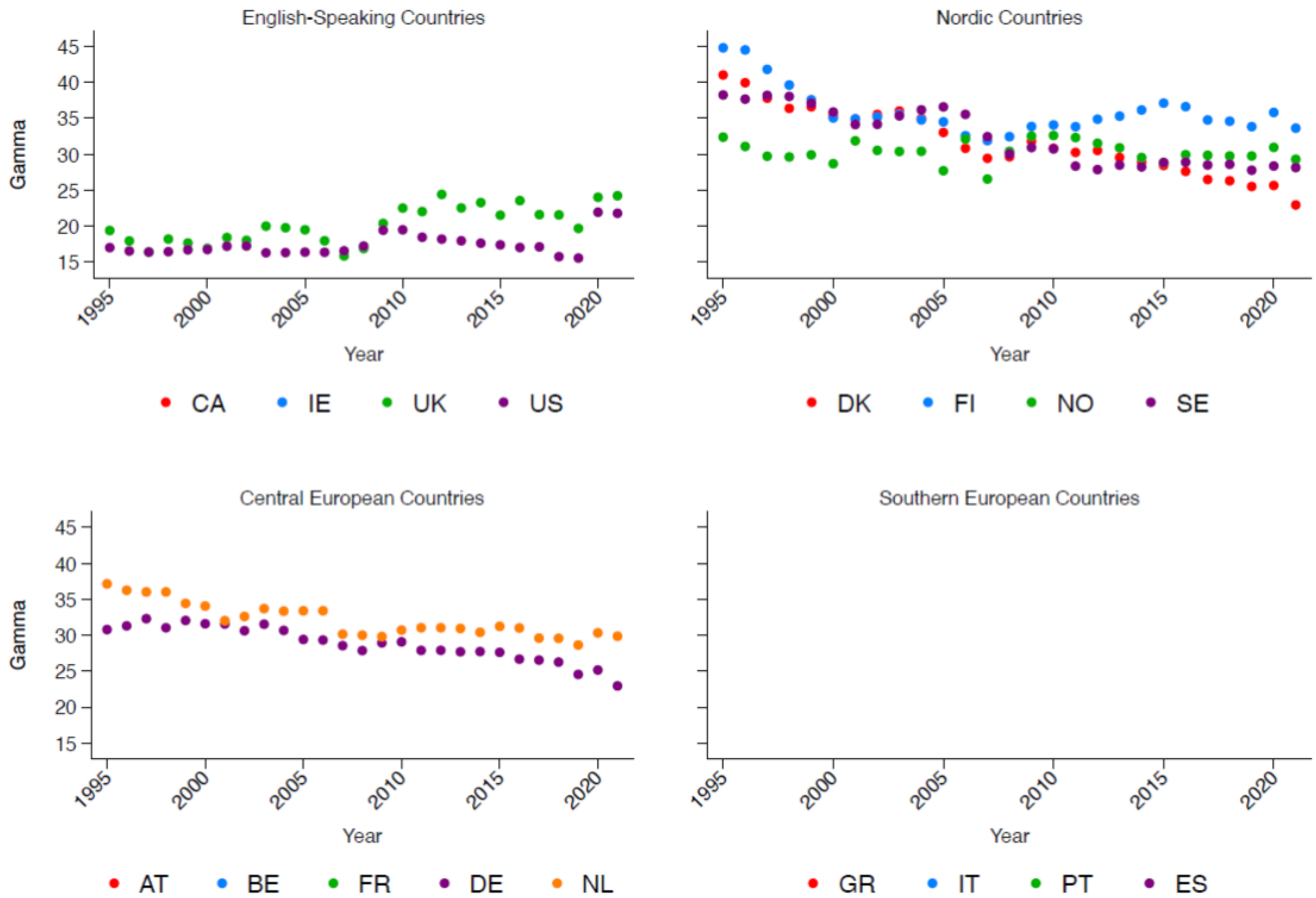


**Fig. S35.** Cross-Country Trends in Redistribution from Market Income to Disposable Income for Working-Age Population with a Balanced Panel of 8 Countries, 1995-2021. Inequality is measured by the Gini coefficient. Gamma is 100 times the relative reduction in market income inequality to disposable income inequality defined as $\hat{\gamma}_{j,t}^{Gini} = \mathbf{100} * (\mathbf{1} - \frac{\hat{I}_{j,t}^{Disp\ Gini}}{\hat{I}_{j,t}^{Market\ Gini}})$. Market income is the sum of labor earnings, ordinary capital income, and nongovernmental transfers. Disposable income is market income plus cash and near-cash governmental transfers less tax payments inclusive of refundable credits. Income includes contributions from household members of all ages, and is equivalized using the Modified OECD scale. Working-age population is defined as those aged 25-60. Countries are grouped into English-speaking nations of Canada (CA), Ireland (IE), United Kingdom (UK), and United States (US); Nordic countries of Denmark (DK), Finland (FI), Norway (NO), and Sweden (SE); Central European countries of Austria (AT), Belgium (BE), France (FR), Germany (DE), and The Netherlands (NL); and Southern European nations of Greece (GR), Italy (IT), Portugal (PT), and Spain (ES).

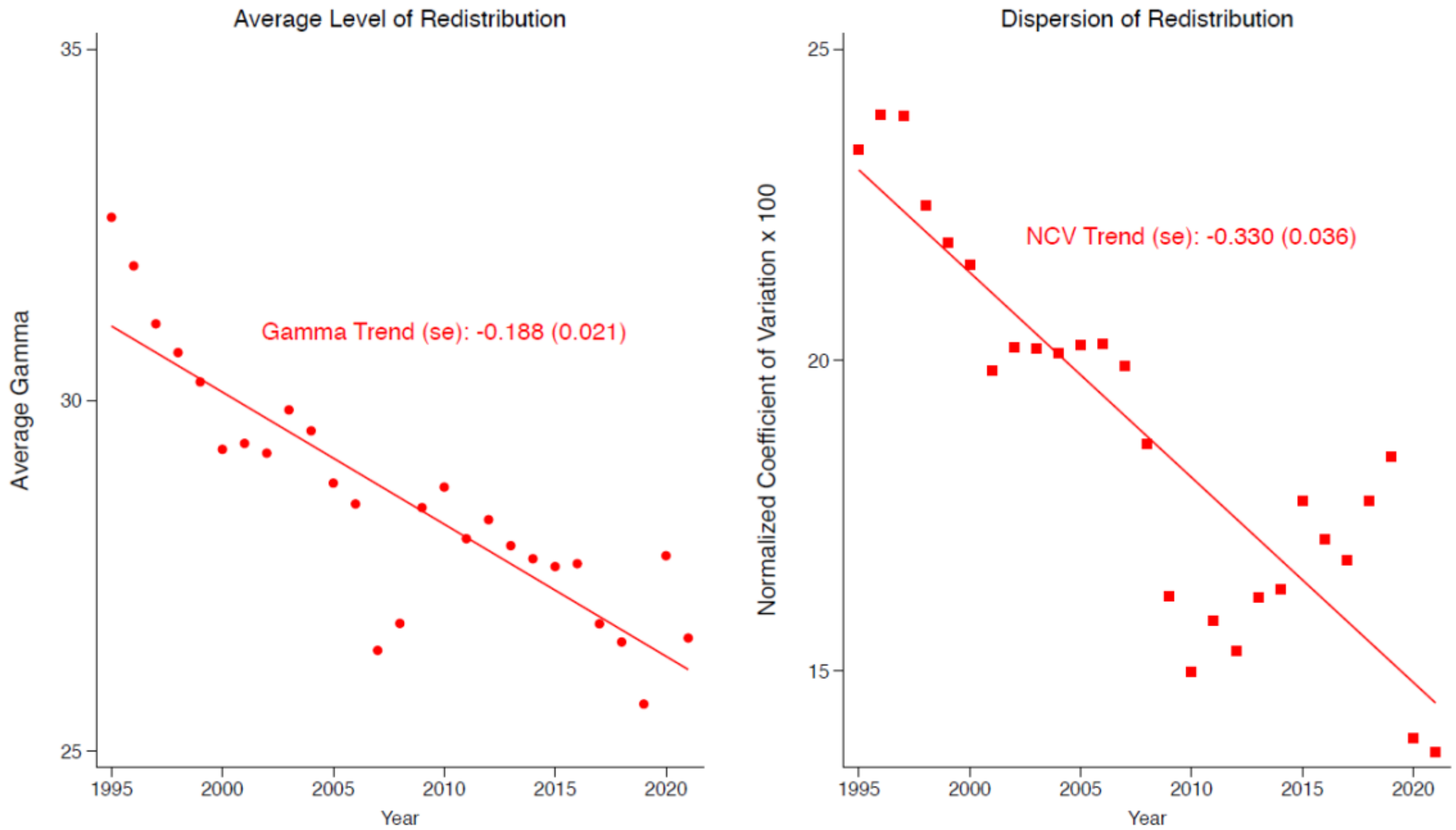


Fig. S36. Average Levels of and Convergence in Cross-Country Redistribution from Market Income to Disposable Income among the Working-Age Population with a Balanced Panel of 8 Countries, 1995-2021. Average Gamma is the cross-country average of 100 times the relative reduction in market income inequality to disposable income inequality defined as $\hat{\gamma}_{j,t}^{Gini} = 100 * (1 - \frac{\hat{I}_{j,t}^{Disp\ Gini}}{\hat{I}_{j,t}^{Market\ Gini}})$. Inequality is measured by the Gini coefficient. Dispersion is measured by the normalized coefficient of variation of gamma ($NCV = \frac{CV}{1+CV}$) times 100, where CV is the ratio of the standard deviation to the mean, $CV = {}^{\sigma}/_{\mu}$. The test of convergence is represented by the coefficient and associated standard error (se) on the linear trend line through the NCV in the figure. Market income is the sum of labor earnings, ordinary capital income, and nongovernmental transfers. Disposable income is market income plus cash and near-cash governmental transfers less tax payments inclusive of refundable credits. Income includes contributions from household members of all ages, and is equivalized using the Modified OECD scale. Working-age population is defined as those aged 25-60.

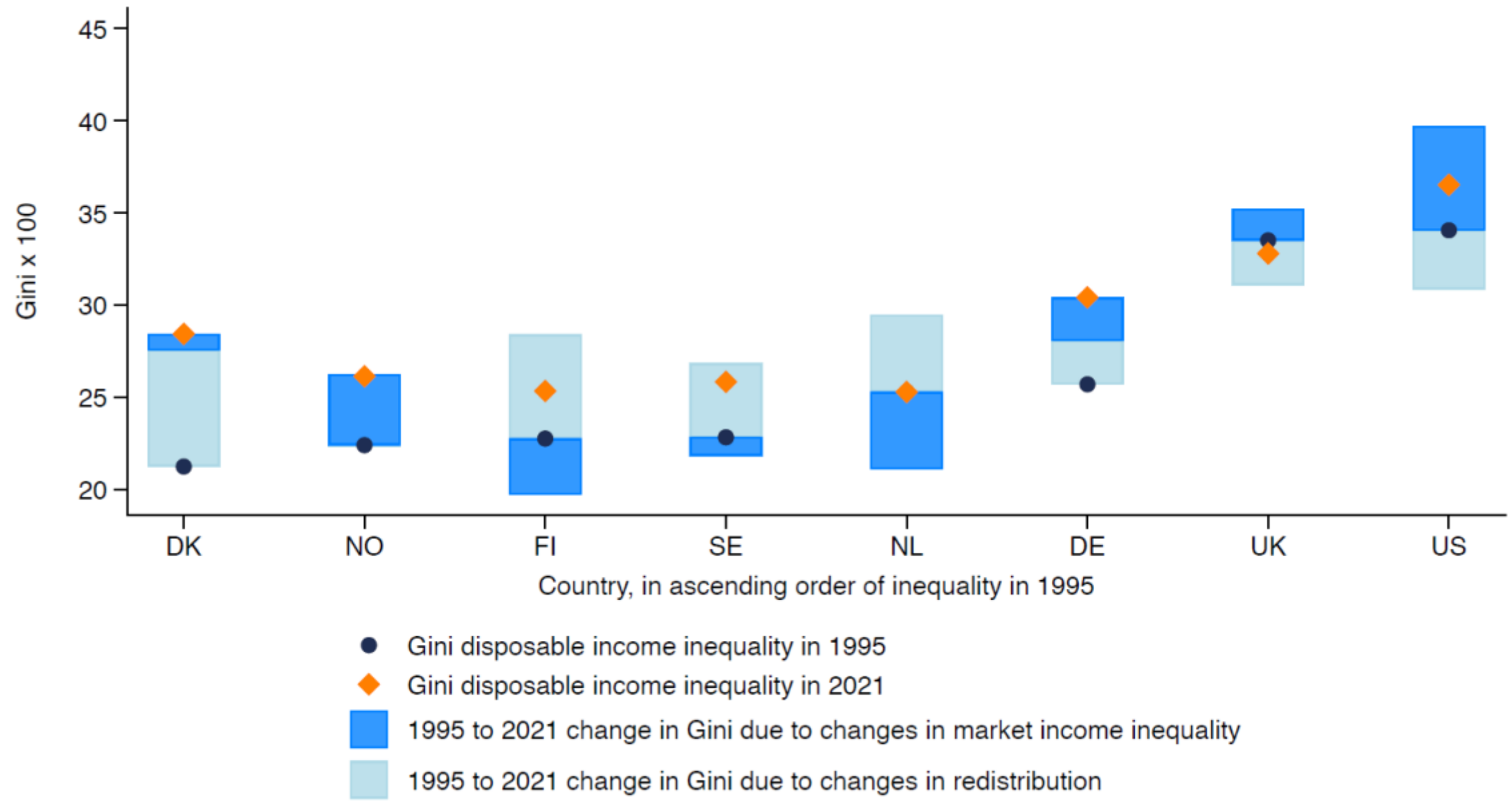


**Fig. S37.** Decomposition of the Change in Disposable Income Inequality from 1995-2021 among the Working-Age Population with a Balanced Panel of Countries. Inequality is measured by the Gini coefficient times 100. Market income is the sum of labor earnings, ordinary capital income, and nongovernmental transfers, and the mid-blue color bar reflects the change in market income inequality from 1995-2021. Disposable income is market income plus cash and near-cash governmental transfers less tax payments inclusive of refundable credits. Redistribution is the change in income inequality in moving from market income inequality to disposable income inequality as reflected in the light-blue color bar. See equation (4) in *Materials and Methods* for additional details. Income includes contributions from household members of all ages, and is equivalized using the Modified OECD scale. Working-age population is defined as those aged 25-60. Countries are listed in ascending order of initial 2004 disposable income inequality as Denmark (DK), Norway (NO), Finland (FI), Sweden (SE), The Netherlands (NL), Germany (DE), United Kingdom (UK), and United States (US).

### 7. Pre-COVID-19 Sample Period

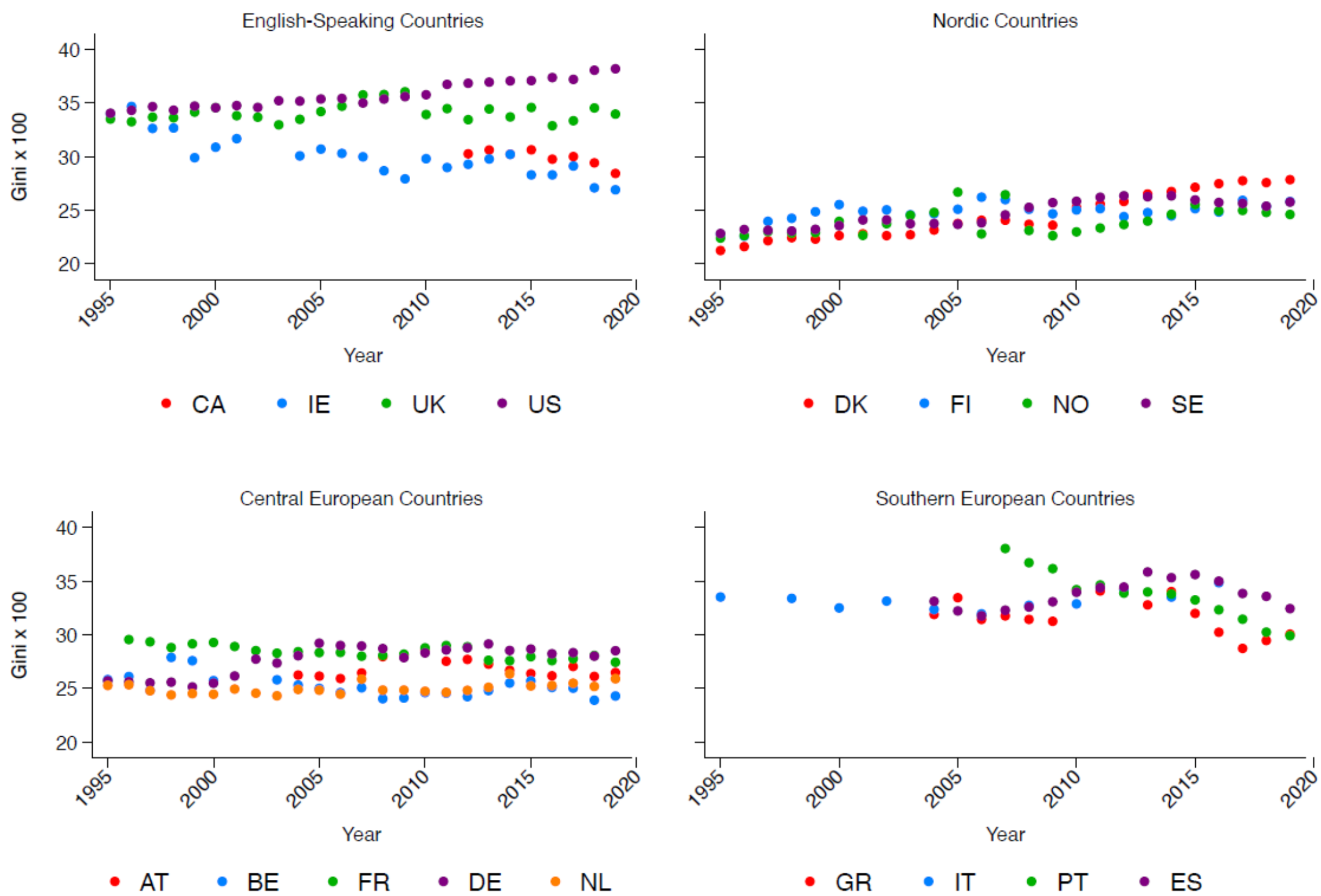


**Figure S38.** Cross-Country Trends in Disposable Income Inequality among the Working-Age Population, 1995-2019. Inequality is measured by the Gini coefficient times 100. Disposable income is the sum of market income from labor earnings, ordinary capital income, nongovernmental transfers, and governmental cash and near-cash transfer income, less tax payments inclusive of refundable credits. Income includes contributions from household members of all ages, and is equivalized using the Modified OECD scale. The sample used in the figure is the working-age population defined as those aged 25-60. Countries are grouped into English-speaking nations of Canada (CA), Ireland (IE), United Kingdom (UK), and United States (US); Nordic countries of Denmark (DK), Finland (FI), Norway (NO), and Sweden (SE); Central European countries of Austria (AT), Belgium (BE), France (FR), Germany (DE), and The Netherlands (NL); and Southern European nations of Greece (GR), Italy (IT), Portugal (PT), and Spain (ES).

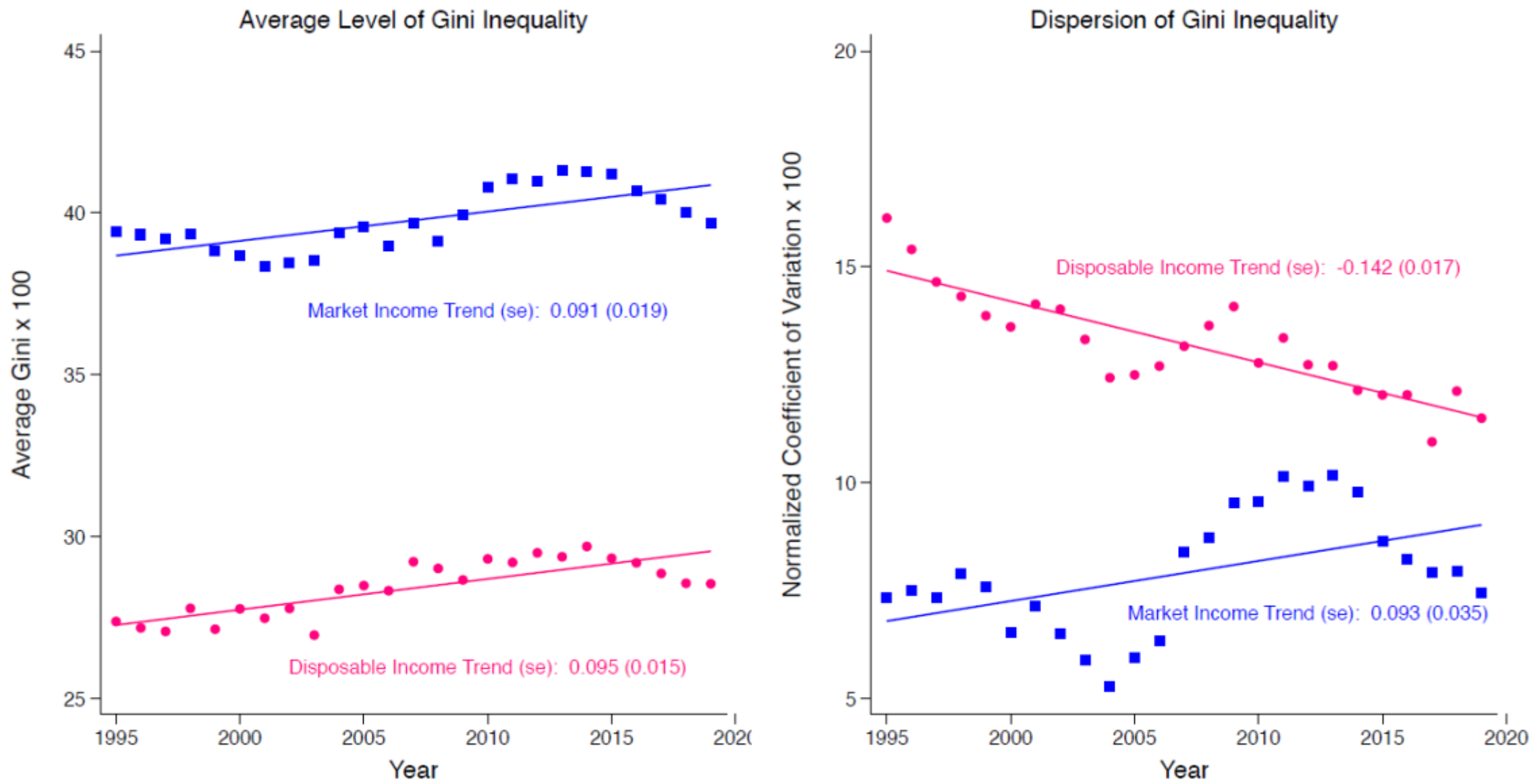


**Figure S39.** Average Level of and Convergence in Cross-Country Disposable and Market Income Inequality among the Working-Age Population, 1995-2019. Inequality is measured by the Gini coefficient times 100. Dispersion is measured by the normalized coefficient of variation of inequality ($NCV = \frac{CV}{1+CV}$) times 100, where *CV* is the ratio of the standard deviation to the mean, $CV = {}^{\sigma}/_{\mu}$. The test of convergence is represented by the coefficient and associated standard error (se) on the linear trend line through the *NCV* in the figure. Market income is the sum of labor earnings, ordinary capital income, and nongovernmental transfers. Disposable income is market income plus cash and near-cash governmental transfers less tax payments inclusive of refundable credits. Income includes contributions from household members of all ages, and is equivalized using the Modified OECD scale. Working-age population is defined as those aged 25-60.

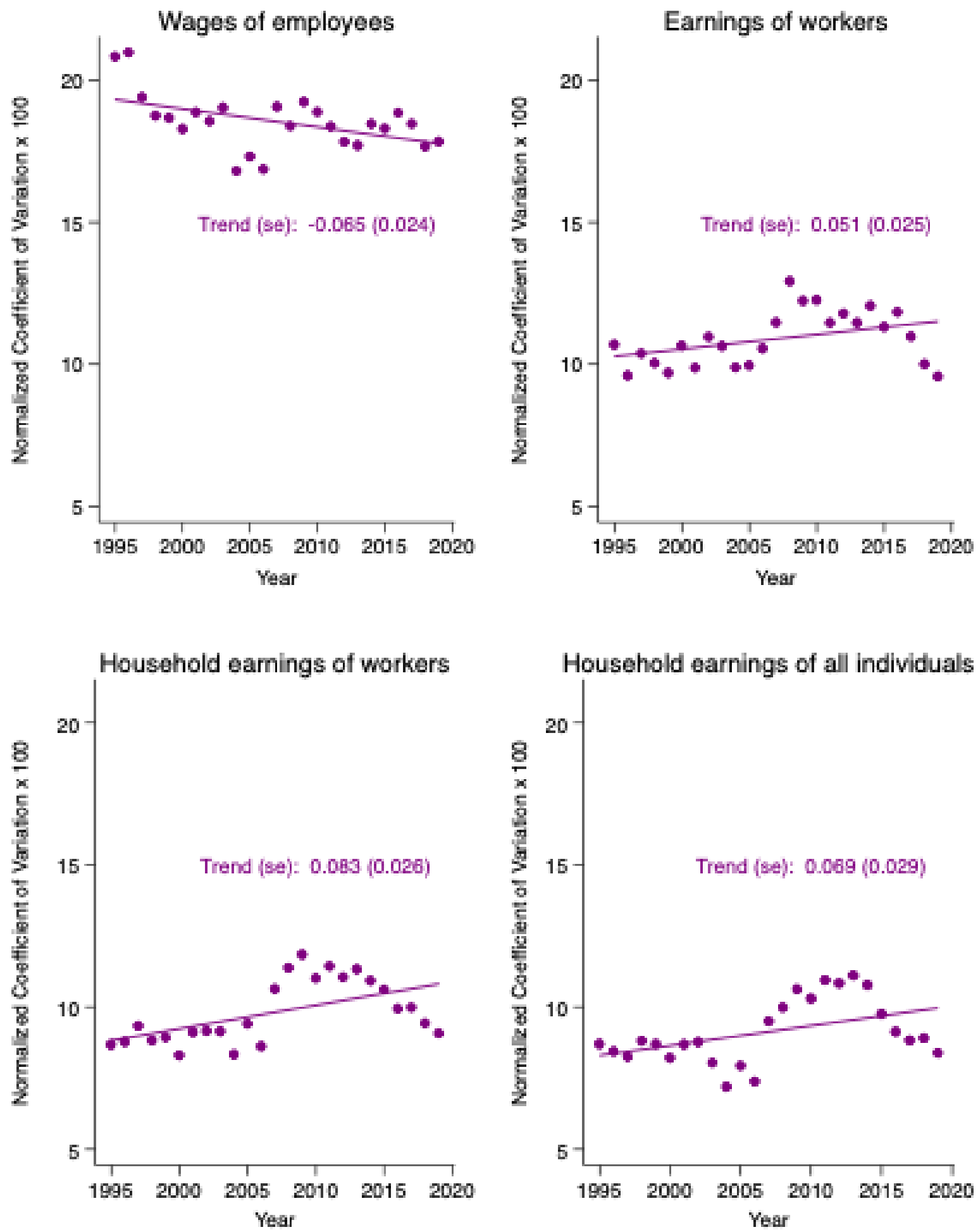


**Figure S40.** Tests of Convergence in Cross-Country Labor-Market Inequalities among Working-Age Population, 1995-2019. Inequality is measured by the Gini coefficient times 100. Dispersion is measured by the normalized coefficient of variation of inequality ($NCV = \frac{CV}{1+CV}$) times 100, where *CV* is the ratio of the standard deviation to the mean, $CV = {}^{\sigma}/_{\mu}$. The test of convergence is represented by the coefficient and associated standard error (se) on the linear trend line through the *NCV* in the figure. Wages of employees are average hourly earnings for employees. Earnings of workers are annual earnings of employees and self-employed workers. Household earnings of workers add up annual earnings of all persons who are employees or self-employed workers. Household earnings of all individuals include non-working households. Household earnings are equivalized using the Modified OECD scale. Working-age population is defined as those aged 25-60.

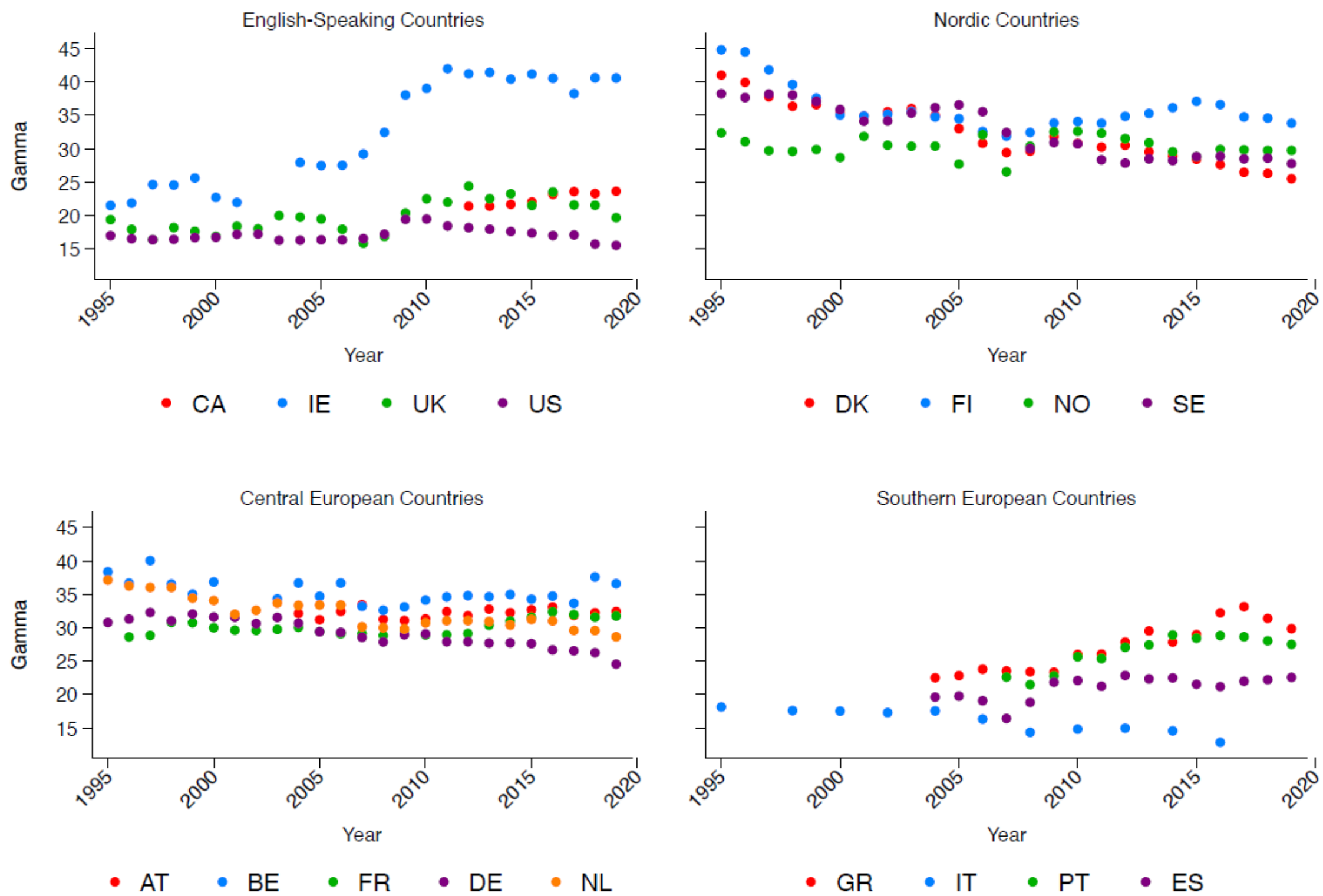


**Figure S41.** Cross-Country Trends in Redistribution from Market Income to Disposable Income for Working-Age Population, 1995-2019. Inequality is measured by the Gini coefficient. Gamma is 100 times the relative reduction in market income inequality to disposable income inequality defined as $\hat{\gamma}_{j,t}^{Gini} = 100 * (1 - \frac{\hat{I}_{j,t}^{Disp\ Gini}}{\hat{I}_{j,t}^{Market\ Gini}})$. Market income is the sum of labor earnings, ordinary capital income, and nongovernmental transfers. Disposable income is market income plus cash and near-cash governmental transfers less tax payments inclusive of refundable credits. Income includes contributions from household members of all ages, and is equivalized using the Modified OECD scale. Working-age population is defined as those aged 25-60. Countries are grouped into English-speaking nations of Canada (CA), Ireland (IE), United Kingdom (UK), and United States (US); Nordic countries of Denmark (DK), Finland (FI), Norway (NO), and Sweden (SE); Central European countries of Austria (AT), Belgium (BE), France (FR), Germany (DE), and The Netherlands (NL); and Southern European nations of Greece (GR), Italy (IT), Portugal (PT), and Spain (ES).

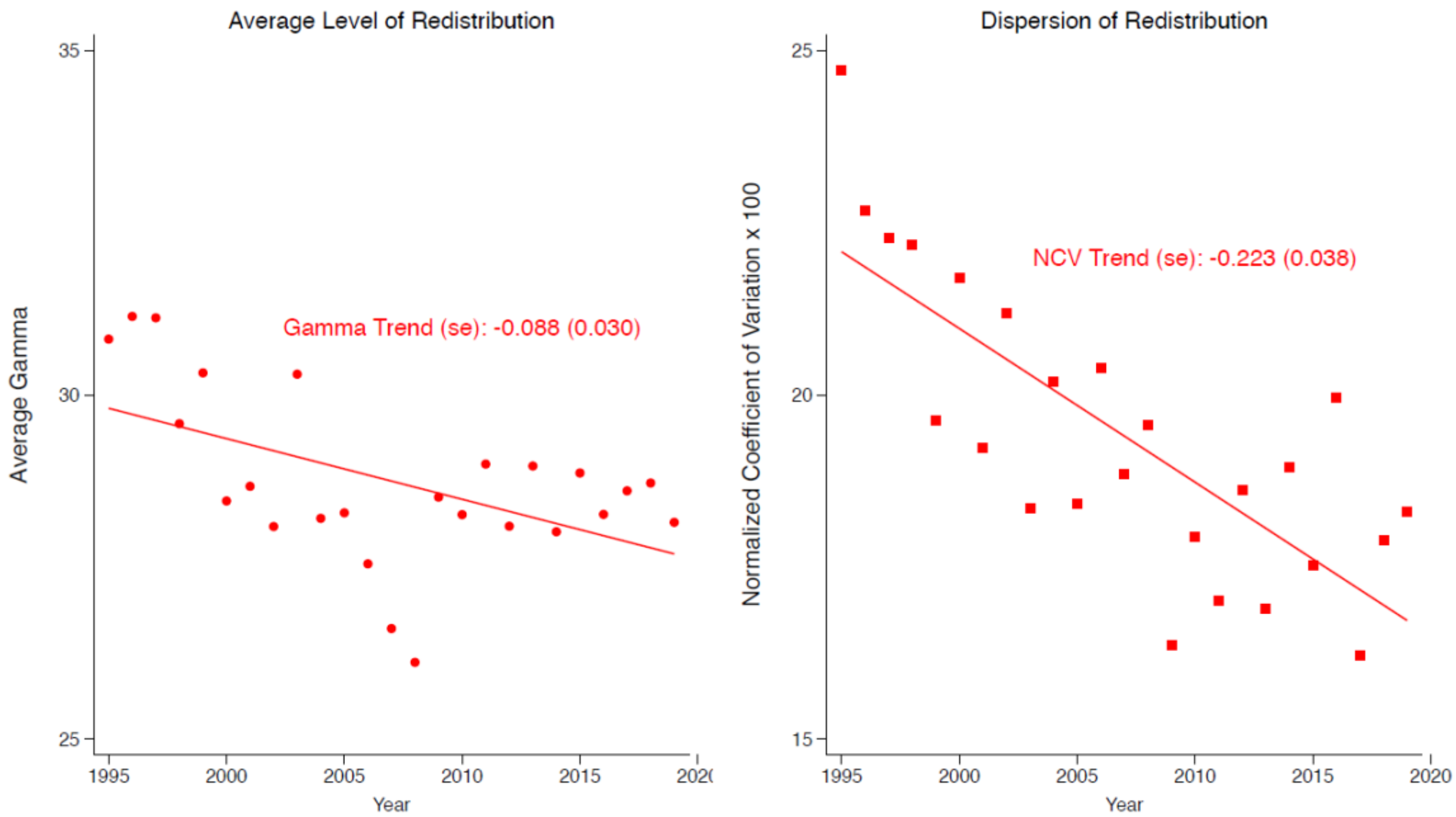


**Figure S42.** Average Levels of and Convergence in Cross-Country Redistribution from Market Income to Disposable Income among the Working-Age Population, 1995-2019. Average Gamma is the cross-country average of Gamma in Fig. 4, the latter defined as 100 times the relative reduction in market income inequality to disposable income inequality defined as $\hat{\gamma}_{j,t}^{Gini} = 100 * (1 - \frac{\hat{I}_{j,t}^{Disp\ Gini}}{\hat{I}_{j,t}^{Market\ Gini}})$. Inequality is measured by the Gini coefficient. Dispersion is measured by the normalized coefficient of variation of gamma ($NCV = \frac{CV}{1+CV}$) times 100, where *CV* is the ratio of the standard deviation to the mean, $CV = {}^{\sigma}/_{\mu}$. The test of convergence is represented by the coefficient and associated standard error (se) on the linear trend line through the *NCV* in the figure. Market income is the sum of labor earnings, ordinary capital income, and nongovernmental transfers. Disposable income is market income plus cash and near-cash governmental transfers less tax payments inclusive of refundable credits. Income includes contributions from household members of all ages, and is equivalized using the Modified OECD scale. Working-age population is defined as those aged 25-60.

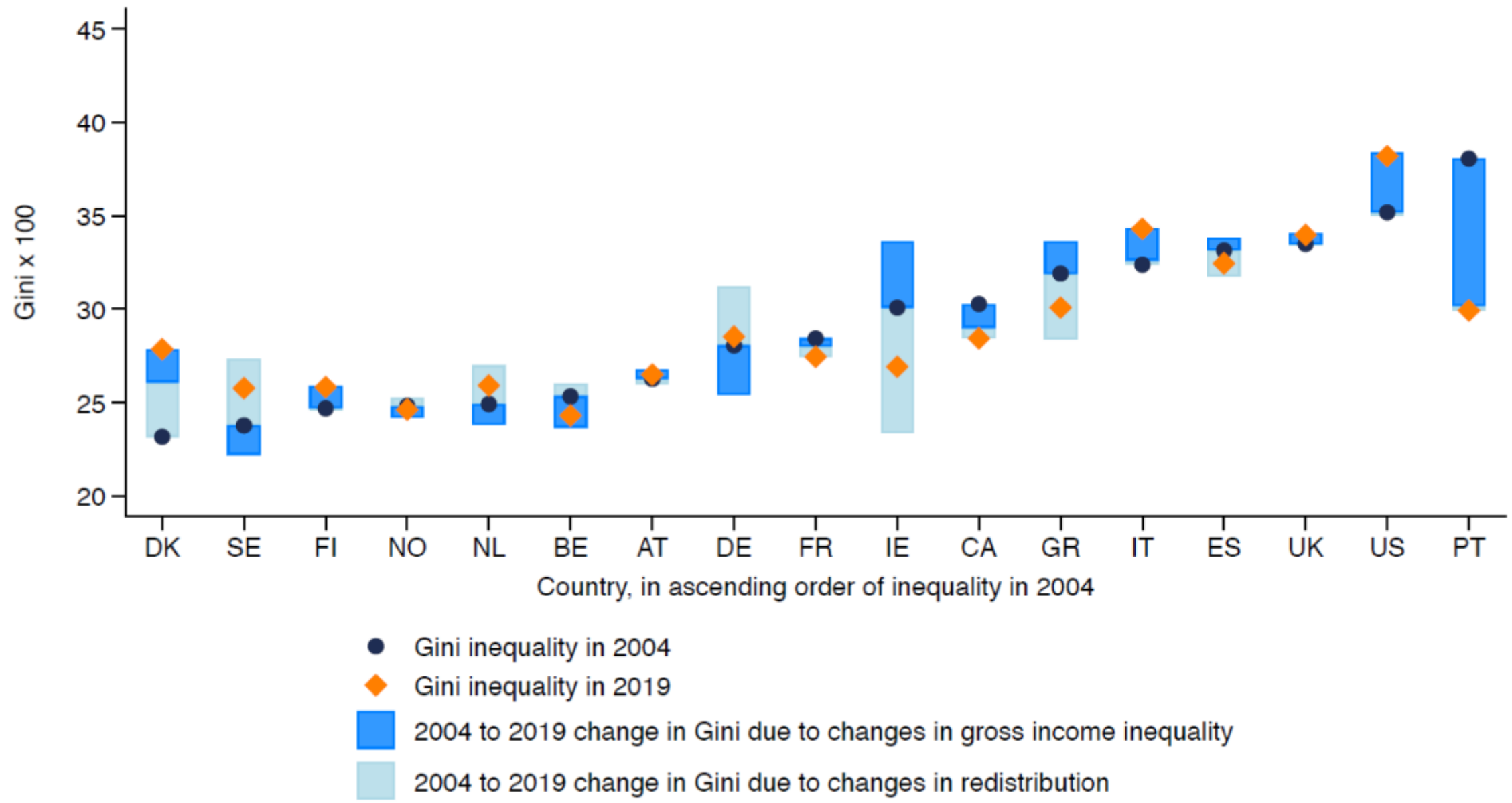


**Figure S43.** Decomposition of the Change in Disposable Income Inequality from 2004-2019 among the Working-Age Population. Inequality is measured by the Gini coefficient times 100. Market income is the sum of labor earnings, ordinary capital income, and nongovernmental transfers, and the mid-blue color bar reflects the change in market income inequality from 2004-2019. Disposable income is market income plus cash and near-cash governmental transfers less tax payments inclusive of refundable credits. Redistribution is the change in income inequality in moving from market income inequality to disposable income inequality as reflected in the light-blue color bar. See equation (4) in *Materials and Methods* for additional details. Income includes contributions from household members of all ages, and is equivalized using the Modified OECD scale. Working-age population is defined as those aged 25-60. Countries are listed in ascending order of initial 2004 disposable income inequality as Denmark (DK), Sweden (SE), Finland (FI), Norway (NO), The Netherlands (NL), Belgium (BE), Austria (AT), Germany (DE), France (FR), Ireland (IE), Canada (CA), Greece (GR), Italy (IT), Spain (ES), United Kingdom (UK), United States (US), and Portugal (PT). For CA, income in 2004 is from 2012; for IT, income in 2019 is from 2020; and for PT, income in 2004 is from 2007.

**8. Leave One Country Out**

| Drop Country: | Disposable Income | Market Income | Gamma |
|---|---|---|---|
| AT | -0.131 | 0.079 | -0.185 |
| | (0.016) | (0.033) | (0.033) |
| BE | -0.175 | 0.073 | -0.242 |
| | (0.015) | (0.030) | (0.033) |
| CA | -0.120 | 0.078 | -0.204 |
| | (0.015) | (0.031) | (0.035) |
| DE | -0.147 | 0.068 | -0.243 |
| | (0.016) | (0.032) | (0.033) |
| DK | -0.098 | 0.085 | -0.219 |
| | (0.015) | (0.033) | (0.033) |
| ES | -0.150 | 0.071 | -0.216 |
| | (0.016) | (0.032) | (0.038) |
| FI | -0.149 | 0.062 | -0.203 |
| | (0.014) | (0.031) | (0.032) |
| FR | -0.151 | 0.077 | -0.220 |
| | (0.014) | (0.032) | (0.032) |
| GR | -0.124 | 0.071 | -0.181 |
| | (0.016) | (0.031) | (0.035) |
| IE | -0.123 | 0.043 | -0.278 |
| | (0.014) | (0.027) | (0.037) |
| IT | -0.140 | 0.066 | -0.212 |
| | (0.015) | (0.031) | (0.030) |
| NL | -0.156 | 0.046 | -0.222 |
| | (0.016) | (0.031) | (0.034) |
| NO | -0.139 | 0.121 | -0.227 |
| | (0.014) | (0.030) | (0.035) |
| PT | -0.126 | 0.071 | -0.181 |
| | (0.016) | (0.030) | (0.035) |
| SE | -0.130 | 0.050 | -0.211 |
| | (0.017) | (0.032) | (0.035) |
| UK | -0.120 | 0.067 | -0.120 |
| | (0.018) | (0.033) | (0.040) |
| US | -0.168 | 0.049 | -0.149 |
| | (0.024) | (0.036) | (0.044) |
| Baseline | -0.138 | 0.069 | -0.206 |
| | (0.015) | (0.031) | (0.034) |

Table S1. Leave One Out Estimates of Convergence in Inequality and Redistribution. Each row is the coefficient on a linear trend with standard error in parentheses from leaving the indicated country out of the sample in computing the normalized coefficient of variation for the dependent variable in the column heading. The Baseline row is the corresponding coefficient and standard error with all countries included from Fig. 2 of the paper for disposable and market income inequality, respectively, and Fig. 5 of the paper for Gamma redistribution. There are 27 years used in estimation.

### 9. Beta Convergence in Inequality and Redistribution

| OUTCOME | Market Income Inequality | Market Income Inequality | Disposable Income Inequality | Disposable Income Inequality | Gamma Redistribution | Gamma Redistribution |
|---|---|---|---|---|---|---|
| Lag level | -0.036 | -0.198 | -0.034 | -0.186 | -0.031 | -0.116 |
| | (0.013) | (0.035) | (0.010) | (0.041) | (0.013) | (0.033) |
| Constant | -0.037 | -0.188 | -0.039 | -0.249 | 0.085 | 0.392 |
| | (0.013) | (0.032) | (0.014) | (0.058) | (0.047) | (0.118) |
| Observations | 366 | 366 | 366 | 366 | 366 | 366 |
| R-squared | 0.194 | 0.293 | 0.141 | 0.237 | 0.274 | 0.352 |
| Country Fixed Effects | No | Yes | No | Yes | No | Yes |
| Year Fixed Effects | Yes | Yes | Yes | Yes | Yes | Yes |

Table S2. Tests of Beta Convergence in Inequality and Redistribution. Tests based on Recentered Influence Function adjusted income series. Robust standard errors in parentheses. In each column the dependent variable is the change in the log of the variable and the key independent variable is the lag log level of the dependent variable. Inequality is measured by the Gini coefficient, with Gamma Redistribution being the relative reduction in Gini inequality for disposable compared to market income inequality. Models without country fixed effects are tests of absolute *beta*-convergence, and those with country fixed effects are tests of conditional *beta*-convergence.

**Additional References**


1. S. Jenkins, Accounting for inequality trends: decomposition analyses for the UK, 1971–86. Economica, 62, 29–63 (1995).
2. T. Piketty, E. Saez, Income inequality in the United States, 1913-1998. Quar. J. Econ, 118, 1-39 (2003).
3. Hardy, B. L., Krause, E., Ziliak, J. P., Income inequality in the United States, 1975–2022. Fiscal Studies, 45, 155–171. (2024).
4. J. Banks, R. Blundell, A. Bozio, J. Cribb, D. Green, J. Ziliak, Changing labour market and income inequalities in Europe and North America: A parallel project to the IFS Deaton Review of Inequalities in the 21st Century. Fisc. Stud., 45, 111-117 (2024).
5. Meyer, B., Mok, W., Sullivan, J. Household surveys in crisis. J. of Econ. Persp., 29, 199-226 (2015).
6. Brewer, M., Etheridge, B., O'Dea, C. Why are households that report the lowest incomes so well off?, The Econ. J., 127, F24-F49, (2017).
7. Bahk, S., Moffitt, R., Smeeding, T. Legacies of the War on Poverty. The ANNALS of the Amer. Acad. Polit. and Soc. Sci., 711, 38-56 (2024).
8. R. Barro, X. Sala-i-Martin, Convergence. J. Pol. Econ., 100, 223-251 (1992).
9. X. Sala-i-Martin, Regional cohesion: Evidence and theories of regional growth and convergence. Eur. Econ. Rev., 40, 1325-1352 (1998).
10. S. Durlauf, D. Quah, "The new empirics of economic growth" in Handbook of Macroeconomics, J. Taylor, M. Woodford, Eds. (North-Holland, 1999), pp. 235-308.
11. P. Johnson, C. Papageorgiou, What remains of cross-country convergence? J. Econ. Lit., 58, 129-175 (2020).
12. R. Benabou, "Inequality and Growth" in NBER Macroeconomics Annual, B. Bernanke, J. Rotemberg, Eds. (MIT, 1996), pp. 11-92.
13. D. Quah, Twin peaks: Growth and convergence in models of distribution dynamics. Econ. J., 106, 1045-1055 (1996).
14. Fuceri, D. $\beta$ and $\sigma$-convergence: A mathematical relation of causality, Econ. Let., 89, 212-215 (2005).
15. Young, A., M. Higgins, D. Levy, Sigma convergence versus beta convergence: Evidence from U.S. county-level data. J. Mon. Cred. Bank., 40, 1083-1093 (2008).
